# *Chaetomium*, *Chlonostachys,* and *Pseudogymnoascus* isolates from tomato tissues significantly suppress *Phytophthora infestans* in tomato

**DOI:** 10.1371/journal.pone.0335007

**Published:** 2025-10-24

**Authors:** Philemon Orwa, Theresa Kuhl-Nagel, Rosa Meinhold-Ernst, Arne Seyer, Johannes A. Jehle, Romano Mwirichia, Ada Linkies

**Affiliations:** 1 Julius Kühn Institute (JKI) - Federal Research Centre for Cultivated Plants, Institute for Biological Control, Dossenheim, Germany; 2 Leibniz Institute of Vegetable and Ornamental Crops (IGZ), Plant-Microbe Systems, Großbeeren, Germany; 3 Geisenheim University, Department of Crop Protection, Geisenheim, Germany; 4 University of Embu, Department of Biological Sciences, Embu, Kenya; Benemérita Universidad Autónoma de Puebla: Benemerita Universidad Autonoma de Puebla, MEXICO

## Abstract

Late blight is a disease whose causative agent is the oomycete *Phytophthora infestans.* It is one of the most destructive pathogenic oomycetes and a major challenge to global tomato production. The pathogen is difficult to manage because of its ability to evolve thereby evading host resistance. The aim of this study was to screen for potential antagonists of *P. infestans* using a combination of culture and microbiome-based approaches. Samples were collected from healthy and *P. infestans*-infected tomato plants grown in soil collected from two organic tomato growers in the Rhine-Main area in Germany. A total of 246 fungal isolates were screened for their antagonistic activity against *P*. *infestans*. Most of the isolates that exhibited *in vitro* antagonistic activity were from the genera *Penicillium, Trichoderma, Chlonostachys, Mortierella,* and *Pseudogymnoascus*. Following a stepwise *in vitro* screening strategy that accounted for growth features, ecological aspects, taxonomic data, potential health risks, commercial properties, and antagonistic efficacy, five fungal isolates were eventually selected for plant trials. *Chaetomium subaffine* showed the highest inhibitory effect against *P. infestans* across three trials whereby the percentage of diseased leaf area reduced by 90% compared to the control. *Chlonostachys* and *Pseudogymnoascus* spp. were effective in two trials, while *Trichoderma* and *Ctenomyces* spp. showed weak disease suppressive effects. In parallel, we characterized the fungal microbiome of the rhizosphere, phyllosphere, and endosphere from healthy and diseased tomato plants using ITS-rRNA sequencing. The fungal community differed significantly between the two soil origins, but *P. infestans* did not significantly influence fungal microbiota composition. Notably, 70% of our antagonistic fungi from the culture collection were detected in the tomato microbiome. This work identified isolates of *Chaetomium subaffine, Clonostachys sp.,* and *Pseudogymnoascus sp.* as potential biocontrol candidates promoting plant health. The findings highlight the importance of combined functional screening and microbiome profiling for identifying fungal antagonists.

## Introduction

Tomato is an important crop forming a vital component of the global diet, owing to its nutritional and health benefits [1]. In addition to other fungal pathogens such as *Alternaria solani*, *Botrytis cinerea*, and *Fusarium oxysporum* f. sp. *lycopersici* [2], late blight disease caused by *Phytophthora infestans,* is among the most devastating pathogenic oomycetes threatening global tomato production [3]. Studies indicate that *P. infestans* has become more aggressive over the past decades [4,5] and that the rapidly evolving genotypes are linked to severe epidemic global outbreaks [6]. Management costs and yield loss associated with late blight disease ranges between 3–10 billion United States Dollars per year globally [7,8].

Conventional approaches for managing *P. infestans* primarily utilize synthetic fungicides, which adversely impact the environment and may harm terrestrial and aquatic organisms [9]. The pathogen also poses the risk of fungicide resistance as it evolves rapidly [10]. In this regard, there are increasingly stringent regulations regarding the use of chemical-synthetic and copper fungicides. The European Union (EU) for instance, has heightened restrictions on the registration of chemical-synthetic pesticides through directives 2009/128/EC and (EU) 2019/782, aiming at lowering the risks associated with pesticide application on humans and the environment [11]. Therefore, there is an urgent need for more eco-friendly alternatives to chemical-synthetic and copper fungicides. Microbial antagonists promote plant growth and suppress plant pathogens through distinct mechanisms, including induced systemic resistance (ISR) [12], competition for space and nutrients [13], or the production of antimicrobial secondary metabolites [14]. The genera *Bacillus* and *Pseudomonas* are commonly found as microbial antagonists [15,16], while *Trichoderma* is among the most investigated and described fungal genera used as a biocontrol agent [17,18].

The soil microbiome includes many beneficial microorganisms and is an important source for plants to recruit their rhizosphere microbiome [19]. Soil microbiomes provide unique properties for plant pathogen control, also known as soil-borne legacy, which is beneficial to plants of the next generation thriving on the same soil [20]. However, plant pathogens can drive dysbiosis of the plant microbiome with consequences for plant health [21]. The rhizosphere, shaped by plant roots, is a complex habitat for diverse microorganisms, such as protozoa, mycorrhizal fungi, nitrogen-fixing bacteria, plant growth-promoting rhizobacteria (PGPR), and mycoparasitic fungi [22–25]. Fungi play multifaceted roles in the soil, including nitrogen fixation, and synthesis of plant growth–promoting hormones, they are natural biological agents against root pathogens and promoters of plant resilience under drought stress [26–28]. Additionally, fungi are key players in stabilizing soil organic matter by breaking down plant residues [29]. In recent years, there has been a growing emphasis on harnessing the diversity of soil fungi as a sustainable method to improve the quality of soil and boost crop productivity —an emerging strategy holding significant promise for the future of agriculture [30]. Several fungal species have been described with biocontrol potential against *P. infesta*ns [31]. Research on new or better biocontrol agents often focuses mainly on *in vitro* confrontation assay results as the basis for the selection of promising candidates, a majority of which do not meet the requirements for commercial use. The current work seeks to address the above-described research limitations by finding answers to the following questions. (i) Are the micro-compartments of tomato plants a suitable source for isolating fungal strains with effective biocontrol potential against *P. infestans*, which also meet selection criteria for later product development and registration? (ii) Does the microbiome structure differ between healthy and *P. infestans*-infected tomato plants grown in two soil origins, and can specific fungal groups be identified as key contributors to disease suppression? Therefore, we applied an integrated approach in the search for fungal antagonists against *P. infestans* by linking culture-dependent characterization to microbiome analyses and assessing the results in the context of tomato plant health. In the culture-dependent approach, we isolated and characterized potential fungal antagonists from diseased and healthy tomato plant samples in a stepwise selection procedure previously described by Köhl et al. [32] to select promising candidates for *ad planta* trials against *P. infestans*. The selection procedure incorporates commercial aspects, including growth on low-cost growth media, massive spore production, and evaluating potential health risks, in addition to antagonistic efficacy. In the microbiome approach, we characterized and compared the fungal microbiome of the phyllosphere, the endosphere, and the rhizosphere of diseased and healthy tomato plants grown in soils from different origins using high-throughput sequencing (HTS). Linking culture-dependent work to the microbiome provides deeper insights to characterize beneficial fungi for biocontrol of tomato late blight and other tomato diseases.

## Methods

### Soil sampling, tomato plant growth conditions, and inoculation with *P. infestans*

Tomato plants (*Solanum lycopersicum* `Red Robin´ cultivar) were grown in two different natural soils that were previously used for organic tomato cultivation in the greenhouse. No chemical-synthetic pesticides and fertilizers were applied previously and during the experiments. As in both soils tomato plants were grown in the previous growing season, these soils are enriched with organisms that are supportive of tomato plant health. The soils were collected from two different professional tomato growers in the Rhine-Main area at Domäne Mechthildshausen, Wiesbaden, 50°02′ N, 8°19′ E (soil A), and Solidarische Landwirtschaft Rüsselsheim, 49°58′ N, 8°25′ E (soil B) in February 2022. The contact to the growers and the corresponding field sites (soil A and B) was provided by Christian Fetzer, employee from the Landesbetrieb Landwirtschaft Hessen, a governmental educational and advisory institution of the State of Hesse, subordinate to the Hessian Ministry of Agriculture and Environment, Viticulture, Forestry, Hunting, and Home Affairs (HMLU). The growers gave permission to collect soil from their greenhouses for this study. Soil samples were taken at five positions throughout the greenhouse area with a depth of 20 cm and a mixed sample from all samples was used as substrate for the experiments. Eight tomato seeds were sown in soils from both locations (soil A and B) in each pot (size 8 x 8 x 8.5 cm) within 24 h after soil collection. Four seedlings for each treatment and soil origin were transferred to individual pots 14 days after sowing. Plants were grown at a temperature range of 19−22°C for subsequent inoculation with *P. infestans* (isolate 606 of JKI culture collection) as described in Drenker et al. [33]. Briefly, four 5-weeks-old plants each grown in soil A and soil B were spray inoculated with 7 ml of 2 x 10^4^ ml^-1^ zoospores per plant. Four plants grown in each soil were not inoculated and served as healthy controls. Scoring for disease symptoms (percentage symptomatic area) was performed 2 weeks after inoculation with *P. infestans*.

### Sample preparation for microbiome analysis and isolation of fungal antagonists

Samples for partial ITS amplicon sequencing and isolation of viable fungi were collected from the above-described tomato plants. Samples from leaves, roots, and rhizosphere soil were collected from the tomato plant. One plant was considered one replicate, in total four replicate plants per treatment were analyzed. Several plant tissues per micro-compartment were pooled as described in detail below. Plant samples were processed for ITS amplicon sequencing and isolation of fungal antagonists following the protocol of Wieland et al. [34], with some modifications, described as follows.

### Rhizosphere sample collection

The roots of each plant were shaken carefully to remove non-adhering soil. Five grams of soil adhering to the roots were transferred in sterile 50 mL centrifuge tubes containing 20 mL of sterile saline buffer (0.85% NaCl) and mixed vigorously by vortexing for 2 min. The roots (approximately 5 g), from which the rhizospheric soil had been dislodged were soaked in 20 mL of sterile saline buffer (0.85% NaCl) and mixed thoroughly by vortexing for 5 min to obtain the rhizoplane. Both solutions were combined to obtain the final rhizosphere sample. A total of 16 rhizosphere samples were obtained for microbiome analysis, four replicates from each soil origin, both from healthy and diseased plants. The remaining rhizosphere material from the four replicate plants was pooled for the isolation of fungal antagonists. A total of four rhizosphere samples were obtained for fungal isolation, one replicate from each soil origin, and from healthy and diseased plants.

### Endosphere sample collection

Roots (approximately 5 g) from rhizoplane extraction described above were sterilized with ethanol (75%; 2 min), sodium hypochlorite solution (50%; 2 min), and ethanol (75%; 1 min) and rinsed five times in sterile distilled water. Root samples (100–150 mg) were then homogenized with a sterile pestle and mortar in 20 mL of sterile saline buffer (0.85% NaCl) to obtain the final endosphere sample. In total, 16 endosphere samples were obtained for microbiome analysis, four replicates from each soil origin, and healthy and diseased plants. The remaining endosphere material from the four replicate plants was pooled for the isolation of fungal antagonists. A total of four endosphere samples were obtained for fungal isolation, one replicate from each soil origin, and from healthy and diseased plants. A sterility check was performed by placing a subset of the sterilized roots on PDA at 27°C for 7 days, and the absence of microbial growth ensured the sterility of the root surfaces.

### Phyllosphere sample collection

Samples for the microbiome analysis and isolation of leaf-associated fungi followed the protocol by Kassa Semagn [35]: Fresh leaf samples (100–150 mg) were harvested from each plant by pooling 4 portions obtained from different branches, cut into smaller pieces into the collection tubes and processed immediately. Notably, samples from plants infected with *P. infestans* were collected from leaf patches with healthy-looking samples adjacent to the diseased area. A total of 16 rhizosphere samples were obtained for microbiome analysis, four replicates from each soil origin, from both healthy and diseased plants. The remaining phyllosphere material from the four replicate plants was pooled for the isolation of fungal antagonists. A total of four phyllosphere samples were obtained for fungal isolation, one replicate from each soil origin, and from healthy and diseased plants.

### Collection of fungal isolates

Culturable fungi were isolated from the phyllosphere and rhizosphere of healthy and diseased plants grown in soil A and B. A total of eight samples were collected for fungal isolation, including four rhizosphere samples (as described above) and four phyllosphere samples as described above. Precisely, 100 µl of serially diluted samples (10^−1^ − 10^−3^) in sterile saline buffer (0.85% NaCl) were spread-plated onto potato dextrose agar (PDA) and oatmeal agar (OMA) supplemented with antibiotics (rifampicin (20 mg/ml) and streptomycin (50 mg/ml)). Plates were incubated at different temperatures (14, 21, and 28°C) to increase the diversity of the collection. The first assessment of fungal growth was conducted 7 days after plating and was based on differences in growth morphology, whereas the last assessment of fungal growth was conducted 14 days after plating. Fungal colonies that emerged on plates with different media, at varying temperatures, and from different tissue samples were isolated through a series of sub-culturing steps.

### Characterization of fungal isolates regarding their biocontrol potential

**Fig 1:**
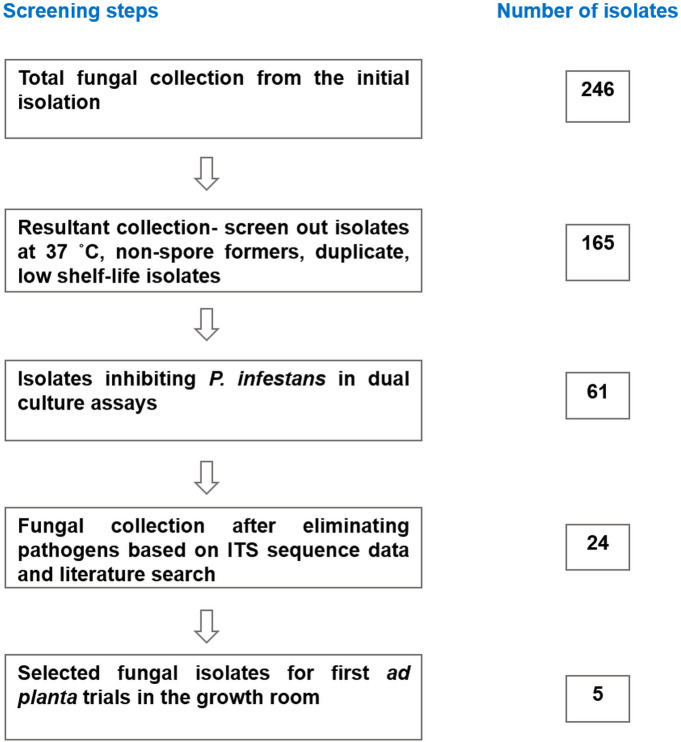
Stepwise screening procedure of the fungal isolate collection for potential microbial antagonists (Modified from: Köhl et al. [32]).

The present work adopted a stepwise screening approach developed by Köhl et al. [32] to select fungal candidates with promising characteristics as microbial antagonists of tomato pathogens (Fig 1). The isolated 246 fungi were examined under the microscope for their ability to form spores as this is a key parameter in the mass production of fungi for commercial purposes (S12 Table). The fungal isolates were further incubated at 37°C and those that grew in this temperature were eliminated as they could present potential risks to majorly humans and other mammals (S12 Table). Additionally, morphologically identical isolates that appeared repeatedly and those that lost viability were eliminated. The remaining 165 fungal isolates were subjected to dual-culture assays with *P. infestans* as the target plant pathogen in this work (S11 Table). A total of 61 fungal isolates that either formed inhibition zones when co-cultured with the pathogen on Petri dish or overgrew the pathogen were deemed to exhibit antagonistic effects, through antibiosis and matrix completion, respectively (S9 Table). The fungal antagonists were identified as potential biocontrol candidates and were molecularly characterized by sequencing the ITS locus, yielding an amplicon of approximately 560 bp (S9 Table). The already identified antagonists were further screened against *A. solani* (isolate 62028 of JKI culture collection) to reveal their broader antagonistic effects (S9 Table). Notably, the *in vitro* confrontation assays with *A. solani* only served as criteria for selecting the most promising antagonists, therefore, the pathogen was not used in the subsequent experiments. A literature search was performed and species that have previously been described as plant, human, or animal pathogens were eliminated, particularly *Penicillium* species which formed a higher proportion of our collection. The resultant collection of 24 fungal isolates were re-screened for *in vitro* inhibitory activity on *P. infestans* but this time on rye agar, which is the most suitable growth media for the pathogen (S10 Table). In the end, 5 fungal antagonistic candidates were selected for *ad planta* tests with *P. infestans* on tomato plants (Table 1). The criteria for selection of the 5 potential fungal antagonists included (i) literature evidence as a potential plant biocontrol agent: both broadly described genera, including *Trichoderma* and scarcely described genera, including *Pseudogymnoascus* were considered; (ii) ability to produce at least 4 x 10^5^ ml^-1^ spores on standard fungal media (PDA) within 21 days of incubation at 25 ˚C; (iii) no negative effects on healthy tomato plants following leaf application.

**Table 1:** Selection of fungal biocontrol candidates for *ad planta* trials with *P. infestans* on tomato plants. Data on isolates inhibiting *A. solani, in vitro* are included. The putative genera were determined by BLAST analysis after partial sequencing of the ITS locus (approximately 560 bp). Isolation parameters include media and temperature used for the isolation of fungi from plant samples. PDA: potato dextrose agar; OMA: oatmeal agar.

	NCBI BLAST close relative (ITS loci)				Isolation parameters			Inhibitory activity on rye agar and PDA
Isolate code	Genus match	Accession number	Sample source	Soil origin	Isolation temperature (°C)	Isolation Media	Spore count after 21 days of incubation at 25°C on PDA	*P. infestans*
Pf101	*Chaetomium* sp.	PX060209	Healthy rhizosphere	B	21	PDA	1.8 × 10^6^ spores/ml	yes
Pf 27	*Clonostachys* sp.	PX060190	Healthy rhizosphere	A	14	OMA	4.5 × 10^6^ spores/ml	yes
Pf 45	*Ctenomyces* sp.	PX060194	Diseased rhizosphere	A	21	PDA	2.2 × 10^6^ spores/ml	yes
Pf 23	*Pseudogymnoascus* sp.	PX060188	Diseased rhizosphere	A	21	OMA	2.6 × 10^6^ spores/ml	yes
Pf 131	*Trichoderma* sp.	PX060216	Diseased leaves	B	14	PDA	2.2 × 10^6^ spores/ml	yes

### *In vitro* dual-culture assays of fungal isolates against *P. infestans* and *A. solani*

Two mycelial discs (5 mm) of 7-day-old agar cultures of the fungal isolates grown on PDA and 7-day-old cultures of *P. infestans* grown on rye agar (200 g rye; 3 g glucose; 12 g agar; 1000 ml distilled water) were simultaneously inoculated 4 cm opposite each other on PDA in the first screening of 165 isolates (S11 Table) and on rye agar in the second screening of 24 isolates (S10 Table) in standard Petri dishes. The control plates were inoculated with *P. infestans* only. Three Petri dishes were prepared for each isolate. The plates were incubated at 25°C for 6–7 days, and fungal pathogen growth reduction indicated by inhibition zones around *P. infestans* was recorded (yes- inhibition zone present and no- inhibition zone absent). Fungal isolates that grew over the pathogen were also recorded as (yes) showing potential inhibitory effect. The screening for *A. solani,* precultured on PDA medium was performed in the same way as described for *P. infestans*.

### Testing disease suppressive effects of selected fungal isolates *ad planta*

Five fungal isolates, *Chaetomium* sp. (Pf101), *Trichoderma* sp. (Pf131), *Pseudogymnoascus* sp. (Pf23) *Chlonostachys* sp. (Pf27), and *Ctenomyces* sp. (Pf45), were selected from the characterized fungal collection for plant trials (Fig 1). The isolates were grown on PDA for 21 days at 25°C for spore production. Tomato seeds (`Red Robin´, Weigelt Samen) were sown in a 3:1 mixture of substrate (ProLine Potgrond, Klasmann-Deilmann, Germany) and sand, respectively, in the greenhouse. The seedlings were transplanted into planting pots (8 x 8 x 8.5 cm) containing the same soil mixture, 14 days after sowing. The plants were maintained in the greenhouse with demand-oriented watering and weekly fertilizer application using 0.2% (w/v) Hakaphos blau (Compo Expert, Münster, Germany). From each of the above-mentioned fungal candidates, a spore suspension of 4 x 10^5^ ml^-1^ was adjusted in distilled water. Five ml were sprayed on the leaves of 5-week-old plants. Plants were kept in the dark overnight. On the next day, the plants were spray-inoculated with 5 ml 2 x 10^4^ ml^-1^ conidia of *P. infestans.* The trays were covered with lids to ensure 100% humidity and left in the dark overnight. The plants were placed in the growth chamber (Grow Bank, Plant Climatics, Germany) set to 16 h/8 h light/darkness, day temperature 21°C, night temperature 16°C, day humidity 65%, and night humidity 75%. Percentage diseased leaf area was visually recorded 7 days after spraying with fungal biocontrol candidates as described by Drenker et al. [33]. Six tomato plants were used per treatment, and three independent replicates of the plant trials were done. All plant trials followed a randomized design.

### Molecular identification of isolates with inhibitory effects against *P. infestans*

Fungal DNA was extracted from 7-day-old cultures grown on PDA at 28°C using the DNeasy Plant Mini kit (Qiagen, Germany) following the manufacturer’s protocol. DNA quality was determined spectrophotometrically (NanoDrop 2000c; peqLab Biotechnologie GmbH, Erlangen Germany). The Internal Transcribed Spacer (ITS) region (approximately 560 bp) was amplified using primers ITS 1F- 5’-TCCGTAGGTGAACCTGCGG-3’, and ITS 4–5’-TCCTCCGCTTATTGATATGC-3’, as described in White et al. [36]. Briefly, a 25 µl PCR reaction mix was prepared for each sample, with reagents quantified as outlined by the manufacturer (Axon Labortechnik, Kaiserslautern). In the cases when PCR amplification was not successful, PCR reagents with a GoTaq® G2 Flexi DNA polymerase from Promega were used. The PCR cycling parameters were as follows: initial denaturation at 95°C for 3 min; 40 cycles at 95°C for 30 s (denaturation), 56°C for 30 s (annealing), and 72°C for 1 min (extension); final extension at 72°C for 5 min. PCR products were purified using the ExoSAP-IT™ PCR Product Cleanup Reagent (Thermo Fisher Scientific) and 10 µl was used for sequencing (StarSEQ, Mainz), pre-mixed with ITS-1F. The Basic Local Alignment Search Tool (BLAST) was used to identify potential genus matches by comparing the sequences of the isolates against the National Center for Biotechnology Information (NCBI) database (blastn, [37], and the most relevant hits based on the highest query coverage and percentage identity were recorded.

### Molecular identification of *Chaetomium* species

The actual species of the *Chaetomium* isolate was evaluated by sequencing three identification loci according to Linkies et al. [38]. Internal transcribed spacer (primers ITS-1 and ITS-4 [36]; RNA polymerase II second largest subunit (primers RPB2AM-1bf (5’-CCA AGG TBT TYG TSA ACG G-3’) and RPB2AM-7R (5’-GAA TRT TGG CCA TGG TRT CCA T-3’) [39]; β-tubulin (primers T1 (5’-AACATGCGTGAGATTGTAAGT-3’) and T22 (5’-TCTGGATGTTGTTGGGAATCC-3’) [40] were employed with the following PCR conditions: Initial denaturation: 5 min at 95°C, 40 cycles of denaturation: 30 s at 95°C, 30 s of annealing at 55°C and 45 s of extension at 72°C, and a final extension of 5 min at 72°C. The PCR product was visualized by 1% agarose gel electrophoresis. Cleaned PCR products using the ExoSAP-IT PCR Product Cleanup Reagent (Thermo Fisher, Berlin, Germany) were sequenced at StarSEQ (Mainz, Germany). Sequencing was performed in one direction and the species name was recorded as the highest BLAST hit. A consensus sequence of three separate PCR reactions was examined. The top BLAST hits for all three examined coding regions showed a 100% match to *Chaetomium subaffine* in both the ITS and β-tubulin regions, and a 99.63% match in the RNA polymerase II second largest subunit region.

### DNA extraction and library preparation for the fungal microbiome analysis

Total DNA was extracted from 0.25 mg of samples of the phyllosphere, endosphere, and rhizosphere from both soil origins and both healthy and diseased plants in four replicates (48 samples in total) using the DNeasy Powersoil Pro kit (Qiagen, Germany) following the manufacturer’s protocol. DNA yield and quality were checked photometrically (NanoDrop 2000c; peqLab Biotechnologie GmbH, Erlangen Germany) and samples were stored at −20°C. Library construction and high-throughput amplicon sequencing of ITS region with primers ITS1F (5’-CTTGGTCATTTAGAGGAAGTAA-3’ [41] and ITS2 (5’-GCTGCGTTCTTCATCGATGC-3’ [36] was carried out by StarSEQ GmbH (Mainz, Germany). Illumina adapter sequences were added in a 1-step PCR approach. Libraries were normalized and sequenced on the Illumina MiSeq platform with a v3 Reagent Kit in 2 x 300 nt paired-end mode.

### Analysis of ITS rDNA amplicon sequencing

Primers and adapters were removed from high-quality raw reads using the software Cutadapt (v.4.4, [42], implemented in the European Galaxy-Server [43](https://usegalaxy.eu/). Processing of paired-end reads was performed in R (v.4.2.1, [44] using the DADA2 pipeline (v.1.24.0, [45] following the adapted workflow for ITS sequences (DADA2 ITS Pipeline Workflow (1.8), https://benjjneb.github.io/dada2/ITS_workflow.html, accessed 25.1.2024). The obtained amplicon sequence variants (ASVs) were taxonomically assigned using NCBI BLAST (megablast, v.2.14.1, [37] with e-value 0.001 and the identity cut-off of 80% against the UNITE database (version 9, [46] containing ITS sequences in a Galaxy workflow as described in Fernandez-Grecco et al. [47]. Sequences identified as not fungal (= other eukaryotes) and sequences with less than five reads were removed from the dataset. This resulted in a final number of 2,124 ASVs and on average 124,032 quality reads per sample from 3,962,154 raw reads. Rarefaction curves indicated sufficient sequencing depth (S1 Fig). Raw reads are deposited at the Sequence Read Archive (https://www.ncbi.nlm.nih.gov/sra) under the BioProject accession number PRJNA1273108.

All scripts including detailed parameters and data to reproduce the figures for this study have been deposited in GitHub (https://github.com/TKuhl-Nagel/Phytophthora_challenged_tomato_microbiome.git).

### Data analysis and statistics

Statistical analysis and data handling for ASVs was performed using R (v.4.2.1, [44]) with the R packages “tidyverse” (v.1.3.2, [48], “ggplot2” (v.3.5.1, [49]), “ggtext” (v.0.1.2, [50]), “dplyr” (v.1.1.4, [51]), “tibble” (v.3.2.1, [52]), “stringr” (v.1.5.1, [53]), “ARTool” (v.0.11.1, [54]), “vegan” (v.2.6–6.1, [55]) and “phyloseq” (v.1.44.0, [56]). To account for uneven sequencing depth, the dataset was rarefied by 1000 times random subsampling to the lowest number of reads (18,628 reads) (S1 Fig) and all further analyses were performed with the rarefied dataset as recommended by Schloss [57]. Significant effects of micro-compartment, soil origin, and disease status on alpha-diversity indices (Richness, Evenness, Shannon, Simpson) were calculated using the non-parametric aligned rank ANOVA because residuals failed the normality criteria based on the Shapiro test in the previously performed 3-way-ANOVA (S1-S4 Tables). After normalizing the dataset to relative abundance (%) Bray-Curtis distance was used to estimate beta-diversity and visualized using nonmetric multidimensional scaling (NMDS). The effects of micro-compartment, soil origin, and disease status on the fungal community composition were tested using PERMANOVA (10,000 permutations). Relative abundance was computed for each ASV within each sample by dividing ASV counts by the total count per sample. Square bar plots were generated to represent the most abundant taxa (phylum, genus, and ASV level) based on their relative abundance in the whole dataset. Differences in their relative abundances based on soil origin were calculated for each micro-compartment using ANCOM-BC2 (v2.1.2, [58]) with p-value correction via the Benjamini-Hochberg method. The data set was filtered for rows with 0 ASVs prior to ANCOM-BC2 (Analysis of Compositions of Microbiomes with Bias Correction) to reveal differentially abundant taxa considering the two soil origins. ANCOM-BC2 also calculates the bias-corrected log-fold change (LFC) for each taxon between the two soil conditions to confirm that any taxon found only in one condition and completely absent in the other is a structural zero (sz) rather than rarely detected low-abundant taxa. LFC represents the ratio of the abundance of taxon between two conditions on a logarithmic scale. Negative LFC values indicate higher abundance in Soil A, while positive values indicate higher abundance in Soil B. ASVs classified as structural zero are exclusively present in one soil origin and are represented by strongly negative LFC (approaching -∞) and strongly positive LFC (approaching +∞). In order to compare the isolated living fungal species to the fungal community of the rhizosphere, we mapped the ASV sequences (~250 bp) to the isolate sequences (~500 bp) with 100% identity and at least 50% query coverage using NCBI BLAST. Afterwards, isolates were evaluated for their presence among the most abundant taxa and/or the differentially abundant taxa in the microbial community. Statistics for *ad planta* experiments and generation of plots were performed in R (versiolastn 4.4.2). Data on diseased leaf area (%) were presented as mean values ± standard error (SEM) computed from 6 biological replicates. The *ad planta* inhibitory effect of the fungal candidates against *P. infestans* was evaluated using a two-way analysis of variance (ANOVA) as the data was normally distributed according to Shapiro-Wilk test results. Significant differences between means were evaluated by Tukey’s test at P ≤ 0.05. Plant experiments were conducted in three independent repetitions, analyzed separately. Raw data from the three plant trials, and the associated R scripts including detailed parameters and data to reproduce the figures for this study have been deposited in GitHub (https://github.com/TKuhl-Nagel/Phytophthora_challenged_tomato_microbiome.git).

## Results

### Comparison of soil properties from Wiesbaden and Rüsselsheim in the the Rhine-Main area of Germany

**Fig 2:**
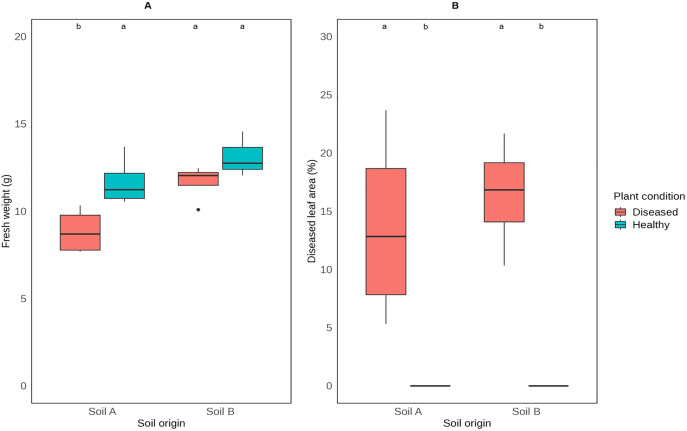
Effect of *P. infestans* on tomato plants grown in two different soil origins (A and B), in terms of (A) plant fresh weight and (B) diseased leaf area (%). The two soils were collected from two organic growers from Domäne Mechthildshausen, Wiesbaden (soil A) and Solidarische Landwirtschaft, Rüsselsheim (soil B). Plants were inoculated with *P. infestans* conidial suspension (2 × 10^4^ conidia/ml) 5 weeks after sowing. Tomato plants inoculated with distilled water served as the healthy control. Four tomato plants were used for each treatment (n = 4). Data were collected 14 days after inoculation with *P. infestans*. Means were compared with two-way ANOVA (p ≤ 0.01) followed by Tukey’s post-hoc test (*p* ≤ 0.05). Significant differences are shown by letters **(a, b)**.

Both soils were loam soils with comparable measures in terms of physical properties including humus level and organic carbon content (Table 2). Soil A had a slightly higher pH than soil B. While both soils were sufficiently supplied with macronutrients such as phosphorus, potassium, magnesium, and trace elements, they had low iron content. Tomato plants grown in soil A demonstrated a significant difference in weight between healthy and diseased conditions following 14 days of infection with *P. infestans* (Fig 2A), with diseased plants showing a significantly lower fresh weight. In soil B, there was no significant difference in fresh weight between healthy and diseased plants. However, healthy plants grown in both soil origins had comparable measures in weight. In addition, plants grown in both soils showed slight differences in the degree of symptom development upon inoculation with *P. infestans* (Fig 2B). The results indicate that the selected soils have comparable properties and are suitable as a resource for plant-beneficial fungi.

**Table 2:** Evaluation of the physiochemical parameters for soil A and soil B. The soil samples were analyzed according to VD-Lufa methods [59].

Soil sample	A	B	A	B
Soil origin	Wiesbaden	Rüsselsheim		
**Soil physical properties**	**Measured value**		**Evaluation**	
Soil type	Loam	Loam	Heavy	Heavy
pH value	7.5	7.2	Increased	Sufficient
Humus class	H2	H2	Slightly humic	Slightly humic
Organic carbon	0.6 – < 1.2	0.6 – < 1.2	Slightly humic	Slightly humic
**Main nutrients**	**Measured value** (mg/100 g)		**Supply rate**	
P_2_O_5_	119	44.0	High	High
K_2_O	34.5	36.0	Above requirement	Above requirement
MgO	145	39.4	High	Above requirement
**Trace elements**	**Measured value** (mg/kg)		**Supply rate**	
Boron	1.99	1.26	Above requirement	Above requirement
Copper	5.52	3.23	High	High
Iron	349	373	Low	Low
Zinc	30.5	10.8	High	High

### Characterization of the fungal culture collection derived from tomato rhizosphere and phyllosphere

**Fig 3:**
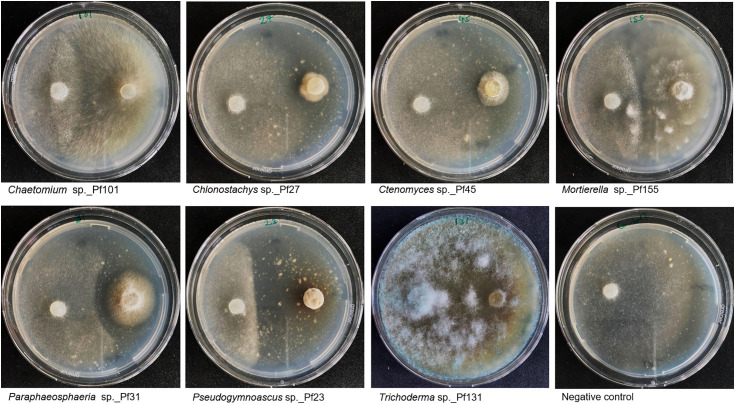
Activity of selected fungal isolates against *P. infestans.* Dual-culture assays showing representative fungal candidates (right part of each Petri dish) and *P. infestans* (left part of each Petri dish). Assays were performed on PDA with both fungal plugs placed 4 cm apart on Petri dishes. The results were recorded after 7 days at 25°C for n = 3 plates.

The isolation process of fungi from different samples yielded 246 isolates (Fig 1). In a stepwise screening approach described in the methods section, the number was reduced to 61 isolates (S9 Table) with promising characteristics as biocontrol agents against *P. infestans*. The *in vitro* antagonistic activity of the fungal isolates against *P. infestans* served as one selection criterion (Fig 3). Additionally, parameters that are advantageous for later product development and approval or registration as a biocontrol product were also taken into account, namely the inability to grow at 37°C, easy spore induction, and ability to survive on standard growth media. Out of the 61 identified isolates with antagonistic effects against *P. infestans*, the rhizosphere yielded 45 fungal isolates, of which 24 and 21 isolates derived from healthy and diseased rhizosphere, respectively. Another 16 isolates were recovered from the phyllosphere, of which eleven isolates derived from diseased phyllosphere compared to five isolates from healthy phyllosphere. All candidates were identified on the genus level by partial sequencing of the ITS locus.

The genus *Penicillium* constituted the largest share (27 isolates), followed by *Trichoderma* (6 isolates), *Agrostalagmus* (4 isolates), *Chlonostachys, Mortierella,* and *Pseudogymnoascus* (3 isolates each), (S9 Table). All other genera were only present once or twice. Based on literature research, potential health concerns and plant pathogenic isolates, such as *Penicillium*, *Fusarium*, and *Acrostalagmus* were removed, ending up with 24 remaining candidates (Table 1). Among the 24 fungi, *Mortierella* sp., *Pseudogymnoascus* sp., *Trametes* sp., and *Trichoderma* sp. are notable for their potential to inhibit both *P. infestans* and *A. solani.* Among the antagonists tested for *ad planta* disease suppressive effects on *P. infestans* included *Pseudogymnoascus* sp. and *Trichoderma* sp.. However, no further tests were performed with *A. solani* as most of the antagonists showed weak inhibition potential via *in vitro* confrontation assays. Intriguingly, a majority of the selected fungi (17 isolates) originated from samples of tomato plants grown in soil A.

### Fungal isolates show disease-suppressive effects *ad planta* against *P. infestans*

**Fig 4:**
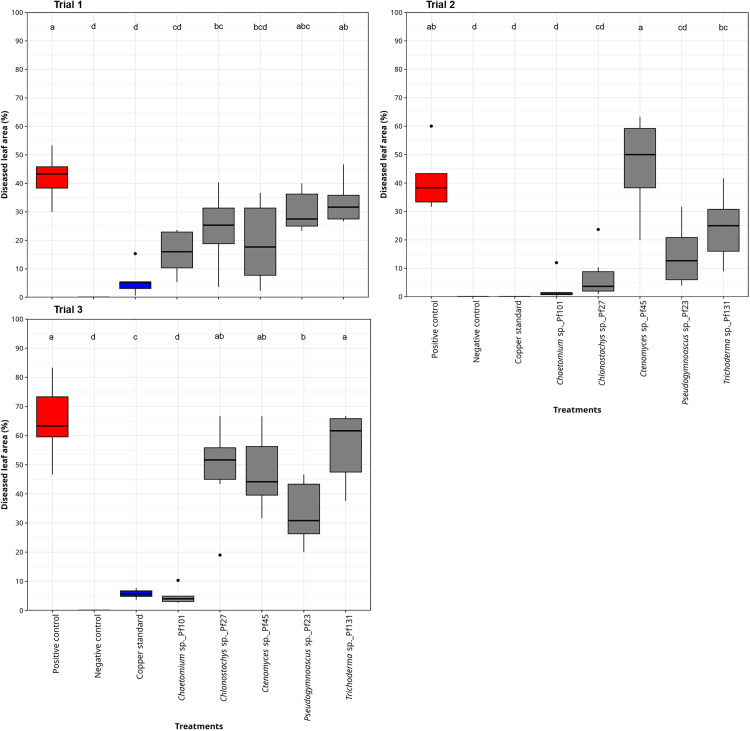
Efficacy of selected fungal isolates against *P. infestans* on tomato plants across three trials. Spore suspensions (4 × 10^5^ CFU/ml) prepared from *Chaetomium* sp*.* (Pf101), *Trichoderma* sp. (Pf131), *Pseudogymnoascus* sp. (Pf23), *Chlonostachys* sp. (Pf27), and *Ctenomyces* sp. (Pf45) were preventively applied on tomato leaves, 24 hours before inoculation with *P. infestans* (2 × 10^4^ ml^-1^ conidia). Tomato plants inoculated with distilled water served as the negative control (Negative_control). Tomato plants inoculated with *P. infestans* only served as the positive control (Positive_control). Tomato plants treated with Cuprozin progress (0.52%) and inoculated with *P. infestans* served as the chemical control (Copper standard). Six tomato plants were used for each treatment (n = 6). Percentage disease leaf area (%) was determined 7 days after inoculation with *P. infestans*. The letters above the boxes show significant differences across treatments (p ≤ 0.05) according to Tukey HSD tests following ANOVA on diseased leaf area data. The three trials represent three independent replicates.

Five of the remaining 24 fungal candidates were selected for exemplary tomato plant trials evaluating suppressive characteristics against *P. infestans*. *Pseudogymnoascus* sp. (isolate Pf23), *Chlonostachys* sp. (Pf27), and *Ctenomyces* sp. (Pf45), *Chaetomium* sp. (Pf101), and *Trichoderma* sp. (Pf131), were chosen based on visible mycelial growth towards the edges of the Petri dish on standard media, abundant spore production (at least 4 x 10^5^ ml^-1^ spores in standard fungal media (PDA)), and literature research. The isolates demonstrated varying levels of disease-suppressive potential in growth chamber plant trials (Fig 4). *Chaetomium* sp. (Pf101) significantly suppressed *P. infestans* across three independent replicates of the trial, as evidenced by the lower percentage of diseased leaf area compared to the positive control. *Chlonostachys* sp. (Pf27) significantly suppressed *P. infestans* in the first and second trials, whereas *Pseudogymnoascus* sp. (Pf23) significantly suppressed *P. infestans* in the second and third trials. *Ctenomyces* sp. (Pf45) was able to inhibit *P. infestans* once and *Trichoderma* sp. (Pf131) did not significantly inhibit symptoms of *P. infestans*, although the percentage of diseased leaf area was slightly lower compared to the positive control.

### Soil origin influences the fungal community in tomato plants

**Fig 5:**
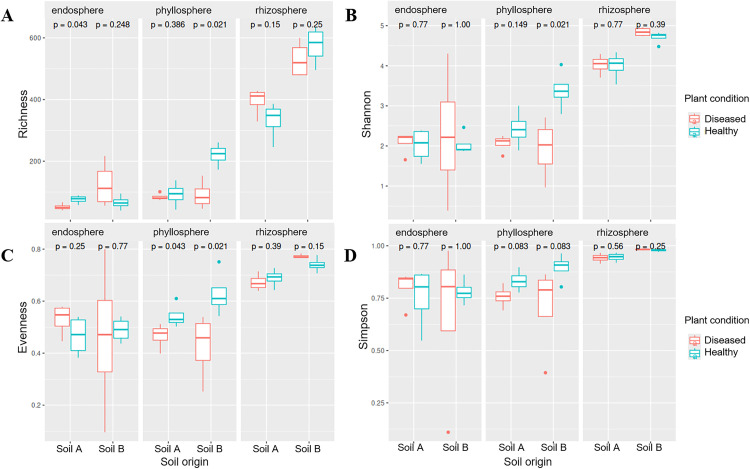
The alpha-diversity indices species richness (A), Shannon diversity index (B), evenness (C), and Simpson index (D) of the fungal microbiome in samples from phyllosphere, endosphere, and rhizosphere from tomato plants grown in two soil origins (soil A and soil B). Plants were grown in two different soil origins (A and B) and were inoculated with *P. infestans* (diseased, (turquoise plots) or remained untreated (healthy, red plots) 14 days before sampling was done. Four tomato plants were used for each treatment (n = 4). Values of alpha-diversity indices are shown, corresponding p-values are written above each comparison. Means were compared with a Kruskal Wallis test, with a significance threshold set at p ≤ 0.05.

The micro-compartment (endosphere, phyllosphere, rhizosphere), soil origin, disease status, and their interaction significantly influenced the alpha diversity of the fungal microbiome (ANOVA, Fig 5A-D, S1-S4 Tables). Rhizosphere samples had the highest alpha-diversity indices indicating the presence of many species and with a rather homogeneous distribution compared to endosphere and phyllosphere. The disease status of the plant had a significant influence on the alpha-diversity only in the phyllosphere (ANOVA, S1-S4 Tables), which shows significant differences in the within-sample diversity between healthy and diseased plants mainly in soil B (Fig 5A-C). Besides, *P. infestans* had an effect on fungal species richness in the endosphere of plants grown in soil A, where infection reduced fungal species richness compared to healthy samples (Fig 5A). These results show that leaf infection with *P. infestans* led to changes in the within-sample microbiome composition of the phyllosphere, although these changes are only significant in plants grown in soil B. In contrast, the endosphere and rhizosphere were mainly not significantly affected by microbial diversity after inoculation with *P. infestans*.

**Fig 6:**
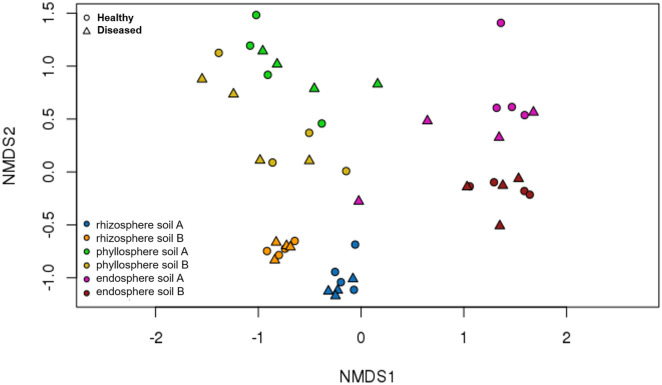
Microbial beta-diversity of different tomato micro-compartments visualized using an NMDS plot. The fungal microbiome composition was determined using Bray–Curtis community dissimilarities. Plants were grown in two soil origins (A and B) and inoculated with *P. infestans* 14 d prior to sample collection from the rhizosphere, phyllosphere, and endosphere of healthy and diseased plants. ANOSIM was used to determine significant differences (p ≤ 0.001, S5 Table).

The micro-compartment, soil origin, and their interaction were identified as the driving factors of the fungal ß-diversity (PERMANOVA p ≤ 0.001, S5 Table, Fig 6), whereas *P. infestans* infection had no significant influence on the fungal community composition. Phyllosphere samples clustered more distant from each other compared to rhizosphere and endosphere.

**Fig 7:**
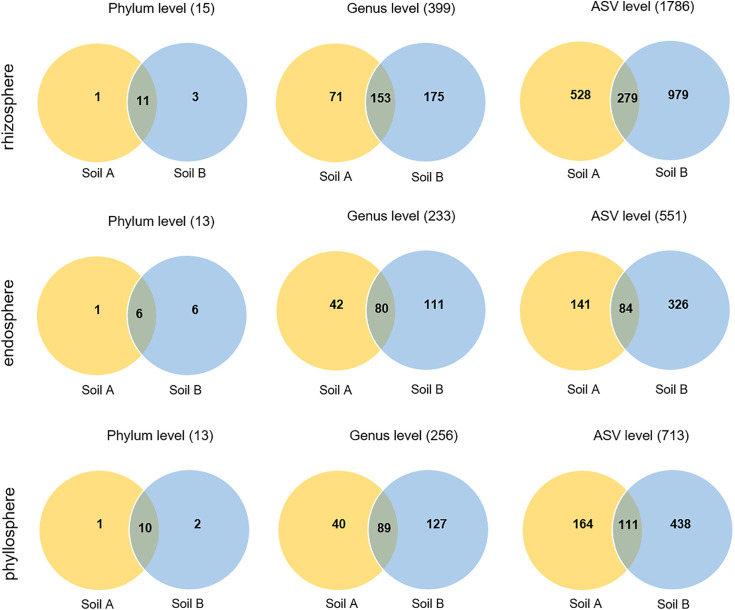
Fungal taxonomic distribution of the microbiome analyses at the phylum, genus, and amplicon sequence variant (ASV) levels in the rhizosphere, phyllosphere, and endosphere of soil A and soil B. The overlapping region represents taxa that are shared between soil A and soil B. Numbers inside each section represent the number of taxa at each level (phylum, genus, or ASV). The Venn diagram is based on the analysis of relative abundance with ANCOM-BC2.

Based on these results we focused on the effect of soil origin on the fungal microbial community in the different micro-compartments because beta diversity analysis showed no significant difference between diseased and healthy plants. In each micro-compartment, we identified shared and unique taxa between the two soils (Fig 7), using ANCOM-BC2 (Analysis of Compositions of Microbiomes with Bias Correction). Rhizosphere samples of the two soil origins had overall more taxa compared to the phyllosphere and the endosphere, which confirms previous results. Interestingly, samples from soil B harbour more taxa compared to soil A across the three micro-compartments, indicating a soil-specific effect on fungal composition at the phylum, genus, and amplicon sequence variant (ASV) levels. At the same time, a substantial number of taxa were shared between plant samples from soil A and soil B across the three taxonomic levels (phylum, genus, ASV).

**Fig 8:**
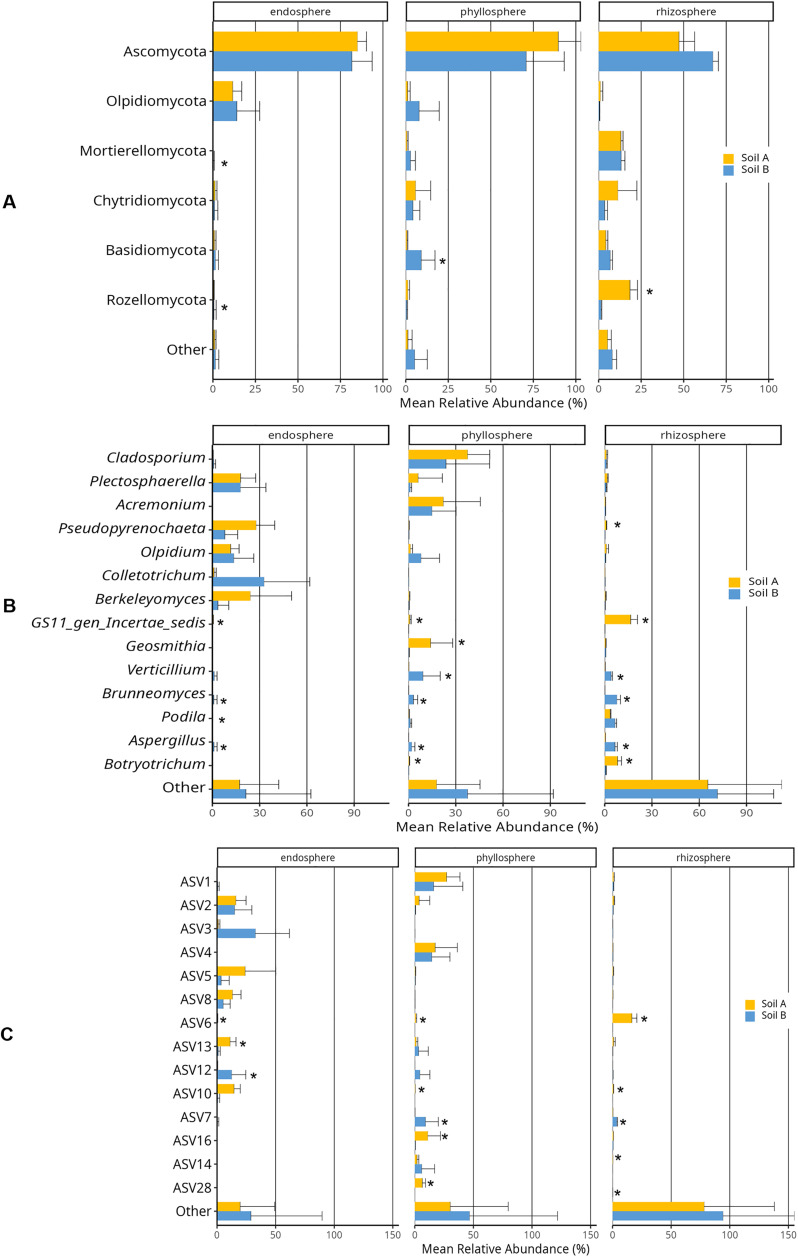
Diversity of the most abundant fungal taxa at phylum (A), genus (B), and (C) ASV levels between plants grown in soil A and soil B across the three micro-compartments (endosphere, phyllosphere, and rhizosphere). Others represent all taxa with less than 9%, 6%, and 5.5% abundance at phylum, genus, and ASV levels, respectively. Bars represent the mean relative abundance (%) of each phylum genus, and ASV. Error bars indicate standard deviation. Asterisks indicate a significant difference in abundance between samples from soil origins A and soil B inferred at p-value ≤ 0.05 according to Benjamini-Hochberg (BH) correction, which controls for false discovery rate (FDR) analyzed using ANCOM-BC2 (Analysis of Compositions of Microbiomes with Bias Correction).

Further analysis of the most abundant taxa in plant samples from the two soil origins showed that the phylum Ascomycota had the highest relative abundance in the whole data set (Fig 8A). Although other phyla showed variation between the two soil origins, only Basidiomycota, Rozellomycota, and Mortierellomycota significantly differed in abundance in individual micro-compartments. At the genus level, *Botryotrichum*, *Geosmithia*, *GS11_gen_Incertae_sedis,* and *Pseudopyrenochaeta* were more abundant in soil A, whereas *Aspergillus*, *Brunneomyces,* and *Verticillium* were more abundant in soil B in one or more micro-compartments (Fig 8B). *Cladosporium* and *Acremonium* were the top abundant genera in the phyllosphere, while *Colletotrichum* and *Pseudopyrenochaeta* were the most abundant group in the endosphere. The rhizosphere had a high proportion for an unknown genus, *GS11_gen_Incertae_sedis*.

The most abundant ASVs in the whole dataset were from three phyla, with a majority from Ascomycota, followed by Olpidiomycota, and Rozellomycota (Fig 8C, Table 3). The differentially abundant ASVs between the two soil origins across the three micro-compartments were linked to *Cladosporium*, *Geosmithia*, *Olpidium, Pseudopyrenochaeta, Verticillium,* and unknown genus (*GS11_gen_Incertae_sedis*) (Fig 8C, Table 3).

**Table 3:** Taxonomic affiliation of the top most abundant fungal ASVs in the microbiome of tomato plants in the whole data set. The ASVs were taxonomically assigned using NCBI BLAST (megablast, v.2.14.1) with an e-value of 0.001 and the identity cut-off of 80% against the UNITE database (version 9, [46]) containing ITS sequences in a Galaxy workflow.

ASVid	Taxonomic affiliation at phylum level	Taxonomic affiliation at genus level and potential species
ASV1	Ascomycota	*Cladosporium sphaerospermum*
ASV2	Ascomycota	*Plectosphaerella ramiseptata*
ASV3	Ascomycota	*Colletotrichum coccodes*
ASV4	Ascomycota	*Acremonium sclerotigenum*
ASV5	Ascomycota	*Berkeleyomyces rouxiae*
ASV6	Rozellomycota	GS11_gen_Incertae_sedis
ASV7	Ascomycota	*Verticillium dahliae*
ASV8	Ascomycota	*Pseudopyrenochaeta lycopersici*
ASV10	Ascomycota	*Pseudopyrenochaeta terrestris*
ASV12	Olpidiomycota	*Olpidium* sp.
ASV13	Olpidiomycota	*Olpidium* sp.
ASV14	Ascomycota	*Cladosporium halotolerans*
ASV16	Ascomycota	*Geosmithia* sp.
ASV28	Ascomycota	*Cladosporium sphaerospermum*

### Comparison of culture collection and fungal microbiome composition

In order to identify overlaps between the culture collection of living isolates and the microbiome analysis, we mapped ITS sequences of selected fungal isolates that demonstrated *in vitro* inhibitory effects against *P. infestans* to fungal ASVs in the microbiome of tomato plants grown in soils A and B. Of the 61 fungal isolates, 42 (*Penicillium* (21), *Trichoderma* (5), *Acrostalagmus* (4), *Clonostachys* (2), *Pseudogymnoascus* (2), *Cladosporium* (2), *Bionectriaceae* (2), *Ctenomyces* (2), *Chaetomium* (1) and *Humicola* (1)) showed 100% identity with the ASV sequences.

*Cladosporium* linked to isolates Pf179 and Pf242 from our culture collection was the only genus detected among the top 15 ASVs in terms of relative abundance (Table 3). Of the ASVs that matched our fungal collection, only ASV14 identified as *Cladosporium* sp. was differentially abundant in the rhizosphere between the two soil origins (Fig 8C, Table 3), with higher abundance in soil A. The matching ASVs were further evaluated for exclusive presence (also referred to as structural zeros) in plant samples from either soil origin. Intriguingly, ASVs identified as the genera *Mortierella, Ctenomyces,* and *Pseudogymnoascus* were exclusively present in the rhizosphere of plant samples from soil A, whereas ASVs identified as the genus *Chaetomium* were exclusively found in samples of plants grown in soil B (S6 Table). Phyllosphere and endosphere samples showed a similar pattern of exclusive taxonomic presence, with plants cultivated in soil A enriched with ASVs identified as the genus *Ctenomyces* and those from plants grown in soil B enriched solely with ASVs identified as *Chaetomium* (S7-S8 Table).

## Discussion

In times of increasing awareness of sustainable use of pesticides, microorganisms gain more and more importance as biocontrol agents of plant diseases [60,61]. Our study characterized cultivable fungi from various plant micro-compartments as the initial step towards discovering fungal biocontrol agents against *P. infestans*. Additionally, we employed a fungal microbiome analysis to evaluate the potential importance of our fungal candidates within the microbiome. The initial number of 246 isolates obtained from the phyllosphere, endosphere, and rhizosphere of tomato plants was reduced to 24 potential biocontrol candidates remaining for plant trials by utilization of the step-wise screening procedure described by Köhl et al. [32]. The multi-step framework employs several steps to screen a high number of isolates for candidates fulfilling requirements for antagonists in commercial use, for example regarding efficacy, ecology, production, safety, and environmental risks. Schisler and Slininger [62] also included the above commercial parameters to select viable bacterial antagonists against *Gibberella pulicaris,* a potato dry rot causing pathogen.

The rhizosphere yielded more candidate isolates than the phyllosphere and endosphere, which concurs with previous findings that described the rhizosphere as a highly competitive habitat and a stable reservoir for beneficial microbes in terms of pathogen suppression, nutrient acquisition, and host defense functions [24,63]. Additionally, as a nutrient-rich environment, partly caused by plant root exudates [64], the rhizosphere supports a diverse microbial community, including beneficial fungi with potential biocontrol characteristics. Low microbe numbers recovered from the phyllosphere are linked to the specific nature of this habitat which is characterized by highly varying conditions that support only the survival of a limited number of microbes [65]. Recovering more fungal candidates from diseased phyllosphere samples suggests the enrichment of fungal microbial antagonists in pathogen-prevalent micro-compartments. Phyllosphere-associated microbes have been described with various functions, including plant growth promotion and pathogen inhibition [66], and nitrogen fixation [67], and can be causative agents for plant diseases [66]. The genera *Penicillium, Trichoderma, Chlonostachys, Mortierella,* and *Pseudogymnoascus* dominated our collection of potential microbial antagonists based on *in vitro* tests. A majority of these fungal groups have been adequately described as potential biocontrol agents. Culture filtrates of *Penicillium griseofulvum* demonstrated inhibitory effects on the growth of *P. infestans in vitro* [68]. But since *Penicillium* [69] species have been frequently reported as mycotoxin producers or as otherwise harmful in regard to food safety and consumer health [70], all *Penicillium* sp. isolates were eliminated from subsequent steps at an early screening stage. *Trichoderma* has been broadly investigated as a BCA owing to its ability to thrive in diverse soil habitats and outcompete pathogens for space and nutrients among other biocontrol mechanisms [71]. *Trichoderma* is also linked to other plant-beneficial functions such as phosphate solubilization and could be vital for the overall fitness of tomato plants through plant growth promotion [72]. Particular strains of *Chlonostachys rosea* [73], a tomato stem endophyte, have been reported to significantly reduce early blight caused by *A. solani* [74] and *B. cinerea* [75] in tomatoes. Products based on *Trichoderma* sp. [76] and *C. rosea* [77] are approved as fungicides in Europe. Moreover, there is limited information on the isolation of *Pseudogymnoascus* species from tomato plants, but this fungal genus is among the most abundant taxa in tomato root microbiome through amplicon sequencing [78].

While *in vitro* effects of certain microbial species or strains may not necessarily be reflected in the *in vivo* effects, the five selected fungal candidates tested in *ad planta* assays showed varying disease-suppressive efficiency against *P. infestans*. The candidate antagonists from *Chaetomium* sp., *Chlonostachys* sp., and *Pseudogymnoascus* sp. demonstrated significant disease-suppressive effects in at least two separate plant trials, validating the feasibility of our approach in identifying new or improved biocontrol agents. It is worth noting that the findings on *Pseudogymnoascus* contribute substantially to the knowledge of this genus in the field of plant pathology as a potential biocontrol agent. *Chaetomium* species, including *Chaetomium cochliodes* and *Chaetomium aureum,* are known to produce antifungal secondary metabolites against tomato pathogens such as *A. solani*, *B. cinerea*, and *P. infestans* [38,79]. However, other species of *Chaetomium* induce human pathogenic effects linked to systemic infections and allergic reactions [80]. Our isolate was confirmed to be *Chaetomium subaffine* after sequencing three identification loci (as described in the methods). To the best of our knowledge, there is currently no data on potential health or environmental risks associated with *C. subaffine.* Liu et al. [81] revealed the potential mycolytic effects of *C. subaffine* LB-1 on phytopathogenic fungi *B. cinerea* and *A. solani* by altering their cell membrane and cell wall. Reduced biocontrol efficacy by other groups, including *Trichoderma sp*. (Pf131) and *Ctenomyces sp*. (Pf45) across trials could be linked to environmental or procedural effects. For instance, soil-originated beneficial microbes such as *Trichoderma* when introduced to the leaves and *vice versa* may not survive or could cause negative interactions with the native community of microorganisms on the leaf reducing the efficacy [3]. Further processing steps such as drying or adding stabilizers might surely change the outcome but have not been investigated here. Different application methods such as the use of soil additives and fertilizers or a regular leaf application allowing for preventive and curative effects might also increase efficacy [82] but have not been tested yet.

High-resolution microbiome profiling techniques have improved the research on cultivable fungal species with plant-beneficial effects, providing crucial data for the targeted application of antagonists to ensure improved efficacy [83,84]. One example of this approach is demonstrated by Sahu et al. [85] who integrated culture methods and metabarcoding to profile the microbiome of the rice phyllosphere, revealing the key microbial communities for blast pathogen control. Similarly, we detected the presence of some of our fungal candidate antagonists for *P. infestans,* including *Cladosporium* among the highly abundant taxa and differential taxa in the tomato plant microbiome dataset. Although some *Cladosporium* species are plant pathogens [86,87], there are plant-beneficial species promoting growth [88,89]. This genus could reflect the core microbiome which has received a lot of interest in recent years, with multiple studies revealing core microorganisms, including bacteria, fungi, and archaea playing different roles in various plant hosts [90–92]. The dominance of specific fungal taxa, for instance, the exclusive presence of *Mortierella, Ctenomyces,* and *Pseudogymnoascus* in the rhizosphere samples from soil A and *Chaetomium* from rhizosphere samples in soil B may be a reflection of their beneficial activities to ensure a healthy plant microbiome [83]. In the rhizosphere, root exudates released due to pathogen invasion trigger plants to recruit microorganisms that are involved in specific functions, including direct suppression of the pathogens [93,94]. The endosphere represents the internal phytomicrobiome enriched with specifically adapted microorganisms that interact intimately [95]. The phyllosphere is linked to the atmosphere and interacts continuously with the air microbiome [96]. The fact that the fungal candidate antagonists are naturally present within the tomato plant and are effective against *P. infestans* highlights their potential as BCAs [97,98]. Therefore, knowledge of the ecological role of fungal biocontrol candidates in their native microbiomes can provide insights into the selection of improved and effective fungal agents against *P. infestans*.

Enhanced knowledge of how pathogen infection of a host plant alters soil microbial communities may pave the way for the use of microbial antagonists in the long-term management of plant diseases [99]. Therefore, our approach to microbiome characterization provides a more complete picture of how *P. infestans* and distinct soil sources may influence the composition of the tomato fungal community. Although *P. infestans* did not alter the overall fungal microbiome composition of tomato plants, significant changes in fungal alpha diversity were evident in specific micro-compartments, particularly the phyllosphere. Previous research on *P. parasitica* (closely related to *P. infestans*) revealed changes in the bacterial microbiota in tomato roots upon infection, whereas the fungal microbiota remained relatively stable [100]. These changes in fungal composition could also be attributed to distinct soil physiochemical parameters as we cultivated tomatoes in soils from two different locations in the Rhine-Main area [101]. Studies have shown that pH plays a key role in determining the composition of bacteria and fungi in the rhizosphere and bulk soil [102,103]. Also, soils with sufficient nutrient amounts provide optimal habitat for beneficial microbial communities to outperform pathogens [104].

Taken together, we present a strategic approach that enhances the likelihood of identifying fungal candidates with both strong biocontrol potential and commercial viability. The tested fungal biocontrol candidates, including *Chaetomium* sp., *Chlonostachys* sp., and *Pseudogymnoascus* sp. isolated from different micro-compartments of tomato plants grown in two soil origins inhibited *P. infestans* and *A.solani in vitro* and reduced *P. infestans-*related disease symptoms *ad planta*. We have also demonstrated that *P. infestans* infection of tomato plants influences the fungal community composition at the micro-compartment level, particularly on the phyllosphere rather than on the overall plant microbiome. However, the effect of soil origin on fungal community composition is significant considering the overall microbiome of tomato plants. The high abundance and differential abundance of specific genera, in our case *Cladosporium* indicates the potential beneficial role of this genus in tomato plant health as initial treatment showed no negative effects on the plant. In subsequent steps, we plan to test the suppressive effects of our *Chaetomium subaffine* in open-field plant trials. Other fungal candidates with variable efficacy against *P. infestans* will be subjected to optimization steps and used as consortia. Lastly, we intend to incorporate both curative and preventive application methods in future plant trials to achieve better disease-suppressive effects from the fungal candidates in a controlled growth room and greenhouse conditions. These findings improve our knowledge of the link between fungal biological control agents and plant microbiome, providing valuable ecological data in identifying effective antagonists suited for sustainable control of *P. infestans*.

## Supporting information

S1 Table Non-parametric aligned-rank ANOVA results for species richness using the ARTool package (v.0.11.1). (DOCX)

S2 Table Non-parametric aligned-rank ANOVA results for Shannon using the ARTool package (v.0.11.1). (DOCX)

S3 Table Non-parametric aligned-rank ANOVA results for species evenness index using the ARTool package (v.0.11.1). (DOCX)

S4 Table Non-parametric aligned-rank ANOVA results for the Simpson index using the ARTool package (v.0.11.1). (DOCX)

S5 Table Permutation test results from Adonis2 for beta diversity analysis with Bray-Curtis dissimilarity. (DOCX)

S6 Table ANCOM-BC2 results of differential abundance of fungal ASVs between soil A and soil B in rhizosphere samples based on Log Fold Change (LogFC) and structural zero analysis. (DOCX)

S7 Table ANCOM-BC2 results of differential abundance of fungal ASVs between soil A and soil B in phyllosphere samples based on Log Fold Change (LogFC) and structural zero analysis. (DOCX)

S8 Table ANCOM-BC2 results of differential abundance of fungal ASVs between soil A and soil B in endosphere samples based on Log Fold Change (LogFC) and structural zero analysis. (DOCX)

S9 Table Total collection of fungal isolates inhibiting *P. infestans*, *in vitro.* (DOCX)

S10 Table Collection of 61 identified fungal biocontrol candidates inhibiting *P. infestans* after eliminating potential plant and human pathogens based on literature search. (DOCX)

S11 Table Collection of 165 unidentified fungal isolates after eliminating those growing at 37˚C and non viable isolates. (XLSX)

S12 Table Collection of 246 initial fungal isolates recovered from tomato plant tissues. (XLSX)

S1 Fig Rarefaction curve of observed species richness after sequencing of ITS rRNA gene from the rhizosphere, endosphere, and phyllosphere samples of tomato plants grown in soil origins A and B. (DOCX)
