# Supplementary material for: Chaetomium, Chlonostachys, and Pseudogymnoascus isolates from tomato tissues significantly suppress Phytophthora infestans in tomato
Source: PLoS One. 2025 Oct 24;20(10):e0335007. doi: 10.1371/journal.pone.0335007 (PMC12551835; doi:10.1371/journal.pone.0335007)
Supplement: S2 Table — (DOCX) [file pone.0335007.s002.docx]

*Chaetomium*, *Chlonostachys,* and *Pseudogymnoascus* isolates from tomato tissues significantly suppress *Phytophthora  infestans* in tomato

Philemon Orwa^1^, Theresa Kuhl-Nagel^2^, Rosa Meinhold-Ernst^1^, Arne Seyer^1,4^, Johannes A. Jehle^1^, Romano Mwirichia^3^, Ada Linkies^1*^

^1^ Julius Kühn Institute (JKI) - Federal Research Centre for Cultivated Plants, Institute for Biological Control, 69221 Dossenheim, Germany

^2^ Leibniz Institute of Vegetable and Ornamental Crops (IGZ), Plant-Microbe Systems, Großbeeren, Germany

^3^University of Embu, Department of Biological Sciences, 6-60100 Embu, Kenya

^4^Geisenheim University, Department of Crop Protection, 65366 Geisenheim, Germany

* Corresponding author

ada.linkies@julius-kuehn.de

**S2 Table.** **Non-parametric aligned-rank ANOVA results for Shannon using the ARTool package (v.0.11.1).** The effects of plant condition, microcompartment, and soil origin, and their interactions on the Shannon index are shown. Significant effects are shown at p < 0.05.

| Analysis of Variance of Aligned Rank Transformed Data  Table Type: Anova Table (Type III tests)  Model: No Repeated Measures (lm)  Response: art(Shannon) | | | | | |
| --- | --- | --- | --- | --- | --- |
|  | Df | Df.res | F value | Pr(>F) | Signif. level |
| Soil.origin | 1 | 36 | 13.51977 | 0.00076486 | *** 0 |
| Plant.condition | 1 | 36 | 3.67579 | 0.06317033 |  |
| Microcompartment | 2 | 36 | 40.72452 | 5.7037e-10 | *** 0 |
| Soil.origin:Plant.condition | 1 | 36 | 0.81046 | 0.37396614 |  |
| Soil.origin:Microcompartment | 2 | 36 | 2.98010 | 0.06344094 |  |
| Plant.condition:Microcompartment | 2 | 36 | 7.10604 | 0.00250552 | ** 0.001 |
| Soil.origin:Plant.condition:Microcompartment | 2 | 36 | 2.45032 | 0.10053179 |  |
| Signif. codes: 0 ‘***’ 0.001 ‘**’ 0.01 ‘*’ 0.05 ‘.’ 0.1 ‘ ’ 1 |  |  |  |  |  |
