# Supplementary material for: Chaetomium, Chlonostachys, and Pseudogymnoascus isolates from tomato tissues significantly suppress Phytophthora infestans in tomato
Source: PLoS One. 2025 Oct 24;20(10):e0335007. doi: 10.1371/journal.pone.0335007 (PMC12551835; doi:10.1371/journal.pone.0335007)
Supplement: S4 Table — (DOCX) [file pone.0335007.s004.docx]

*Chaetomium*, *Chlonostachys,* and *Pseudogymnoascus* isolates from tomato tissues significantly suppress *Phytophthora  infestans* in tomato

Philemon Orwa^1^, Theresa Kuhl-Nagel^2^, Rosa Meinhold-Ernst^1^, Arne Seyer^1,4^, Johannes A. Jehle^1^, Romano Mwirichia^3^, Ada Linkies^1*^

^1^ Julius Kühn Institute (JKI) - Federal Research Centre for Cultivated Plants, Institute for Biological Control, 69221 Dossenheim, Germany

^2^ Leibniz Institute of Vegetable and Ornamental Crops (IGZ), Plant-Microbe Systems, Großbeeren, Germany

^3^University of Embu, Department of Biological Sciences, 6-60100 Embu, Kenya

^4^Geisenheim University, Department of Crop Protection, 65366 Geisenheim, Germany

* Corresponding author

ada.linkies@julius-kuehn.de

| Analysis of Variance of Aligned Rank Transformed Data  Table Type: Anova Table (Type III tests)  Model: No Repeated Measures (lm)  Response: art(Simpson) | | | | | |
| --- | --- | --- | --- | --- | --- |
|  | Df | Df.res | F value | Pr(>F) | Signif. level |
| Soil.origin | 1 | 36 | 0.16143 | 0.690219 |  |
| Plant.condition | 1 | 36 | 4.06290 | 0.051347 |  |
| Microcompartment | 2 | 36 | 29.23324 | 2.8737e-08 | *** 0 |
| Soil.origin:Plant.condition | 1 | 36 | 1.48119 | 0.231507 |  |
| Soil.origin:Microcompartment | 2 | 36 | 2.03093 | 0.145978 |  |
| Plant.condition:Microcompartment | 2 | 36 | 3.12250 | 0.056168 |  |
| Soil.origin:Plant.condition:Microcompartment | 2 | 36 | 0.98839 | 0.382047 |  |
| Signif. codes: 0 ‘***’ 0.001 ‘**’ 0.01 ‘*’ 0.05 ‘.’ 0.1 ‘ ’ 1 |  |  |  |  |  |

**S4 Table.** **Non-parametric aligned-rank ANOVA results for the Simpson index using the ARTool package (v.0.11.1)**. The effects of plant condition, microcompartment, and soil origin, and their interactions on the Simpson index are shown. Significant effects are shown at p < 0.05.
