# Supplementary material for: Chaetomium, Chlonostachys, and Pseudogymnoascus isolates from tomato tissues significantly suppress Phytophthora infestans in tomato
Source: PLoS One. 2025 Oct 24;20(10):e0335007. doi: 10.1371/journal.pone.0335007 (PMC12551835; doi:10.1371/journal.pone.0335007)
Supplement: S5 Table — (DOCX) [file pone.0335007.s005.docx]

*Chaetomium*, *Chlonostachys,* and *Pseudogymnoascus* isolates from tomato tissues significantly suppress *Phytophthora  infestans* in tomato

Philemon Orwa^1^, Theresa Kuhl-Nagel^2^, Rosa Meinhold-Ernst^1^, Arne Seyer^1,4^, Johannes A. Jehle^1^, Romano Mwirichia^3^, Ada Linkies^1*^

^1^ Julius Kühn Institute (JKI) - Federal Research Centre for Cultivated Plants, Institute for Biological Control, 69221 Dossenheim, Germany

^2^ Leibniz Institute of Vegetable and Ornamental Crops (IGZ), Plant-Microbe Systems, Großbeeren, Germany

^3^University of Embu, Department of Biological Sciences, 6-60100 Embu, Kenya

^4^Geisenheim University, Department of Crop Protection, 65366 Geisenheim, Germany

* Corresponding author

ada.linkies@julius-kuehn.de

| Permutation test for adonis under reduced model  Terms added sequentially (first to last)  Permutation: free  Number of permutations: 9999  adonis2(formula = ASVs_rela_dist ~ Plant.condition * Microcompartment * Soil.origin, data = metadat, permutations = 9999, method = "Bray") | | | | | |
| --- | --- | --- | --- | --- | --- |
|  | Df | Sumof  Sqs | F | Pr(>F) | Signif. level |
| Plant.condition | 1 | 0.1974 | 1.4181 | 0.1763 |  |
| Microcompartment | 2 | 6.5498 | 23.5249 | 0.0001 | *** 0 |
| Soil.origin | 1 | 2.1536 | 15.4700 | 0.0001 | *** 0 |
| Plant.condition:Microcompartment | 2 | 0.4827 | 1.7337 | 0.0380 | * 0.01 |
| Plant.condition:Soil.origin | 1 | 0.1699 | 1.2207 | 0.2650 |  |
| Microcompartment:Soil.origin | 2 | 2.3930 | 8.5949 | 0.0001 | *** 0 |
| Plant.condition:Microcompartment:Soil.origin | 2 | 0.3664 | 0.02115 | 0.1758 |  |
| Signif. codes: 0 ‘***’ 0.001 ‘**’ 0.01 ‘*’ 0.05 ‘.’ 0.1 ‘ ’ 1 |  |  |  |  |  |

**S5 Table.** **Permutation test results from Adonis2 for beta diversity analysis with Bray-Curtis dissimilarity.** The model assesses the effect of plant condition, microcompartment, and soil origin, as well as their interactions, on fungal community composition. Significant effects are shown at p < 0.05 based on PERMANOVA (Permutational Multivariate Analysis of Variance).
