# Supplementary material for: Chaetomium, Chlonostachys, and Pseudogymnoascus isolates from tomato tissues significantly suppress Phytophthora infestans in tomato
Source: PLoS One. 2025 Oct 24;20(10):e0335007. doi: 10.1371/journal.pone.0335007 (PMC12551835; doi:10.1371/journal.pone.0335007)
Supplement: S8 Table — (DOCX) [file pone.0335007.s008.docx]

*Chaetomium*, *Chlonostachys,* and *Pseudogymnoascus* isolates from tomato tissues significantly suppress *Phytophthora  infestans* in tomato

Philemon Orwa^1^, Theresa Kuhl-Nagel^2^, Rosa Meinhold-Ernst^1^, Arne Seyer^1,4^, Johannes A. Jehle^1^, Romano Mwirichia^3^, Ada Linkies^1*^

^1^ Julius Kühn Institute (JKI) - Federal Research Centre for Cultivated Plants, Institute for Biological Control, 69221 Dossenheim, Germany

^2^ Leibniz Institute of Vegetable and Ornamental Crops (IGZ), Plant-Microbe Systems, Großbeeren, Germany

^3^University of Embu, Department of Biological Sciences, 6-60100 Embu, Kenya

^4^Geisenheim University, Department of Crop Protection, 65366 Geisenheim, Germany

* Corresponding author

ada.linkies@julius-kuehn.de

**S8 Table. ANCOM-BC2 results of differential abundance of fungal ASVs between soil A and soil B in endosphere samples based on Log Fold Change (LogFC) and structural zero analysis.** The table shows only ASVs that matched with candidate fungal antagonists. Negative LogFC values indicate higher abundance in soil A, while positive values indicate higher abundance in soil B. ASVs classified as structural zero are exclusively present in one soil type and are represented by strongly negative LFC and strongly positive LFC.

| **ASV id from the microbiome dataset** | **Taxonomy (BLAST) genus match of the isolates** | **Isolate(s) code** | **LogFC Soil (B-A) or structural zero** | **Higher abundance in** |
| --- | --- | --- | --- | --- |
| 14 | *Cladosporium* sp. | Pf179, Pf242 | 0.561437844 | Soil B |
| 22 | *Penicillium* sp. | Pf15 | 0398047123 | Soil B |
| 23 | *Verticillium* sp. | Pf226 | 0.148012815 | Soil B |
| 29 | *Mortierella* sp. | Pf194 | -0-09408423 | Soil A |
| 29 | *Chaetomium* sp. | Pf101 | Structural zero | Exclusively in soil B |
| 33 | *Clonostachys* sp. | Pf4 | Structural zero | Exclusively in soil A |
| 47 | *Mortierella* sp. | Pf210 | 0.509529953 | Soil B |
| 88 | *Penicillium* sp. | Pf52 | Structural zero | Exclusively in soil B |
| 126 | *Ctenomyces* sp. | Pf45 | Structural zero | Exclusively in soil A |
| 133 | *Clonostachys* sp*.* | Pf22 | 0.738995899 | Soil B |
| 174 | *Trichoderma* sp. | Pf32 | -2.723150229 | Soil A |
| 178 | *Trichoderma* sp. | Pf205 | Structural zero | Exclusively in soil B |
| 1009 | *Penicillium* sp*.* | Pf15 | Structural zero | Exclusively in soil A |
| 2224 | *Ctenomyces* sp. | Pf45 | Structural zero | Exclusively in soil A |
