# Supplementary material for: Chaetomium, Chlonostachys, and Pseudogymnoascus isolates from tomato tissues significantly suppress Phytophthora infestans in tomato
Source: PLoS One. 2025 Oct 24;20(10):e0335007. doi: 10.1371/journal.pone.0335007 (PMC12551835; doi:10.1371/journal.pone.0335007)
Supplement: S9 Table — (DOCX) [file pone.0335007.s009.docx]

*Chaetomium*, *Chlonostachys,* and *Pseudogymnoascus* isolates from tomato tissues significantly suppress *Phytophthora  infestans* in tomato

Philemon Orwa^1^, Theresa Kuhl-Nagel^2^, Rosa Meinhold-Ernst^1^, Arne Seyer^1,4^, Johannes A. Jehle^1^, Romano Mwirichia^3^, Ada Linkies^1*^

^1^ Julius Kühn Institute (JKI) - Federal Research Centre for Cultivated Plants, Institute for Biological Control, 69221 Dossenheim, Germany

^2^ Leibniz Institute of Vegetable and Ornamental Crops (IGZ), Plant-Microbe Systems, Großbeeren, Germany

^3^University of Embu, Department of Biological Sciences, 6-60100 Embu, Kenya

^4^Geisenheim University, Department of Crop Protection, 65366 Geisenheim, Germany

* Corresponding author

ada.linkies@julius-kuehn.de

**S9 Table: Total collection of fungal isolates inhibiting *P. infestans, in vitro.*** The *in vitro* tests were conducetd on PDA*.* Data on isolates inhibiting *A. solani*, *in vitro* are included. Isolation parameters include media and temperature used for the isolation of fungi from plant samples. The putative genus matches were determined by BLAST analysis after sequencing the ITS locus. PDA: Potato Dextrose Agar; OMA: Oat Meal Agar

|  | | | | Isolation parameters | | NCBI BLAST close relative (ITS) | | *In vitro* inhibitory activity on PDA | |
| --- | --- | --- | --- | --- | --- | --- | --- | --- | --- |
| **S/N** | **Isolate code** | **Sample source** | **Soil origin** | **Isolation Temperature (°C)** | **Isolation Media** | **Probable genus match with (%) identity** | **Accesion number** | ***vs P. infestans*** | ***vs A. solani*** |
| 1 | Pf 19 | Healthy phyllosphere | B | 14 | OMA | *Acrostalagmus sp.  99%* | PX060186 | yes | no |
| 2 | Pf 78 | Diseased rhizosphere | A | 14 | OMA | *Acrostalagmus sp. 100%* | PX060202 | yes | no |
| 3 | Pf 202 | Healthy rhizosphere | B | 14 | OMA | *Acrostalagmus sp. 100%* | PX060230 | yes | no |
| 4 | Pf 230 | Healthy rhizosphere | B | 14 | OMA | *Acrostalagmus sp. 100%* | PX060238 | yes | no |
| 5 | Pf 121 | Diseased rhizosphere | A | 28 | OMA | *Bionectria sp. 100%* | PX060213 | yes | no |
| 6 | Pf 154 | Healthy rhizosphere | A | 28 | PDA | *Bionectria sp. 100%* | PX060220 | yes | no |
| 7 | Pf 101 | Healthy rhizosphere | B | 21 | PDA | *Chaetomium sp. 100%* | PX060209 | yes | no |
| 8 | Pf 179 | Diseased phyllosphere | A | 28 | PDA | *Cladosporium sp. 100%* | PX060226 | yes | no |
| 9 | Pf 242 | Diseased phyllosphere | B | 21 | OMA | *Cladosporium sp. 100%* | PX060240 | yes | no |
| 10 | Pf 4 | Healthy rhizosphere | A | 28 | OMA | *Clonostachys sp. 100%* | PX060182 | yes | no |
| 11 | Pf 22 | Diseased rhizosphere | A | 14 | PDA | *Clonostachys sp. 100%* | PX060187 | yes | no |
| 12 | Pf 27 | Healthy rhizosphere | A | 14 | OMA | *Clonostachys sp. 100%* | PX060190 | yes | no |
| 13 | Pf 45 | Diseased rhizosphere | A | 21 | PDA | *Ctenomyces sp. 100%* | PX060194 | yes | no |
| 14 | Pf 56 | Healthy rhizosphere | A | 14 | OMA | *Furcasterigmium sp. 100%* | PX060197 | yes | no |
| 15 | Pf 213 | Diseased rhizosphere | B | 28 | PDA | *Fusarium sp. 100%* | PX060233 | yes | yes |
| 16 | Pf 158 | Diseased rhizosphere | A | 14 | OMA | *Geomyces sp. 99.79%* | PX060222 | yes | no |
| 17 | Pf 75 | Diseased rhizosphere | B | 14 | OMA | *Humicola sp. 100%* | PX060201 | yes | no |
| 18 | Pf 155 | Healthy rhizosphere | A | 28 | PDA | *Mortierella sp. 99.82%* | PX060221 | yes | yes |
| 19 | Pf 194 | Healthy rhizosphere | A | 14 | PDA | *Mortierella sp. 98.82%* | PX060229 | yes | no |
| 20 | Pf 210 | Healthy rhizosphere | A | 28 | OMA | *Mortierella sp. 99.44%* | PX060232 | yes | yes |
| 21 | Pf 86 | Diseased rhizosphere | A | 14 | OMA | *Myrothecium sp. 100%* | PX060205 | yes | no |
| 22 | Pf 31 | Healthy rhizosphere | A | 21 | PDA | *Paraphaeosphaeria sp. 99.82%* | PX060191 | yes | no |
| 23 | Pf2 | Diseased rhizosphere | A | 28 | OMA | *Penicillium sp. 100%* | PX060181 | yes | no |
| 24 | Pf 12 | Healthy phyllosphere | A | 14 | PDA | *Penicillium sp. 99.63%* | PX060183 | yes | yes |
| 25 | Pf 13 | Diseased phyllosphere | B | 14 | PDA | *Penicillium sp. 100%* | PX060184 | yes | yes |
| 26 | Pf 15 | Healthy phyllosphere | A | 21 | PDA | *Penicillium sp. 100%* | PX060185 | yes | no |
| 27 | Pf 26 | Healthy rhizosphere | A | 21 | PDA | *Penicillium sp. 100%* | PX060189 | yes | yes |
| 28 | Pf 35 | Healthy rhizosphere | B | 28 | PDA | *Penicillium sp. 99.82%* | PX060193 | yes | yes |
| 29 | Pf 52 | Healthy rhizosphere | B | 14 | PDA | *Penicillium sp. 100%* | PX060195 | yes | yes |
| 30 | Pf 54 | Healthy rhizosphere | A | 28 | PDA | *Penicillium sp. 100%* | PX060196 | yes | yes |
| 31 | Pf 61 | Healthy phyllosphere | A | 28 | PDA | *Penicillium sp. 100%* | PX060198 | yes | yes |
| 32 | Pf 70 | Diseased rhizosphere | B | 14 | PDA | *Penicillium sp. 100%* | PX060199 | yes | no |
| 33 | Pf 73 | Diseased rhizosphere | B | 28 | PDA | *Penicillium sp. 100%* | PX060200 | yes | no |
| 34 | Pf 83 | Diseased phyllosphere | A | 14 | PDA | *Penicillium sp. 100%* | PX060203 | yes | no |
| 35 | Pf 85 | Diseased rhizosphere | A | 28 | OMA | *Penicillium sp. 100%* | PX060204 | yes | no |
| 36 | Pf 88 | Healthy phyllosphere | A | 14 | PDA | *Penicillium sp.  99.82%* | PX060206 | yes | yes |
| 37 | Pf 98 | Healthy rhizosphere | B | 28 | PDA | *Penicillium sp. 100%* | PX060208 | yes | yes |
| 38 | Pf 104 | Healthy rhizosphere | B | 21 | PDA | *Penicillium sp. 100%* | PX060210 | yes | yes |
| 39 | Pf 109 | Healthy rhizosphere | B | 28 | OMA | *Penicillium sp. 100%* | PX060211 | yes | yes |
| 40 | Pf 124 | Healthy rhizosphere | B | 21 | PDA | *Penicillium sp. 100%* | PX060214 | yes | yes |
| 41 | Pf 125 | Diseased phyllosphere | A | 14 | OMA | *Penicillium sp.  99.82%* | PX060215 | yes | no |
| 42 | Pf 138 | Diseased phyllosphere | B | 14 | OMA | *Penicillium sp. 100%* | PX060218 | yes | yes |
| 43 | Pf 148 | Diseased rhizosphere | A | 28 | PDA | *Penicillium sp. 99.83%* | PX060219 | yes | yes |
| 44 | Pf 161 | Diseased phyllosphere | B | 14 | PDA | *Penicillium sp. 100%* | PX060223 | yes | no |
| 45 | Pf 162 | Healthy rhizosphere | A | 14 | OMA | *Penicillium sp. 99.47%* | PX060224 | yes | no |
| 46 | Pf 185 | Diseased phyllosphere | B | 28 | OMA | *Penicillium sp. 100%* | PX060227 | yes | yes |
| 47 | Pf 193 | Diseased rhizosphere | A | 21 | OMA | *Penicillium sp. 99%* | PX060228 | yes | yes |
| 48 | Pf 232 | Diseased phyllosphere | B | 28 | PDA | *Penicillium sp. 100%* | PX060239 | yes | no |
| 49 | Pf 246 | Diseased phyllosphere | A | 14 | OMA | *Penicillium sp. 100%* | PX060241 | yes | no |
| 50 | Pf 23 | Diseased rhizosphere | A | 21 | OMA | *Pseudogymnoascus sp. 100%* | PX060188 | yes | yes |
| 51 | Pf 117 | Healthy rhizosphere | A | 14 | PDA | *Pseudogymnoascus sp. 100%* | PX060212 | yes | no |
| 52 | Pf 97 | Diseased rhizosphere | B | 28 | PDA | *Scytalidium sp. 99.64%* | PX060207 | yes | yes |
| 53 | Pf 133 | Diseased rhizosphere | B | 14 | PDA | *Talaromyces sp. 100%* | PX060217 | yes | no |
| 54 | Pf 165 | Diseased rhizosphere | A | 28 | OMA | *Trametes sp. 99.83%* | PX060225 | yes | yes |
| 55 | Pf 32 | Healthy rhizosphere | B | 28 | OMA | *Trichoderma sp. 100%* | PX060192 | yes | no |
| 56 | Pf 131 | Diseased phyllosphere | B | 14 | PDA | *Trichoderma sp. 100%* | PX060216 | yes | yes |
| 57 | Pf 205 | Healthy rhizosphere | B | 28 | PDA | *Trichoderma sp. 100%* | PX060231 | yes | yes |
| 58 | Pf 218 | Diseased rhizosphere | B | 21 | OMA | *Trichoderma sp. 100%* | PX060218 | yes | yes |
| 59 | Pf 223 | Healthy rhizosphere | B | 14 | OMA | *Trichoderma sp. 100%* | PX060235 | yes | yes |
| 60 | Pf 225 | Diseased rhizosphere | B | 14 | OMA | *Trichoderma sp. 100%* | PX060236 | yes | yes |
| 61 | Pf 226 | Diseased rhizosphere | B | 28 | OMA | *Verticillium sp. 100%* | PX060237 | yes | no |
