# Supplementary material for: Chaetomium, Chlonostachys, and Pseudogymnoascus isolates from tomato tissues significantly suppress Phytophthora infestans in tomato
Source: PLoS One. 2025 Oct 24;20(10):e0335007. doi: 10.1371/journal.pone.0335007 (PMC12551835; doi:10.1371/journal.pone.0335007)
Supplement: S10 Table — (DOCX) [file pone.0335007.s010.docx]

*Chaetomium*, *Chlonostachys,* and *Pseudogymnoascus* isolates from tomato tissues significantly suppress *Phytophthora  infestans* in tomato

Philemon Orwa^1^, Theresa Kuhl-Nagel^2^, Rosa Meinhold-Ernst^1^, Arne Seyer^1,4^, Johannes A. Jehle^1^, Romano Mwirichia^3^, Ada Linkies^1*^

^1^ Julius Kühn Institute (JKI) - Federal Research Centre for Cultivated Plants, Institute for Biological Control, 69221 Dossenheim, Germany

^2^ Leibniz Institute of Vegetable and Ornamental Crops (IGZ), Plant-Microbe Systems, Großbeeren, Germany

^3^University of Embu, Department of Biological Sciences, 6-60100 Embu, Kenya

^4^Geisenheim University, Department of Crop Protection, 65366 Geisenheim, Germany

* Corresponding author

ada.linkies@julius-kuehn.de

**S10 Table. Collection of fungal biocontrol candidates inhibiting *P. infestans* after eliminating potential plant and human pathogens based on literature search.** The *in vitro* tests were conducetd on rye agar*.* The putative genera were determined by BLAST analysis after partial sequencing of the ITS locus (approximately 560bp). Isolation parameters include media and temperature used for the isolation of fungi from plant samples. PDA: potato dextrose agar; OMA: oatmeal agar

|  | NCBI BLAST close relative (ITS loci) | |  | | Isolation parameters | | *In vitro* inhibitory activity on rye agar |
| --- | --- | --- | --- | --- | --- | --- | --- |
| **Isolate code** | **Probable genus match with (%) identity** | **Accession number** | **Sample source** | **Soil origin** | **Isolation temperature (°C)** | **Isolation Media** | ***vs P. infestans*** |
| Pf121 | *Bionectria* sp*.* | PX060213 | Diseased rhizosphere | A | 28 | OMA | yes |
| Pf 154 | *Bionectria* sp*.* | PX060220 | Healthy rhizosphere | A | 28 | PDA | yes |
| Pf101 | *Chaetomium* sp*.* | PX060209 | Healthy rhizosphere | B | 21 | PDA | yes |
| Pf 179 | *Cladosporium* sp*.* | PX060226 | Diseased leaves | A | 28 | PDA | yes |
| Pf 242 | *Cladosporium* sp*.* | PX060240 | Diseased phyllosphere | B | 21 | OMA | yes |
| Pf 4 | *Clonostachys* sp*.* | PX060182 | Healthy rhizosphere | A | 28 | OMA | yes |
| Pf 22 | *Clonostachys* sp*.* | PX060187 | Diseased rhizosphere | A | 14 | PDA | yes |
| Pf 27 | *Clonostachys* sp*.* | PX060190 | Healthy rhizosphere | A | 14 | OMA | yes |
| Pf 45 | *Ctenomyces* sp*.* | PX060194 | Diseased rhizosphere | A | 21 | PDA | yes |
| Pf 56 | *Furcasterigmium* sp*.* | PX060197 | Healthy rhizosphere | A | 14 | OMA | yes |
| Pf 158 | *Geomyces* sp*.* | PX060222 | Diseased rhizosphere | A | 14 | OMA | yes |
| Pf 155 | *Mortierella* sp*.* | PX060221 | Healthy rhizosphere | A | 28 | PDA | yes |
| Pf 194 | *Mortierella* sp*.* | PX060229 | Healthy rhizosphere | A | 14 | PDA | yes |
| Pf 210 | *Mortierella* sp*.* | PX060232 | Healthy rhizosphere | A | 28 | OMA | yes |
| Pf 86 | *Myrothecium* sp*.* | PX060205 | Diseased rhizosphere | A | 14 | OMA | yes |
| Pf 31 | *Paraphaeosphaeria* sp*.* | PX060191 | Healthy rhizosphere | A | 21 | PDA | yes |
| Pf 23 | *Pseudogymnoascus* sp*.* | PX060188 | Diseased rhizosphere | A | 21 | OMA | yes |
| Pf 117 | *Pseudogymnoascus* sp*.* | PX060212 | Healthy rhizosphere | A | 14 | PDA | yes |
| Pf 133 | *Talaromyces* sp*.* | PX060217 | Diseased rhizosphere | B | 14 | PDA | yes |
| Pf 165 | *Trametes* sp*.* | PX060225 | Diseased rhizosphere | A | 28 | OMA | yes |
| Pf 131 | *Trichoderma* sp*.* | PX060216 | Diseased leaves | B | 14 | PDA | yes |
| Pf 205 | *Trichoderma* sp*.* | PX060231 | Healthy rhizosphere | B | 28 | PDA | yes |
| Pf 218 | *Trichoderma* sp*.* | PX060234 | Diseased rhizosphere | B | 21 | OMA | yes |
| Pf 223 | *Trichoderma* sp*.* | PX060235 | Healthy rhizosphere | B | 14 | OMA | yes |
