# Supplementary material for: Chaetomium, Chlonostachys, and Pseudogymnoascus isolates from tomato tissues significantly suppress Phytophthora infestans in tomato
Source: PLoS One. 2025 Oct 24;20(10):e0335007. doi: 10.1371/journal.pone.0335007 (PMC12551835; doi:10.1371/journal.pone.0335007)
Supplement: S1 Fig — (DOCX) [file pone.0335007.s013.docx]

*Chaetomium*, *Chlonostachys,* and *Pseudogymnoascus* isolates from tomato tissues significantly suppress *Phytophthora  infestans* in tomato

Philemon Orwa^1^, Theresa Kuhl-Nagel^2^, Rosa Meinhold-Ernst^1^, Arne Seyer^1,4^, Johannes A. Jehle^1^, Romano Mwirichia^3^, Ada Linkies^1*^

^1^ Julius Kühn Institute (JKI) - Federal Research Centre for Cultivated Plants, Institute for Biological Control, 69221 Dossenheim, Germany

^2^ Leibniz Institute of Vegetable and Ornamental Crops (IGZ), Plant-Microbe Systems, Großbeeren, Germany

^3^University of Embu, Department of Biological Sciences, 6-60100 Embu, Kenya

^4^Geisenheim University, Department of Crop Protection, 65366 Geisenheim, Germany

* Corresponding author

ada.linkies@julius-kuehn.de


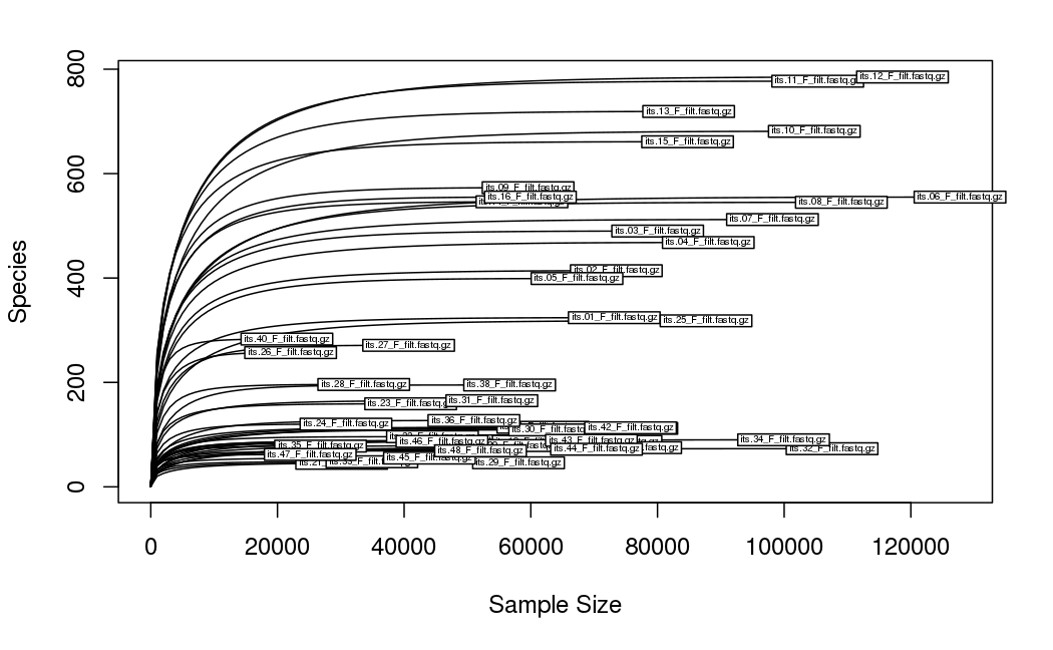


**S1 Fig.** **Rarefaction curve of observed species richness after sequencing of ITS rRNA gene from the rhizosphere, endosphere, and phyllosphere samples of tomato plants grown in soil origins A and B.** The curve was generated by subsampling the dataset multiple times (1000 random rarefaction runs) to estimate ASV distribution at different sequencing depths. All curves reaching saturation indicate sufficient sequencing depth to cover microbial diversity.
