# Supplementary material for: Chaetomium, Chlonostachys, and Pseudogymnoascus isolates from tomato tissues significantly suppress Phytophthora infestans in tomato
Source: PLoS One. 2025 Oct 24;20(10):e0335007. doi: 10.1371/journal.pone.0335007 (PMC12551835; doi:10.1371/journal.pone.0335007)
Supplement: Attachment — Submitted filename: PONE-D-25-31666_R1.pdf [file pone.0335007.s016.pdf]

PLOS ONE

<i>Chaetomium</i>, <i>Chlonostachys</i>, and <i>Pseudogymnoascus</i> isolates from tomato tissues significantly suppress <i>Phytophthora infestans</i> in tomato

--Manuscript Draft--

**Manuscript Number:**	PONE-D-25-31666R1
**Article Type:**	Research Article
**Full Title:**	<i>Chaetomium</i>, <i>Chlonostachys</i>, and <i>Pseudogymnoascus</i> isolates from tomato tissues significantly suppress <i>Phytophthora infestans</i> in tomato
**Short Title:**	Fungal isolates significantly suppress <i>P. infestans</i> in tomato
**Corresponding Author:**	Ada Linkies<br>Julius Kühn Institute: Julius Kuhn-Institut<br>Dossenheim, GERMANY
**Keywords:**	fungal microbiome; ITS rDNA; Illumina sequencing; tomato rhizosphere; microbial antagonists; Biological control; Tomato; Phytophthora infestans; Chaetomium
**Abstract:**	Late blight is a disease whose causative agent is the oomycete Phytophthora infestans. It is one of the most destructive pathogenic oomycetes and a major challenge to global tomato production. The pathogen is difficult to manage because of its ability to evolve thereby evading host resistance. The aim of this study was to screen for potential antagonists of P. infestans using a combination of culture and microbiome-based approaches. Samples were collected from healthy and P. infestans-infected tomato plants grown in soil collected from two organic tomato growers in the Rhine-Main area in Germany. A total of 246 fungal isolates were screened for their antagonistic activity against P. infestans. Most of the isolates that exhibited in vitro antagonistic activity were from the genera Penicillium, Trichoderma, Chlonostachys, Mortierella, and Pseudogymnoascus. Following a stepwise in vitro screening strategy that accounted for growth features, ecological aspects, taxonomic data, potential health risks, commercial properties, and antagonistic efficacy, five fungal isolates were eventually selected for plant trials. Chaetomiumsubaffine showed the highest inhibitory effect against P. infestans across three trials whereby the percentage of diseased leaf area reduced by 90% compared to the control. Chlonostachys and Pseudogymnoascus spp. were effective in two trials, while Trichoderma and Ctenomyces spp. showed weak disease suppressive effects. In parallel, we characterized the fungal microbiome of the rhizosphere, phyllosphere, and endosphere from healthy and diseased tomato plants using ITS-rRNA sequencing. The fungal community differed significantly between the two soil origins, but P. infestans did not significantly influence fungal microbiota composition. Notably, 70% of our antagonistic fungi from the culture collection were detected in the tomato microbiome. This work identified isolates of Chaetomium subaffine, Clonostachys sp., and Pseudogymnoascus sp. as potential biocontrol candidates promoting plant health. The findings highlight the importance of combined functional screening and microbiome profiling for identifying fungal antagonists.
**Order of Authors:**	Philemon Orwa
	Theresa Kuhl-Nagel
	Rosa Meinhold-Ernst
	Arne Seyer
	Johannes Alois Jehle
	Romano Mwirichia
	Ada Linkies
**Response to Reviewers:**	We replied in detail to each comment from both editor and the reviewers in the included `response to reviewers´file.
**Additional Information:**	
**Question**	**Response**

Powered by Editorial Manager® and ProduXion Manager® from Aries Systems Corporation

**Financial Disclosure**<br><br>Enter a financial disclosure statement that describes the sources of funding for the work included in this submission. Review the submission guidelines for detailed requirements. View published research articles from *PLOS ONE* for specific examples.<br><br>This statement is required for submission and **will appear in the published article** if the submission is accepted. Please make sure it is accurate.<br><br>**Funded studies**<br>Enter a statement with the following details:<br>• Initials of the authors who received each award<br>• Grant numbers awarded to each author<br>• The full name of each funder<br>• URL of each funder website<br>• Did the sponsors or funders play any role in the study design, data collection and analysis, decision to publish, or preparation of the manuscript?<br><br>Did you receive funding for this work?	Yes
Please add funding details.<br>as follow-up to "**Financial Disclosure**<br><br>Enter a financial disclosure statement that describes the sources of funding for the work included in this submission. Review the submission guidelines for detailed requirements. View published research articles from *PLOS ONE* for specific examples.<br><br>This statement is required for submission and **will appear in the published article** if the submission is accepted. Please make sure it is accurate.	Deutscher Akademischer Austauschdienst, grant no.

**Funded studies**<br>Enter a statement with the following details:<br>• Initials of the authors who received each award<br>• Grant numbers awarded to each author<br>• The full name of each funder<br>• URL of each funder website<br>• Did the sponsors or funders play any role in the study design, data collection and analysis, decision to publish, or preparation of the manuscript?<br><br>Did you receive funding for this work?"	
Please select the country of your main research funder (please select carefully as in some cases this is used in fee calculation).<br>as follow-up to "**Financial Disclosure**<br><br>Enter a financial disclosure statement that describes the sources of funding for the work included in this submission. Review the submission guidelines for detailed requirements. View published research articles from *PLOS ONE* for specific examples.<br><br>This statement is required for submission and **will appear in the published article** if the submission is accepted. Please make sure it is accurate.<br><br>**Funded studies**<br>Enter a statement with the following details:<br>• Initials of the authors who received each award<br>• Grant numbers awarded to each author<br>• The full name of each funder<br>• URL of each funder website<br>• Did the sponsors or funders play any role in the study design, data collection and analysis, decision to publish, or preparation of the manuscript?<br><br>Did you receive funding for this work?"	GERMANY - DE
**Competing Interests**	The authors have declared that no competing interests exist.

Use the instructions below to enter a competing interest statement for this submission. On behalf of all authors, disclose any competing interests that could be perceived to bias this work—acknowledging all financial support and any other relevant financial or non-financial competing interests.<br><br>This statement is required for submission and **will appear in the published article** if the submission is accepted. Please make sure it is accurate and that any funding sources listed in your Funding Information later in the submission form are also declared in your Financial Disclosure statement.<br><br>View published research articles from *PLOS ONE* for specific examples.<br><br>**NO authors have competing interests**<br><br>Enter: *The authors have declared that no competing interests exist.*<br><br>**Authors with competing interests**<br><br>Enter competing interest details beginning with this statement:<br><br>*I have read the journal's policy and the authors of this manuscript have the following competing interests: [insert competing interests here]*<br><br>* typeset	
**Ethics Statement**<br><br>Enter an ethics statement for this submission. This statement is required if the study involved:<br><br>• Human participants<br>• Human specimens or tissue<br>• Vertebrate animals or cephalopods<br>• Vertebrate embryos or tissues	N/A

Powered by Editorial Manager® and ProduXion Manager® from Aries Systems Corporation

• Field research<br><br>Write "N/A" if the submission does not require an ethics statement.<br><br>General guidance is provided below. Consult the submission guidelines for detailed instructions. **Make sure that all information entered here is included in the Methods section of the manuscript.**	

Powered by Editorial Manager® and ProduXion Manager® from Aries Systems Corporation

**Format for specific study types**<br><br>**Human Subject Research (involving human participants and/or tissue)**<br>• Give the name of the institutional review board or ethics committee that approved the study<br>• Include the approval number and/or a statement indicating approval of this research<br>• Indicate the form of consent obtained (written/oral) or the reason that consent was not obtained (e.g. the data were analyzed anonymously)<br><br>**Animal Research (involving vertebrate animals, embryos or tissues)**<br>• Provide the name of the Institutional Animal Care and Use Committee (IACUC) or other relevant ethics board that reviewed the study protocol, and indicate whether they approved this research or granted a formal waiver of ethical approval<br>• Include an approval number if one was obtained<br>• If the study involved *non-human primates*, add *additional details* about animal welfare and steps taken to ameliorate suffering<br>• If anesthesia, euthanasia, or any kind of animal sacrifice is part of the study, include briefly which substances and/or methods were applied<br><br>**Field Research**<br><br>Include the following details if this study involves the collection of plant, animal, or other materials from a natural setting:<br>• Field permit number<br>• Name of the institution or relevant body that granted permission	
**Data Availability**<br><br>Authors are required to make all data underlying the findings described fully available, without restriction, and from the time of publication. PLOS allows rare exceptions to address legal and ethical concerns. See the PLOS Data Policy and FAQ for detailed information.	Yes - all data are fully available without restriction

A Data Availability Statement describing where the data can be found is required at submission. Your answers to this question constitute the Data Availability Statement and **will be published in the article**, if accepted.<br><br>**Important:** Stating 'data available on request from the author' is not sufficient. If your data are only available upon request, select 'No' for the first question and explain your exceptional situation in the text box.<br><br>Do the authors confirm that all data underlying the findings described in their manuscript are fully available without restriction?	
**Describe where the data may be found in full sentences. If you are copying our sample text, replace any instances of XXX with the appropriate details.**<br><br>• If the data are **held or will be held in a public repository**, include URLs, accession numbers or DOIs. If this information will only be available after acceptance, indicate this by ticking the box below. For example: *All XXX files are available from the XXX database (accession number(s) XXX, XXX.*).<br>• If the data are all contained **within the manuscript and/or Supporting Information files**, enter the following: *All relevant data are within the manuscript and its Supporting Information files.*<br>• If neither of these applies but you are able to provide **details of access elsewhere**, with or without limitations, please do so. For example:<br><br>*Data cannot be shared publicly because of [XXX]. Data are available from the XXX Institutional Data Access / Ethics Committee (contact via XXX) for researchers who meet the criteria for access to confidential data.*<br><br>*The data underlying the results presented in the study are available from (include the name of the third party*	Raw reads are deposited at the Sequence Read Archive (https://www.ncbi.nlm.nih.gov/sra) under the BioProject accession number PRJNA1273108.<br>All scripts including detailed parameters and data to reproduce the figures for this study have been deposited in GitHub (https://github.com/TKuhl-Nagel/Phytophthora_challenged_tomato_microbiome.git).

and contact information or URL).<br>• This text is appropriate if the data are owned by a third party and authors do not have permission to share the data.<br><br>* typeset	
Additional data availability information:	

Powered by Editorial Manager® and ProduXion Manager® from Aries Systems Corporation

Manuscript with track changes accepted
Click here to access/download;Manuscript;Orwa et al_2025 changes accepted.docx

Chaetomium, *Chlonostachys*, and *Pseudogymnoascus* isolates from tomato tissues significantly suppress *Phytophthora infestans* in tomato

Philemon Orwa[1], Theresa Kuhl-Nagel[2], Rosa Meinhold-Ernst[1], Arne Seyer[1,4], Johannes A. Jehle[1], Romano Mwirichia[3], Ada Linkies[1*]

[1] Julius Kühn Institute (JKI) - Federal Research Centre for Cultivated Plants, Institute for Biological Control, Dossenheim, Germany

[2] Leibniz Institute of Vegetable and Ornamental Crops (IGZ), Plant-Microbe Systems, Großbeeren, Germany

[3] University of Embu, Department of Biological Sciences, Embu, Kenya

[4] Geisenheim University, Department of Crop Protection, Geisenheim, Germany

* Corresponding author:

ada.linkies@julius-kuehn.de

Short title: Fungal isolates significantly suppress *P. infestans* in tomato

Abstract

Late blight is a disease whose causative agent is the oomycete *Phytophthora infestans*. It is one of the most destructive pathogenic oomycetes and a major challenge to global tomato production. The pathogen is difficult to manage because of its ability to evolve thereby evading host resistance. The

aim of this study was to screen for potential antagonists of *P. infestans* using a combination of culture and microbiome-based approaches. Samples were collected from healthy and *P. infestans*-infected tomato plants grown in soil collected from two organic tomato growers in the Rhine-Main area in Germany. A total of 246 fungal isolates were screened for their antagonistic activity against *P. infestans*. Most of the isolates that exhibited *in vitro* antagonistic activity were from the genera *Penicillium, Trichoderma, Chlonostachys, Mortierella,* and *Pseudogymnoascus*. Following a stepwise *in vitro* screening strategy that accounted for growth features, ecological aspects, taxonomic data, potential health risks, commercial properties, and antagonistic efficacy, five fungal isolates were eventually selected for plant trials. *Chaetomium subaffine* showed the highest inhibitory effect against *P. infestans* across three trials whereby the percentage of diseased leaf area reduced by 90% compared to the control. *Chlonostachys* and *Pseudogymnoascus* spp. were effective in two trials, while *Trichoderma* and *Ctenomyces* spp. showed weak disease suppressive effects. In parallel, we characterized the fungal microbiome of the rhizosphere, phyllosphere, and endosphere from healthy and diseased tomato plants using ITS-rRNA sequencing. The fungal community differed significantly between the two soil origins, but *P. infestans* did not significantly influence fungal microbiota composition. Notably, 70% of our antagonistic fungi from the culture collection were detected in the tomato microbiome. This work identified isolates of *Chaetomium subaffine, Clonostachys sp.,* and *Pseudogymnoascus sp.* as potential biocontrol candidates promoting plant health. The findings highlight the importance of combined functional screening and microbiome profiling for identifying fungal antagonists.

Keywords: fungal microbiome, ITS rDNA, Illumina sequencing, tomato rhizosphere, microbial antagonists, biological control.

Introduction

Tomato is an important crop forming a vital component of the global diet, owing to its nutritional and health benefits [1]. In addition to other fungal pathogens such as *Alternaria solani*, *Botrytis cinerea*, and *Fusarium oxysporum* f. sp. *lycopersici* [2], late blight disease caused by *Phytophthora infestans*, is among the most devastating pathogenic oomycetes threatening global tomato production [3]. Studies indicate that *P. infestans* has become more aggressive over the past decades [4,5] and that the rapidly evolving genotypes are linked to severe epidemic global outbreaks [6]. Management costs and yield loss associated with late blight disease ranges between 3-10 billion United States Dollars per year globally [7,8].

Conventional approaches for managing *P. infestans* primarily utilize synthetic fungicides, which adversely impact the environment and may harm terrestrial and aquatic organisms [9]. The pathogen also poses the risk of fungicide resistance as it evolves rapidly [10]. In this regard, there are increasingly stringent regulations regarding the use of chemical-synthetic and copper fungicides. The European Union (EU) for instance, has heightened restrictions on the registration of chemical-synthetic pesticides through directives 2009/128/EC and (EU) 2019/782, aiming at lowering the risks associated with pesticide application on humans and the environment [11]. Therefore, there is an urgent need for more eco-friendly alternatives to chemical-synthetic and copper fungicides. Microbial antagonists promote plant growth and suppress plant pathogens through distinct mechanisms, including induced systemic resistance (ISR) [12], competition for space and nutrients [13], or the production of antimicrobial secondary metabolites [14]. The genera *Bacillus* and *Pseudomonas* are commonly found as microbial antagonists [15,16], while *Trichoderma* is among the most investigated and described fungal genera used as a biocontrol agent [17,18].

The soil microbiome includes many beneficial microorganisms and is an important source for plants to recruit their rhizosphere microbiome [19]. Soil microbiomes provide unique properties for plant

pathogen control, also known as soil-borne legacy, which is beneficial to plants of the next generation thriving on the same soil [20]. However, plant pathogens can drive dysbiosis of the plant microbiome with consequences for plant health [21]. The rhizosphere, shaped by plant roots, is a complex habitat for diverse microorganisms, such as protozoa, mycorrhizal fungi, nitrogen-fixing bacteria, plant growth-promoting rhizobacteria (PGPR), and mycoparasitic fungi [22–25]. Fungi play multifaceted roles in the soil, including nitrogen fixation, and synthesis of plant growth–promoting hormones, they are natural biological agents against root pathogens and promoters of plant resilience under drought stress [26–28]. Additionally, fungi are key players in stabilizing soil organic matter by breaking down plant residues [29]. In recent years, there has been a growing emphasis on harnessing the diversity of soil fungi as a sustainable method to improve the quality of soil and boost crop productivity —an emerging strategy holding significant promise for the future of agriculture [30]. Several fungal species have been described with biocontrol potential against *P. infesta*ns [31]. Research on new or better biocontrol agents often focuses mainly on *in vitro* confrontation assay results as the basis for the selection of promising candidates, a majority of which do not meet the requirements for commercial use. The current work seeks to address the above-described research limitations by finding answers to the following questions. (i) Are the micro-compartments of tomato plants a suitable source for isolating fungal strains with effective biocontrol potential against *P. infestans*, which also meet selection criteria for later product development and registration? (ii) Does the microbiome structure differ between healthy and *P. infestans*-infected tomato plants grown in two soil origins, and can specific fungal groups be identified as key contributors to disease suppression? Therefore, we applied an integrated approach in the search for fungal antagonists against *P. infestans* by linking culture-dependent characterization to microbiome analyses and assessing the results in the context of tomato plant health. In the culture-dependent approach, we isolated and characterized potential fungal antagonists from diseased and healthy tomato plant samples in a stepwise selection procedure previously described by Köhl et al. [32] to select promising candidates for *ad planta* trials against *P. infestans*. The selection

procedure incorporates commercial aspects, including growth on low-cost growth media, massive spore production, and evaluating potential health risks, in addition to antagonistic efficacy. In the microbiome approach, we characterized and compared the fungal microbiome of the phyllosphere, the endosphere, and the rhizosphere of diseased and healthy tomato plants grown in soils from different origins using high-throughput sequencing (HTS). Linking culture-dependent work to the microbiome provides deeper insights to characterize beneficial fungi for biocontrol of tomato late blight and other tomato diseases.

Methods

Soil sampling, tomato plant growth conditions, and inoculation with *P. infestans*

Tomato plants (*Solanum lycopersicum* \`Red Robin´ cultivar) were grown in two different natural soils that were previously used for organic tomato cultivation in the greenhouse. No chemical-synthetic pesticides and fertilizers were applied previously and during the experiments. As in both soils tomato plants were grown in the previous growing season, these soils are enriched with organisms that are supportive of tomato plant health. The soils were collected from two different professional tomato growers in the Rhine-Main area at Domäne Mechthildshausen, Wiesbaden, 50°02′ N, 8°19′ E (soil A), and Solidarische Landwirtschaft Rüsselsheim, 49°58′ N, 8°25′ E (soil B) in February 2022. The contact to the growers and the corresponding field sites (soil A and B) was provided by Christian Fetzer, employee from the Landesbetrieb Landwirtschaft Hessen, a governmental educational and advisory institution of the State of Hesse, subordinate to the Hessian Ministry of Agriculture and Environment, Viticulture, Forestry, Hunting, and Home Affairs (HMLU). The growers gave permission to collect soil from their greenhouses for this study. Soil samples were taken at five positions throughout the

greenhouse area with a depth of 20 cm and a mixed sample from all samples was used as substrate for the experiments. Eight tomato seeds were sown in soils from both locations (soil A and B) in each pot (size 8 x 8 x 8.5 cm) within 24 h after soil collection. Four seedlings for each treatment and soil origin were transferred to individual pots 14 days after sowing. Plants were grown at a temperature range of 19-22 °C for subsequent inoculation with *P. infestans* (isolate 606 of JKI culture collection) as described in Drenker et al. [33]. Briefly, four 5-weeks-old plants each grown in soil A and soil B were spray inoculated with 7 ml of 2 x 10^4 ml^{-1} zoospores per plant. Four plants grown in each soil were not inoculated and served as healthy controls. Scoring for disease symptoms (percentage symptomatic area) was performed 2 weeks after inoculation with *P. infestans*.

Sample preparation for microbiome analysis and isolation of fungal antagonists

Samples for partial ITS amplicon sequencing and isolation of viable fungi were collected from the above-described tomato plants. Samples from leaves, roots, and rhizosphere soil were collected from the tomato plant. One plant was considered one replicate, in total four replicate plants per treatment were analyzed. Several plant tissues per micro-compartment were pooled as described in detail below. Plant samples were processed for ITS amplicon sequencing and isolation of fungal antagonists following the protocol of Wieland et al. [34], with some modifications, described as follows.

Rhizosphere sample collection

The roots of each plant were shaken carefully to remove non-adhering soil. Five grams of soil adhering to the roots were transferred in sterile 50 mL centrifuge tubes containing 20 mL of sterile saline buffer (0.85% NaCl) and mixed vigorously by vortexing for 2 min. The roots (approximately 5 g), from which the rhizospheric soil had been dislodged were soaked in 20 mL of sterile saline buffer (0.85% NaCl)

and mixed thoroughly by vortexing for 5 min to obtain the rhizoplane. Both solutions were combined to obtain the final rhizosphere sample. A total of 16 rhizosphere samples were obtained for microbiome analysis, four replicates from each soil origin, both from healthy and diseased plants. The remaining rhizosphere material from the four replicate plants was pooled for the isolation of fungal antagonists. A total of four rhizosphere samples were obtained for fungal isolation, one replicate from each soil origin, and from healthy and diseased plants.

Endosphere sample collection

Roots (approximately 5 g) from rhizoplane extraction described above were sterilized with ethanol (75%; 2 min), sodium hypochlorite solution (50%; 2 min), and ethanol (75%; 1 min) and rinsed five times in sterile distilled water. Root samples (100–150 mg) were then homogenized with a sterile pestle and mortar in 20 mL of sterile saline buffer (0.85% NaCl) to obtain the final endosphere sample. In total, 16 endosphere samples were obtained for microbiome analysis, four replicates from each soil origin, and healthy and diseased plants. The remaining endosphere material from the four replicate plants was pooled for the isolation of fungal antagonists. A total of four endosphere samples were obtained for fungal isolation, one replicate from each soil origin, and from healthy and diseased plants. A sterility check was performed by placing a subset of the sterilized roots on PDA at 27 °C for 7 days, and the absence of microbial growth ensured the sterility of the root surfaces.

Phyllosphere sample collection

Samples for the microbiome analysis and isolation of leaf-associated fungi followed the protocol by Kassa Semagn [35]: Fresh leaf samples (100–150 mg) were harvested from each plant by pooling 4 portions obtained from different branches, cut into smaller pieces into the collection tubes and processed immediately. Notably, samples from plants infected with *P. infestans* were collected from

leaf patches with healthy-looking samples adjacent to the diseased area. A total of 16 rhizosphere samples were obtained for microbiome analysis, four replicates from each soil origin, from both healthy and diseased plants. The remaining phyllosphere material from the four replicate plants was pooled for the isolation of fungal antagonists. A total of four phyllosphere samples were obtained for fungal isolation, one replicate from each soil origin, and from healthy and diseased plants.

Collection of fungal isolates

Culturable fungi were isolated from the phyllosphere and rhizosphere of healthy and diseased plants grown in soil A and B. A total of eight samples were collected for fungal isolation, including four rhizosphere samples (as described above) and four phyllosphere samples as described above. Precisely, 100 µl of serially diluted samples (10^{-1} - 10^{-3}) in sterile saline buffer (0.85% NaCl) were spread-plated onto potato dextrose agar (PDA) and oatmeal agar (OMA) supplemented with antibiotics (rifampicin (20 mg/ml) and streptomycin (50 mg/ml)). Plates were incubated at different temperatures (14, 21, and 28 °C) to increase the diversity of the collection. The first assessment of fungal growth was conducted 7 days after plating and was based on differences in growth morphology, whereas the last assessment of fungal growth was conducted 14 days after plating. Fungal colonies that emerged on plates with different media, at varying temperatures, and from different tissue samples were isolated through a series of sub-culturing steps.

Characterization of fungal isolates regarding their biocontrol potential

The present work adopted a stepwise screening approach developed by Köhl et al. [32] to select fungal candidates with promising characteristics as microbial antagonists of tomato pathogens (Fig 1). The isolated 246 fungi were examined under the microscope for their ability to form spores as this is a key parameter in the mass production of fungi for commercial purposes (S12 Table). The fungal isolates

were further incubated at 37 °C and those that grew in this temperature were eliminated as they could present potential risks to majorly humans and other mammals (S12 Table). Additionally, morphologically identical isolates that appeared repeatedly and those that lost viability were eliminated. The remaining 165 fungal isolates were subjected to dual-culture assays with *P. infestans* as the target plant pathogen in this work (S11 Table). A total of 61 fungal isolates that either formed inhibition zones when co-cultured with the pathogen on Petri dish or overgrew the pathogen were deemed to exhibit antagonistic effects, through antibiosis and matrix completion, respectively (S9 Table). The fungal antagonists were identified as potential biocontrol candidates and were molecularly characterized by sequencing the ITS locus, yielding an amplicon of approximately 560bp (S9 Table). The already identified antagonists were further screened against *A. solani* (isolate 62028 of JKI culture collection) to reveal their broader antagonistic effects (S9 Table). Notably, the *in vitro* confrontation assays with *A. solani* only served as criteria for selecting the most promising antagonists, therefore, the pathogen was not used in the subsequent experiments. A literature search was performed and species that have previously been described as plant, human, or animal pathogens were eliminated, particularly *Penicillium* species which formed a higher proportion of our collection. The resultant collection of 24 fungal isolates were re-screened for *in vitro* inhibitory activity on *P. infestans* but this time on rye agar, which is the most suitable growth media for the pathogen (S10 Table). In the end, 5 fungal antagonistic candidates were selected for *ad planta* tests with *P. infestans* on tomato plants (Table 1). The criteria for selection of the 5 potential fungal antagonists included (i) literature evidence as a potential plant biocontrol agent: both broadly described genera, including *Trichoderma* and scarcely described genera, including *Pseudogymnoascus* were considered; (ii) ability to produce at least 4×10^5 ml^{-1} spores on standard fungal media (PDA) within 21 days of incubation at 25 ˚C; (iii) no negative effects on healthy tomato plants following leaf application.

Fig 1. Stepwise screening procedure of the fungal isolate collection for potential microbial antagonists (Modified from: Köhl et al. [32]).

Table 1. Selection of fungal biocontrol candidates for *ad planta* trials with *P. infestans* on tomato plants. Data on isolates inhibiting *A. solani*, *in vitro* are included.The putative genera were determined by BLAST analysis after partial sequencing of the ITS locus (approximately 560bp). Isolation parameters include media and temperature used for the isolation of fungi from plant samples. PDA: potato dextrose agar; OMA: oatmeal agar

	NCBI BLAST close relative (ITS loci)				Isolation parameters			Inhibitory activity on rye agar and PDA
**Isolate code**	**Genus match**	**Accession number**	**Sample source**	**Soil origin**	**Isolation temperature (°C)**	**Isolation Media**	**Spore count after 21 days of incubation at 25 °C on PDA**	***P. infestans***
Pf101	*Chaetomium* sp.	PX060209	Healthy rhizosphere	B	21	PDA	1.8×10^{6} spores/ml	yes
Pf 27	*Clonostachys* sp.	PX060190	Healthy rhizosphere	A	14	OMA	4.5×10^{6} spores/ml	yes
Pf 45	*Ctenomyces* sp.	PX060194	Diseased rhizosphere	A	21	PDA	2.2×10^{6} spores/ml	yes
Pf 23	*Pseudogymnoascus* sp.	PX060188	Diseased rhizosphere	A	21	OMA	2.6×10^{6} spores/ml	yes
Pf 131	*Trichoderma* sp.	PX060216	Diseased leaves	B	14	PDA	2.2×10^{6} spores/ml	yes

In vitro dual-culture assays of fungal isolates against *P. infestans* and *A. solani*

Two mycelial discs (5 mm) of 7-day-old agar cultures of the fungal isolates grown on PDA and 7-day-old cultures of *P. infestans* grown on rye agar (200 g rye; 3 g glucose; 12 g agar; 1000 ml distilled water) were simultaneously inoculated 4 cm opposite each other on PDA in the first screening of 165 isolates (S11 Table) and on rye agar in the second screening of 24 isolates (S10 Table) in standard Petri dishes. The control plates were inoculated with *P. infestans* only. Three Petri dishes were prepared for each isolate. The plates were incubated at 25 °C for 6-7 days, and fungal pathogen growth reduction indicated by inhibition zones around *P. infestans* was recorded (yes- inhibition zone present and no-inhibition zone absent). Fungal isolates that grew over the pathogen were also recorded as (yes) showing potential inhibitory effect. The screening for *A. solani*, precultured on PDA medium was performed in the same way as described for *P. infestans*.

Testing disease suppressive effects of selected fungal isolates *ad planta*

Five fungal isolates, *Chaetomium* sp. (Pf101), *Trichoderma* sp. (Pf131), *Pseudogymnoascus* sp. (Pf23) *Chlonostachys* sp. (Pf27), and *Ctenomyces* sp. (Pf45), were selected from the characterized fungal collection for plant trials (Fig 1). The isolates were grown on PDA for 21 days at 25 °C for spore production. Tomato seeds (`Red Robin´, Weigelt Samen) were sown in a 3:1 mixture of substrate (ProLine Potgrond, Klasmann-Deilmann, Germany) and sand, respectively, in the greenhouse. The seedlings were transplanted into planting pots (8 x 8 x 8.5 cm) containing the same soil mixture, 14 days after sowing. The plants were maintained in the greenhouse with demand-oriented watering and weekly fertilizer application using 0.2% (w/v) Hakaphos blau (Compo Expert, Münster, Germany). From each of the above-mentioned fungal candidates, a spore suspension of 4 x 10^5 ml^{-1} was adjusted in distilled water. Five ml were sprayed on the leaves of 5-week-old plants. Plants were kept in the

dark overnight. On the next day, the plants were spray-inoculated with 5 ml 2 x 10^4 ml^{-1} conidia of *P. infestans*. The trays were covered with lids to ensure 100% humidity and left in the dark overnight. The plants were placed in the growth chamber (Grow Bank, Plant Climatics, Germany) set to 16 h/8 h light/darkness, day temperature 21 °C, night temperature 16 °C, day humidity 65%, and night humidity 75%. Percentage diseased leaf area was visually recorded 7 days after spraying with fungal biocontrol candidates as described by Drenker et al. [33]. Six tomato plants were used per treatment, and three independent replicates of the plant trials were done. All plant trials followed a randomized design.

Molecular identification of isolates with inhibitory effects against *P. infestans*

Fungal DNA was extracted from 7-day-old cultures grown on PDA at 28 °C using the DNeasy Plant Mini kit (Qiagen, Germany) following the manufacturer's protocol. DNA quality was determined spectrophotometrically (NanoDrop 2000c; peqLab Biotechnologie GmbH, Erlangen Germany). The Internal Transcribed Spacer (ITS) region (approximately 560bp) was amplified using primers ITS 1F- 5'-TCCGTAGGTGAACCTGCGG-3', and ITS 4- 5'-TCCTCCGCTTATTGATATGC-3', as described in White et al. [36]. Briefly, a 25 µl PCR reaction mix was prepared for each sample, with reagents quantified as outlined by the manufacturer (Axon Labortechnik, Kaiserslautern). In the cases when PCR amplification was not successful, PCR reagents with a GoTaq® G2 Flexi DNA polymerase from Promega were used. The PCR cycling parameters were as follows: initial denaturation at 95 °C for 3 min; 40 cycles at 95 °C for 30 s (denaturation), 56 °C for 30 s (annealing), and 72 °C for 1 min (extension); final extension at 72 °C for 5 min. PCR products were purified using the ExoSAP-IT™ PCR Product Cleanup Reagent (Thermo Fisher Scientific) and 10 µl was used for sequencing (StarSEQ, Mainz), pre-mixed with ITS-1F. The Basic Local Alignment Search Tool (BLAST) was

used to identify potential genus matches by comparing the sequences of the isolates against the National Center for Biotechnology Information (NCBI) database (blastn, [37], and the most relevant hits based on the highest query coverage and percentage identity were recorded.

Molecular identification of *Chaetomium* species

The actual species of the *Chaetomium* isolate was evaluated by sequencing three identification loci according to Linkies et al. [38]. Internal transcribed spacer (primers ITS-1 and ITS-4 [36]; RNA polymerase II second largest subunit (primers RPB2AM-1bf (5'-CCA AGG TBT TYG TSA ACG G-3') and RPB2AM-7R (5'-GAA TRT TGG CCA TGG TRT CCA T-3') [39]; β-tubulin (primers T1 (5'-AACATGCGTGAGATTGTAAGT-3') and T22 (5'-TCTGGATGTTGTTGGGAATCC-3') [40] were employed with the following PCR conditions: Initial denaturation: 5 min at 95 °C, 40 cycles of denaturation: 30 s at 95 °C, 30 s of annealing at 55 °C and 45 s of extension at 72 °C, and a final extension of 5 min at 72 °C. The PCR product was visualized by 1% agarose gel electrophoresis. Cleaned PCR products using the ExoSAP-IT PCR Product Cleanup Reagent (Thermo Fisher, Berlin, Germany) were sequenced at StarSEQ (Mainz, Germany). Sequencing was performed in one direction and the species name was recorded as the highest BLAST hit. A consensus sequence of three separate PCR reactions was examined. The top BLAST hits for all three examined coding regions showed a 100% match to *Chaetomium subaffine* in both the ITS and β-tubulin regions, and a 99.63% match in the RNA polymerase II second largest subunit region.

DNA extraction and library preparation for the fungal microbiome analysis

Total DNA was extracted from 0.25 mg of samples of the phyllosphere, endosphere, and rhizosphere from both soil origins and both healthy and diseased plants in four replicates (48 samples in total) using the DNeasy Powersoil Pro kit (Qiagen, Germany) following the manufacturer's protocol. DNA yield and quality were checked photometrically (NanoDrop 2000c; peqLab Biotechnologie GmbH, Erlangen Germany) and samples were stored at -20 °C. Library construction and high-throughput amplicon sequencing of ITS region with primers ITS1F (5'-CTTGGTCATTTAGAGGAAGTAA-3' [41] and ITS2 (5'-GCTGCGTTCTTCATCGATGC-3' [36] was carried out by StarSEQ GmbH (Mainz, Germany). Illumina adapter sequences were added in a 1-step PCR approach. Libraries were normalized and sequenced on the Illumina MiSeq platform with a v3 Reagent Kit in 2 x 300 nt paired-end mode.

Analysis of ITS rDNA amplicon sequencing

Primers and adapters were removed from high-quality raw reads using the software Cutadapt (v.4.4, [42], implemented in the European Galaxy-Server [43](https://usegalaxy.eu/). Processing of paired-end reads was performed in R (v.4.2.1, [44] using the DADA2 pipeline (v.1.24.0, [45] following the adapted workflow for ITS sequences (DADA2 ITS Pipeline Workflow (1.8), https://benjjneb.github.io/dada2/ITS_workflow.html, accessed 25.1.2024). The obtained amplicon sequence variants (ASVs) were taxonomically assigned using NCBI BLAST (megablast, v.2.14.1, [37] with e-value 0.001 and the identity cut-off of 80% against the UNITE database (version 9, [46] containing ITS sequences in a Galaxy workflow as described in Fernandez-Grecco et al. [47]. Sequences identified as not fungal (= other eukaryotes) and sequences with less than five reads were removed from the dataset. This resulted in a final number of 2,124 ASVs and on average 124,032 quality reads per sample from 3,962,154 raw reads. Rarefaction curves indicated sufficient sequencing

depth (S1 Fig). Raw reads are deposited at the Sequence Read Archive (https://www.ncbi.nlm.nih.gov/sra) under the BioProject accession number PRJNA1273108. All scripts including detailed parameters and data to reproduce the figures for this study have been deposited in GitHub (https://github.com/TKuhl-Nagel/Phytophthora_challenged_tomato_microbiome.git).

Data analysis and statistics

Statistical analysis and data handling for ASVs was performed using R (v.4.2.1, [44]) with the R packages "tidyverse" (v.1.3.2, [48], "ggplot2" (v.3.5.1, [49]), "ggtext" (v.0.1.2, [50]), "dplyr" (v.1.1.4, [51]), "tibble" (v.3.2.1, [52]), "stringr" (v.1.5.1, [53]), "ARTool" (v.0.11.1, [54]), "vegan" (v.2.6-6.1, [55]) and "phyloseq" (v.1.44.0, [56]). To account for uneven sequencing depth, the dataset was rarefied by 1000 times random subsampling to the lowest number of reads (18,628 reads) (S1 Fig) and all further analyses were performed with the rarefied dataset as recommended by Schloss [57]. Significant effects of micro-compartment, soil origin, and disease status on alpha-diversity indices (Richness, Evenness, Shannon, Simpson) were calculated using the non-parametric aligned rank ANOVA because residuals failed the normality criteria based on the Shapiro test in the previously performed 3-way-ANOVA (S1-S4 Tables). After normalizing the dataset to relative abundance (%) Bray-Curtis distance was used to estimate beta-diversity and visualized using nonmetric multidimensional scaling (NMDS). The effects of micro-compartment, soil origin, and disease status on the fungal community composition were tested using PERMANOVA (10,000 permutations). Relative abundance was computed for each ASV within each sample by dividing ASV counts by the total count per sample. Square bar plots were generated to represent the most abundant taxa (phylum, genus, and ASV level) based on their relative abundance in the whole dataset. Differences in their relative abundances based on soil origin were calculated for each micro-

compartment using ANCOM-BC2 (v2.1.2, [58]) with p-value correction via the Benjamini-Hochberg method. The data set was filtered for rows with 0 ASVs prior to ANCOM-BC2 (Analysis of Compositions of Microbiomes with Bias Correction) to reveal differentially abundant taxa considering the two soil origins. ANCOM-BC2 also calculates the bias-corrected log-fold change (LFC) for each taxon between the two soil conditions to confirm that any taxon found only in one condition and completely absent in the other is a structural zero (sz) rather than rarely detected low-abundant taxa. LFC represents the ratio of the abundance of taxon between two conditions on a logarithmic scale. Negative LFC values indicate higher abundance in Soil A, while positive values indicate higher abundance in Soil B. ASVs classified as structural zero are exclusively present in one soil origin and are represented by strongly negative LFC (approaching $-\infty$) and strongly positive LFC (approaching $+\infty$). In order to compare the isolated living fungal species to the fungal community of the rhizosphere, we mapped the ASV sequences (~250 bp) to the isolate sequences (~500 bp) with 100% identity and at least 50% query coverage using NCBI BLAST. Afterwards, isolates were evaluated for their presence among the most abundant taxa and/or the differentially abundant taxa in the microbial community. Statistics for *ad planta* experiments and generation of plots were performed in R (versiolastn 4.4.2). Data on diseased leaf area (%) were presented as mean values ± standard error (SEM) computed from 6 biological replicates. The *ad planta* inhibitory effect of the fungal candidates against *P. infestans* was evaluated using a two-way analysis of variance (ANOVA) as the data was normally distributed according to Shapiro-Wilk test results. Significant differences between means were evaluated by Tukey's test at $P \leq 0.05$. Plant experiments were conducted in three independent repetitions, analyzed separately. Raw data from the three plant trials, and the associated R scripts including detailed parameters and data to reproduce the figures for this study have been deposited in GitHub (https://github.com/TKuhl-Nagel/Phytophthora_challenged_tomato_microbiome.git).

Results

Comparison of soil properties from Wiesbaden and Rüsselsheim in the the Rhine-Main area of Germany

Both soils were loam soils with comparable measures in terms of physical properties including humus level and organic carbon content (Table 2). Soil A had a slightly higher pH than soil B. While both soils were sufficiently supplied with macronutrients such as phosphorus, potassium, magnesium, and trace elements, they had low iron content. Tomato plants grown in soil A demonstrated a significant difference in weight between healthy and diseased conditions following 14 days of infection with *P. infestans* (Fig 2A), with diseased plants showing a significantly lower fresh weight. In soil B, there was no significant difference in fresh weight between healthy and diseased plants. However, healthy plants grown in both soil origins had comparable measures in weight. In addition, plants grown in both soils showed slight differences in the degree of symptom development upon inoculation with *P. infestans* (Fig 2B). The results indicate that the selected soils have comparable properties and are suitable as a resource for plant-beneficial fungi.

Table 2: Evaluation of the physiochemical parameters for soil A and soil B. The soil samples were analyzed according to VD-Lufa methods [59].

**Soil sample**	**A**	**B**	**A**	**B**
Soil origin	Wiesbaden	Rüsselsheim		
**Soil physical properties**	**Measured value**		**Evaluation**	

Soil type	Loam	Loam	Heavy	Heavy
pH value	7.5	7.2	Increased	Sufficient
Humus class	H2	H2	Slightly humic	Slightly humic
Organic carbon	0.6 - < 1.2	0.6 - < 1.2	Slightly humic	Slightly humic
**Main nutrients**	**Measured value** (mg/100 g)		**Supply rate**	
P_2O_5	119	44.0	High	High
K_2O	34.5	36.0	Above requirement	Above requirement
MgO	145	39.4	High	Above requirement
**Trace elements**	**Measured value** (mg/kg)		**Supply rate**	
Boron	1.99	1.26	Above requirement	Above requirement
Copper	5.52	3.23	High	High
Iron	349	373	Low	Low
Zinc	30.5	10.8	High	High

Fig 2. Effect of *P. infestans* on tomato plants grown in two different soil origins (A and B), in terms of (A) plant fresh weight and (B) diseased leaf area (%). The two soils were collected from two organic growers from Domäne Mechthildshausen, Wiesbaden (soil A) and Solidarische

Landwirtschaft, Rüsselsheim (soil B). Plants were inoculated with *P. infestans* conidial suspension (2×10^4 conidia/ml) 5 weeks after sowing. Tomato plants inoculated with distilled water served as the healthy control. Four tomato plants were used for each treatment (n=4). Data were collected 14 days after inoculation with *P. infestans*. Means were compared with two-way ANOVA ($p \leq 0.01$) followed by Tukey's post-hoc test ($p \leq 0.05$). Significant differences are shown by letters (a, b).

Characterization of the fungal culture collection derived from tomato rhizosphere and phyllosphere

The isolation process of fungi from different samples yielded 246 isolates (Fig 1). In a stepwise screening approach described in the methods section, the number was reduced to 61 isolates (S9 Table) with promising characteristics as biocontrol agents against *P. infestans*. The *in vitro* antagonistic activity of the fungal isolates against *P. infestans* served as one selection criterion (Fig 3). Additionally, parameters that are advantageous for later product development and approval or registration as a biocontrol product were also taken into account, namely the inability to grow at 37 °C, easy spore induction, and ability to survive on standard growth media. Out of the 61 identified isolates with antagonistic effects against *P. infestans*, the rhizosphere yielded 45 fungal isolates, of which 24 and 21 isolates derived from healthy and diseased rhizosphere, respectively. Another 16 isolates were recovered from the phyllosphere, of which eleven isolates derived from diseased phyllosphere compared to five isolates from healthy phyllosphere. All candidates were identified on the genus level by partial sequencing of the ITS locus.

Fig 3. Activity of selected fungal isolates against *P. infestans*. Dual-culture assays showing representative fungal candidates (right part of each Petri dish) and *P. infestans* (left part of each Petri

dish). Assays were performed on PDA with both fungal plugs placed 4 cm apart on Petri dishes. The results were recorded after 7 days at 25 °C for n=3 plates.

The genus *Penicillium* constituted the largest share (27 isolates), followed by *Trichoderma* (6 isolates), *Agrostalagmus* (4 isolates), *Chlonostachys, Mortierella,* and *Pseudogymnoascus* (3 isolates each), (S9 Table). All other genera were only present once or twice. Based on literature research, potential health concerns and plant pathogenic isolates, such as *Penicillium*, *Fusarium*, and *Acrostalagmus* were removed, ending up with 24 remaining candidates (Table 1). Among the 24 fungi, *Mortierella* sp., *Pseudogymnoascus* sp., *Trametes* sp., and *Trichoderma* sp. are notable for their potential to inhibit both *P. infestans* and *A. solani.* Among the antagonists tested for *ad planta* disease suppressive effects on *P. infestans* included *Pseudogymnoascus* sp. and *Trichoderma* sp.. However, no further tests were performed with *A. solani* as most of the antagonists showed weak inhibition potential via *in vitro* confrontation assays. Intriguingly, a majority of the selected fungi (17 isolates) originated from samples of tomato plants grown in soil A.

Fungal isolates show disease-suppressive effects *ad planta* against *P. infestans*

Five of the remaining 24 fungal candidates were selected for exemplary tomato plant trials evaluating suppressive characteristics against *P. infestans*. *Pseudogymnoascus* sp. (isolate Pf23), *Chlonostachys* sp. (Pf27), and *Ctenomyces* sp. (Pf45), *Chaetomium* sp. (Pf101), and *Trichoderma* sp. (Pf131), were chosen based on visible mycelial growth towards the edges of the Petri dish on standard media, abundant spore production (at least 4 x 10^5 ml^{-1} spores in standard fungal media (PDA)), and literature

research. The isolates demonstrated varying levels of disease-suppressive potential in growth chamber plant trials (Fig 4). *Chaetomium* sp. (Pf101) significantly suppressed *P. infestans* across three independent replicates of the trial, as evidenced by the lower percentage of diseased leaf area compared to the positive control. *Chlonostachys* sp. (Pf27) significantly suppressed *P. infestans* in the first and second trials, whereas *Pseudogymnoascus* sp. (Pf23) significantly suppressed *P. infestans* in the second and third trials. *Ctenomyces* sp. (Pf45) was able to inhibit *P. infestans* once and *Trichoderma* sp. (Pf131) did not significantly inhibit symptoms of *P. infestans*, although the percentage of diseased leaf area was slightly lower compared to the positive control.

Fig 4. **Efficacy of selected fungal isolates against *P. infestans* on tomato plants across three trials.** Spore suspensions (4×10^5 CFU/ml) prepared from *Chaetomium* sp. (Pf101), *Trichoderma* sp. (Pf131), *Pseudogymnoascus* sp. (Pf23), *Chlonostachys* sp. (Pf27), and *Ctenomyces* sp. (Pf45) were preventively applied on tomato leaves, 24 hours before inoculation with *P. infestans* (2×10^4 ml^{-1} conidia). Tomato plants inoculated with distilled water served as the negative control (Negative_control). Tomato plants inoculated with *P. infestans* only served as the positive control (Positive_control). Tomato plants treated with Cuprozin progress (0.52%) and inoculated with *P. infestans* served as the chemical control (Copper standard). Six tomato plants were used for each treatment (n=6). Percentage disease leaf area (%) was determined 7 days after inoculation with *P. infestans*. The letters above the boxes show significant differences across treatments ($p \leq 0.05$) according to Tukey HSD tests following ANOVA on diseased leaf area data. The three trials represent three independent replicates.

Soil origin influences the fungal community in tomato plants

The micro-compartment (endosphere, phyllosphere, rhizosphere), soil origin, disease status, and their interaction significantly influenced the alpha diversity of the fungal microbiome (ANOVA, Fig 5A-D, S1-S4 Tables). Rhizosphere samples had the highest alpha-diversity indices indicating the presence of many species and with a rather homogeneous distribution compared to endosphere and phyllosphere. The disease status of the plant had a significant influence on the alpha-diversity only in the phyllosphere (ANOVA, S1-S4 Tables), which shows significant differences in the within-sample diversity between healthy and diseased plants mainly in soil B (Fig 5A-C). Besides, *P. infestans* had an effect on fungal species richness in the endosphere of plants grown in soil A, where infection reduced fungal species richness compared to healthy samples (Fig 5A). These results show that leaf infection with *P. infestans* led to changes in the within-sample microbiome composition of the phyllosphere, although these changes are only significant in plants grown in soil B. In contrast, the endosphere and rhizosphere were mainly not significantly affected by microbial diversity after inoculation with *P. infestans*.

Fig 5. The alpha-diversity indices species richness (A), Shannon diversity index (B), evenness (C), and Simpson index (D) of the fungal microbiome in samples from phyllosphere, endosphere, and rhizosphere from tomato plants grown in two soil origins (soil A and soil B). Plants were grown in two different soil origins (A and B) and were inoculated with *P. infestans* (diseased, (turquoise plots) or remained untreated (healthy, red plots) 14 days before sampling was done. Four tomato plants were used for each treatment (n=4). Values of alpha-diversity indices are shown, corresponding p-values are written above each comparison. Means were compared with a Kruskal Wallis test, with a significance threshold set at $p \leq 0.05$.

The micro-compartment, soil origin, and their interaction were identified as the driving factors of the fungal ß-diversity (PERMANOVA $p \leq 0.001$, S5 Table, Fig 6), whereas *P. infestans* infection had no significant influence on the fungal community composition. Phyllosphere samples clustered more distant from each other compared to rhizosphere and endosphere.

Fig 6. Microbial beta-diversity of different tomato micro-compartments visualized using an NMDS plot. The fungal microbiome composition was determined using Bray–Curtis community dissimilarities. Plants were grown in two soil origins (A and B) and inoculated with *P. infestans* 14 d prior to sample collection from the rhizosphere, phyllosphere, and endosphere of healthy and diseased plants. ANOSIM was used to determine significant differences ($p \leq 0.001$, S5 Table).

Based on these results we focused on the effect of soil origin on the fungal microbial community in the different micro-compartments because beta diversity analysis showed no significant difference between diseased and healthy plants. In each micro-compartment, we identified shared and unique taxa between the two soils (Fig 7), using ANCOM-BC2 (Analysis of Compositions of Microbiomes with Bias Correction). Rhizosphere samples of the two soil origins had overall more taxa compared to the phyllosphere and the endosphere, which confirms previous results. Interestingly, samples from soil B harbour more taxa compared to soil A across the three micro-compartments, indicating a soil-specific effect on fungal composition at the phylum, genus, and amplicon sequence variant (ASV) levels. At the same time, a substantial number of taxa were shared between plant samples from soil A and soil B across the three taxonomic levels (phylum, genus, ASV).

Fig 7: Fungal taxonomic distribution of the microbiome analyses at the phylum, genus, and amplicon sequence variant (ASV) levels in the rhizosphere, phyllosphere, and endosphere of soil

A and soil B. The overlapping region represents taxa that are shared between soil A and soil B. Numbers inside each section represent the number of taxa at each level (phylum, genus, or ASV). The Venn diagram is based on the analysis of relative abundance with ANCOM-BC2.

Further analysis of the most abundant taxa in plant samples from the two soil origins showed that the phylum Ascomycota had the highest relative abundance in the whole data set (Fig 8A). Although other phyla showed variation between the two soil origins, only Basidiomycota, Rozellomycota, and Mortierellomycota significantly differed in abundance in individual micro-compartments. At the genus level, *Botryotrichum*, *Geosmithia*, *GS11_gen_Incertae_sedis*, and *Pseudopyrenochaeta* were more abundant in soil A, whereas *Aspergillus*, *Brunneomyces*, and *Verticillium* were more abundant in soil B in one or more micro-compartments (Fig 8B). *Cladosporium* and *Acremonium* were the top abundant genera in the phyllosphere, while *Colletotrichum* and *Pseudopyrenochaeta* were the most abundant group in the endosphere. The rhizosphere had a high proportion for an unknown genus, *GS11_gen_Incertae_sedis*.

The most abundant ASVs in the whole dataset were from three phyla, with a majority from Ascomycota, followed by Olpidiomycota, and Rozellomycota (Fig 8C, Table 3). The differentially abundant ASVs between the two soil origins across the three micro-compartments were linked to *Cladosporium*, *Geosmithia*, *Olpidium*, *Pseudopyrenochaeta*, *Verticillium*, and unknown genus (*GS11_gen_Incertae_sedis*) (Fig 8C, Table 3).

Fig 8. Diversity of the most abundant fungal taxa at phylum (A), genus (B), and (C) ASV levels between plants grown in soil A and soil B across the three micro-compartments (endosphere, phyllosphere, and rhizosphere). Others represent all taxa with less than 9%, 6%, and 5.5% abundance at phylum, genus, and ASV levels, respectively. Bars represent the mean relative abundance (%) of

each phylum genus, and ASV. Error bars indicate standard deviation. Asterisks indicate a significant difference in abundance between samples from soil origins A and soil B inferred at p-value ≤ 0.05 according to Benjamini-Hochberg (BH) correction, which controls for false discovery rate (FDR) analyzed using ANCOM-BC2 (Analysis of Compositions of Microbiomes with Bias Correction).

Comparison of culture collection and fungal microbiome composition

In order to identify overlaps between the culture collection of living isolates and the microbiome analysis, we mapped ITS sequences of selected fungal isolates that demonstrated *in vitro* inhibitory effects against *P. infestans* to fungal ASVs in the microbiome of tomato plants grown in soils A and B. Of the 61 fungal isolates, 42 (*Penicillium* (21), *Trichoderma* (5), *Acrostalagmus* (4), *Clonostachys* (2), *Pseudogymnoascus* (2), *Cladosporium* (2), *Bionectriaceae* (2), *Ctenomyces* (2), *Chaetomium* (1) and *Humicola* (1)) showed 100% identity with the ASV sequences.

Cladosporium linked to isolates Pf179 and Pf242 from our culture collection was the only genus detected among the top 15 ASVs in terms of relative abundance (Table 3). Of the ASVs that matched our fungal collection, only ASV14 identified as *Cladosporium* sp. was differentially abundant in the rhizosphere between the two soil origins (Fig 8C, Table 3), with higher abundance in soil A. The matching ASVs were further evaluated for exclusive presence (also referred to as structural zeros) in plant samples from either soil origin. Intriguingly, ASVs identified as the genera *Mortierella, Ctenomyces,* and *Pseudogymnoascus* were exclusively present in the rhizosphere of plant samples from soil A, whereas ASVs identified as the genus *Chaetomium* were exclusively found in samples of plants grown in soil B (S6 Table). Phyllosphere and endosphere samples showed a similar pattern of exclusive taxonomic presence, with plants cultivated in soil A enriched with ASVs identified as the genus *Ctenomyces* and those from plants grown in soil B enriched solely with ASVs identified as *Chaetomium* (S7-S8 Table).

Table 3: Taxonomic affiliation of the top most abundant fungal ASVs in the microbiome of tomato plants in the whole data set. The ASVs were taxonomically assigned using NCBI BLAST (megablast, v.2.14.1) with an e-value of 0.001 and the identity cut-off of 80% against the UNITE database (version 9, [46]) containing ITS sequences in a Galaxy workflow.

ASVid	Taxonomic affiliation at phylum level	Taxonomic affiliation at genus level and potential species
ASV1	Ascomycota	*Cladosporium sphaerospermum*
ASV2	Ascomycota	*Plectosphaerella ramiseptata*
ASV3	Ascomycota	*Colletotrichum coccodes*
ASV4	Ascomycota	*Acremonium sclerotigenum*
ASV5	Ascomycota	*Berkeleyomyces rouxiae*
ASV6	Rozellomycota	GS11_gen_Incertae_sedis
ASV7	Ascomycota	*Verticillium dahliae*
ASV8	Ascomycota	*Pseudopyrenochaeta lycopersici*
ASV10	Ascomycota	*Pseudopyrenochaeta terrestris*
ASV12	Olpidiomycota	*Olpidium* sp.
ASV13	Olpidiomycota	*Olpidium* sp.
ASV14	Ascomycota	*Cladosporium halotolerans*
ASV16	Ascomycota	*Geosmithia* sp.
ASV28	Ascomycota	*Cladosporium sphaerospermum*

Discussion

In times of increasing awareness of sustainable use of pesticides, microorganisms gain more and more importance as biocontrol agents of plant diseases [60,61]. Our study characterized cultivable fungi

from various plant micro-compartments as the initial step towards discovering fungal biocontrol agents against *P. infestans*. Additionally, we employed a fungal microbiome analysis to evaluate the potential importance of our fungal candidates within the microbiome. The initial number of 246 isolates obtained from the phyllosphere, endosphere, and rhizosphere of tomato plants was reduced to 24 potential biocontrol candidates remaining for plant trials by utilization of the step-wise screening procedure described by Köhl et al. [32]. The multi-step framework employs several steps to screen a high number of isolates for candidates fulfilling requirements for antagonists in commercial use, for example regarding efficacy, ecology, production, safety, and environmental risks. Schisler and Slininger [62] also included the above commercial parameters to select viable bacterial antagonists against *Gibberella pulicaris*, a potato dry rot causing pathogen.

The rhizosphere yielded more candidate isolates than the phyllosphere and endosphere, which concurs with previous findings that described the rhizosphere as a highly competitive habitat and a stable reservoir for beneficial microbes in terms of pathogen suppression, nutrient acquisition, and host defense functions [24,63]. Additionally, as a nutrient-rich environment, partly caused by plant root exudates [64], the rhizosphere supports a diverse microbial community, including beneficial fungi with potential biocontrol characteristics. Low microbe numbers recovered from the phyllosphere are linked to the specific nature of this habitat which is characterized by highly varying conditions that support only the survival of a limited number of microbes [65]. Recovering more fungal candidates from diseased phyllosphere samples suggests the enrichment of fungal microbial antagonists in pathogen-prevalent micro-compartments. Phyllosphere-associated microbes have been described with various functions, including plant growth promotion and pathogen inhibition [66], and nitrogen fixation [67], and can be causative agents for plant diseases [66]. The genera *Penicillium, Trichoderma, Chlonostachys, Mortierella*, and *Pseudogymnoascus* dominated our collection of potential microbial antagonists based on *in vitro* tests. A majority of these fungal groups have been adequately described as potential biocontrol agents. Culture filtrates of *Penicillium griseofulvum* demonstrated inhibitory

effects on the growth of *P. infestans in vitro* [68]. But since *Penicillium* [69] species have been frequently reported as mycotoxin producers or as otherwise harmful in regard to food safety and consumer health [70], all *Penicillium* sp. isolates were eliminated from subsequent steps at an early screening stage. *Trichoderma* has been broadly investigated as a BCA owing to its ability to thrive in diverse soil habitats and outcompete pathogens for space and nutrients among other biocontrol mechanisms [71]. *Trichoderma* is also linked to other plant-beneficial functions such as phosphate solubilization and could be vital for the overall fitness of tomato plants through plant growth promotion [72]. Particular strains of *Chlonostachys rosea* [73], a tomato stem endophyte, have been reported to significantly reduce early blight caused by *A. solani* [74] and *B. cinerea* [75] in tomatoes. Products based on *Trichoderma* sp. [76] and *C. rosea* [77] are approved as fungicides in Europe. Moreover, there is limited information on the isolation of *Pseudogymnoascus* species from tomato plants, but this fungal genus is among the most abundant taxa in tomato root microbiome through amplicon sequencing [78].

While *in vitro* effects of certain microbial species or strains may not necessarily be reflected in the *in vivo* effects, the five selected fungal candidates tested in *ad planta* assays showed varying disease-suppressive efficiency against *P. infestans*. The candidate antagonists from *Chaetomium* sp., *Chlonostachys* sp., and *Pseudogymnoascus* sp. demonstrated significant disease-suppressive effects in at least two separate plant trials, validating the feasibility of our approach in identifying new or improved biocontrol agents. It is worth noting that the findings on *Pseudogymnoascus* contribute substantially to the knowledge of this genus in the field of plant pathology as a potential biocontrol agent. *Chaetomium* species, including *Chaetomium cochliodes* and *Chaetomium aureum*, are known to produce antifungal secondary metabolites against tomato pathogens such as *A. solani*, *B. cinerea*, and *P. infestans* [38,79]. However, other species of *Chaetomium* induce human pathogenic effects linked to systemic infections and allergic reactions [80]. Our isolate was confirmed to be *Chaetomium*

subaffine after sequencing three identification loci (as described in the methods). To the best of our knowledge, there is currently no data on potential health or environmental risks associated with *C. subaffine*. Liu et al. [81] revealed the potential mycolytic effects of *C. subaffine* LB-1 on phytopathogenic fungi *B. cinerea* and *A. solani* by altering their cell membrane and cell wall. Reduced biocontrol efficacy by other groups, including *Trichoderma sp*. (Pf131) and *Ctenomyces sp*. (Pf45) across trials could be linked to environmental or procedural effects. For instance, soil-originated beneficial microbes such as *Trichoderma* when introduced to the leaves and *vice versa* may not survive or could cause negative interactions with the native community of microorganisms on the leaf reducing the efficacy [3]. Further processing steps such as drying or adding stabilizers might surely change the outcome but have not been investigated here. Different application methods such as the use of soil additives and fertilizers or a regular leaf application allowing for preventive and curative effects might also increase efficacy [82] but have not been tested yet.

High-resolution microbiome profiling techniques have improved the research on cultivable fungal species with plant-beneficial effects, providing crucial data for the targeted application of antagonists to ensure improved efficacy [83,84]. One example of this approach is demonstrated by Sahu et al. [85] who integrated culture methods and metabarcoding to profile the microbiome of the rice phyllosphere, revealing the key microbial communities for blast pathogen control. Similarly, we detected the presence of some of our fungal candidate antagonists for *P. infestans*, including *Cladosporium* among the highly abundant taxa and differential taxa in the tomato plant microbiome dataset. Although some *Cladosporium* species are plant pathogens [86,87], there are plant-beneficial species promoting growth[88,89]. This genus could reflect the core microbiome which has received a lot of interest in recent years, with multiple studies revealing core microorganisms, including bacteria, fungi, and archaea playing different roles in various plant hosts[90–92]. The dominance of specific fungal taxa, for instance, the exclusive presence of *Mortierella*, *Ctenomyces*, and *Pseudogymnoascus* in the rhizosphere samples from soil A and *Chaetomium* from rhizosphere samples in soil B may be a

reflection of their beneficial activities to ensure a healthy plant microbiome [83]. In the rhizosphere, root exudates released due to pathogen invasion trigger plants to recruit microorganisms that are involved in specific functions, including direct suppression of the pathogens [93,94]. The endosphere represents the internal phytomicrobiome enriched with specifically adapted microorganisms that interact intimately [95]. The phyllosphere is linked to the atmosphere and interacts continuously with the air microbiome [96]. The fact that the fungal candidate antagonists are naturally present within the tomato plant and are effective against *P. infestans* highlights their potential as BCAs [97,98]. Therefore, knowledge of the ecological role of fungal biocontrol candidates in their native microbiomes can provide insights into the selection of improved and effective fungal agents against *P. infestans*. Enhanced knowledge of how pathogen infection of a host plant alters soil microbial communities may pave the way for the use of microbial antagonists in the long-term management of plant diseases [99]. Therefore, our approach to microbiome characterization provides a more complete picture of how *P. infestans* and distinct soil sources may influence the composition of the tomato fungal community. Although *P. infestans* did not alter the overall fungal microbiome composition of tomato plants, significant changes in fungal alpha diversity were evident in specific micro-compartments, particularly the phyllosphere. Previous research on *P. parasitica* (closely related to *P. infestans*) revealed changes in the bacterial microbiota in tomato roots upon infection, whereas the fungal microbiota remained relatively stable [100]. These changes in fungal composition could also be attributed to distinct soil physiochemical parameters as we cultivated tomatoes in soils from two different locations in the Rhine-Main area [101]. Studies have shown that pH plays a key role in determining the composition of bacteria and fungi in the rhizosphere and bulk soil [102,103]. Also, soils with sufficient nutrient amounts provide optimal habitat for beneficial microbial communities to outperform pathogens [104].

Taken together, we present a strategic approach that enhances the likelihood of identifying fungal candidates with both strong biocontrol potential and commercial viability. The tested fungal biocontrol candidates, including *Chaetomium* sp., *Chlonostachys* sp., and *Pseudogymnoascus* sp. isolated from different micro-compartments of tomato plants grown in two soil origins inhibited *P. infestans* and *A.solani in vitro* and reduced *P. infestans*-related disease symptoms *ad planta*. We have also demonstrated that *P. infestans* infection of tomato plants influences the fungal community composition at the micro-compartment level, particularly on the phyllosphere rather than on the overall plant microbiome. However, the effect of soil origin on fungal community composition is significant considering the overall microbiome of tomato plants. The high abundance and differential abundance of specific genera, in our case *Cladosporium* indicates the potential beneficial role of this genus in tomato plant health as initial treatment showed no negative effects on the plant. In subsequent steps, we plan to test the suppressive effects of our *Chaetomium subaffine* in open-field plant trials. Other fungal candidates with variable efficacy against *P. infestans* will be subjected to optimization steps and used as consortia. Lastly, we intend to incorporate both curative and preventive application methods in future plant trials to achieve better disease-suppressive effects from the fungal candidates in a controlled growth room and greenhouse conditions. These findings improve our knowledge of the link between fungal biological control agents and plant microbiome, providing valuable ecological data in identifying effective antagonists suited for sustainable control of *P. infestans*.

Acknowledgements

The authors thank Anja Götzmann, Julia Herzberg, and Ramona Mittag for technical support and Julian Maier for assistance with the analysis of plant trial data. Christian Fetzer from Landesbetrieb Landwirtschaft Hessen (LLH) established the contact to the growers whose soil was used for the study, which is kindly acknowledged.

Funding

P.O. received funding from Deutscher Akademischer Austauschdienst (DAAD) doctoral scholarship (award no. 91832749). The funders did not contribute to the study design, data collection and analysis, decision to publish, or preparation of the manuscript.

Author Contributions

Conceptualization: Philemon Orwa, Johannes A. Jehle, Romano Mwirichia, Ada Linkies.

Data curation: Philemon Orwa, Theresa Kuhl-Nagel, Rosa Meinhold-Ernst, Arne Seyer.

Formal analysis: Philemon Orwa, Theresa Kuhl-Nagel.

Funding acquisition: Philemon Orwa, Johannes A. Jehle, Romano Mwirichia, Ada Linkies.

Investigation: Philemon Orwa, Rosa Meinhold-Ernst, Arne Seyer.

Methodology: Philemon Orwa, Theresa Kuhl-Nagel, Rosa Meinhold-Ernst, Arne Seyer.

Project administration: Johannes A. Jehle, Ada Linkies.

Resources: Johannes A. Jehle, Ada Linkies.

Supervision: Theresa Kuhl-Nagel, Johannes A. Jehle, Romano Mwirichia, Ada Linkies.

Visualization: Philemon Orwa, Theresa Kuhl-Nagel.

Writing – original draft: Philemon Orwa.

Writing – review & editing: Theresa Kuhl-Nagel, Johannes Romano Mwirichia, Ada Linkies.

References

1. Ali MY, Sina AAI, Khandker SS, Neesa L, Tanvir EM, Kabir A, et al. Nutritional composition and bioactive compounds in tomatoes and their impact on human health and disease: A

review. Foods. 2021;10(1).

2. Panno S, Davino S, Caruso AG, Bertacca S, Crnogorac A, Mandić A, et al. A review of the most common and economically important diseases that undermine the cultivation of tomato crop in the mediterranean basin. Agronomy. 2021;11(11): 1–45.

3. Hashemi M, Tabet D, Sandroni M, Benavent-Celma C, Seematti J, Andersen CB, et al. The hunt for sustainable biocontrol of oomycete plant pathogens, a case study of *Phytophthora infestans*. Fungal Biol Rev. 2022;40: 53–69.

4. Cooke LR, Schepers HTAM, Hermansen A, Bain RA, Bradshaw NJ, Ritchie F, et al. Epidemiology and integrated control of potato late blight in Europe. Potato Res. 2011;54(2): 183–222.

5. Lehsten V, Wiik L, Hannukkala A, Andreasson E, Chen D, Ou T, et al. Earlier occurrence and increased explanatory power of climate for the first incidence of potato late blight caused by *Phytophthora infestans* in Fennoscandia. PLoS One. 2017;12(5): 1–21.

6. Fry WE, Birch PRJ, Judelson HS, Grünwald NJ, Danies G, Everts KL, et al. Five reasons to consider *Phytophthora infestans* a reemerging pathogen. Phytopathology. 2015;105(7): 966–981.

7. Haverkort AJ, Struik PC, Visser RGF, Jacobsen E. Applied biotechnology to combat late blight in potato caused by *Phytophthora infestans*. Potato Res. 2009;52(3): 249–264.

8. Kamoun S, Furzer O, Jones JDG, Judelson HS, Ali GS, Dalio RJD, et al. The top 10 oomycete pathogens in molecular plant pathology. Mol Plant Pathol. 2015;16(4): 413–434.

9. Wan NF, Fu L, Dainese M, Kiær LP, Hu YQ, Xin F, et al. Pesticides have negative effects on non-target organisms. Nat Commun. 2025;16(1): 1–16.

10. Wang Y, Tyler BM, Wang Y. Defense and counterdefense during plant-pathogenic oomycete infection. Annu Rev Microbiol. 2019;73: 667–696.

11. European Parliament. Directive 2009/128/EC of the european parliament and the council of 21

October 2009 establishing a framework for community action to achieve the sustainable use of pesticides. October. 2009;309: 71–86.

12. Pieterse CMJ, Zamioudis C, Berendsen RL, Weller DM, Van Wees SCM, Bakker PAHM. Induced systemic resistance by beneficial microbes. Annu Rev Phytopathol. 2014;52: 347–375.

13. Spadaro D, Droby S. Development of biocontrol products for postharvest diseases of fruit: The importance of elucidating the mechanisms of action of yeast antagonists. Trends Food Sci Technol. 2016;47: 39–49.

14. Raaijmakers JM, Mazzola M. Diversity and natural functions of antibiotics produced by beneficial and plant pathogenic bacteria. Annu Rev Phytopathol. 2012;50: 403–424.

15. Khabbaz SE, Zhang L, Cáceres LA, Sumarah M, Wang A, Abbasi PA. Characterisation of antagonistic *Bacillus* and *Pseudomonas* strains for biocontrol potential and suppression of damping-off and root rot diseases. Ann Appl Biol. 2015;166(3): 456–471.

16. Chowdappa P, Mohan Kumar SP, Jyothi Lakshmi M, Upreti KK. Growth stimulation and induction of systemic resistance in tomato against early and late blight by *Bacillus subtilis* OTPB1 or *Trichoderma harzianum* OTPB3. Biol Control. 2013;65(1): 109–117.

17. De Silva NI, Brooks S, Lumyong S, Hyde KD. Use of endophytes as biocontrol agents. Fungal Biol Rev. 2019;33(2): 133–148.

18. Rai S, Solanki MK, Solanki AC, Surapathrudu K. Biocontrol potential of *Trichoderma* spp.: current understandings and future outlooks on molecular techniques. In: Ansari RA, Mahmood I, editors. Plant Health Under Biotic Stress: Volume 2: Microbial Interactions. Singapore: Springer Singapore; 2019. p. 129–160.

19. Luo C, He Y, Chen Y. Rhizosphere microbiome regulation: Unlocking the potential for plant growth. Curr Res Microb Sci. 2025;8: 100322.

20. Bakker PAHM, Pieterse CMJ, de Jonge R, Berendsen RL. The soil-borne legacy. Cell. 2018;172(6): 1178–1180.
21. Gush S, Lebre P, Couthino TA, Cowan DA, van der Waals JE. Disentangling shifts in the soil microbiome of potatoes infected with *Rhizoctonia solani* anastomosis group 3-pt in search of potential biocontrol agents. Phytobiomes J. 2024;8(2): 128–142.
22. Raaijmakers JM, Paulitz TC, Steinberg C, Alabouvette C, Moënne-Loccoz Y. The rhizosphere: A playground and battlefield for soilborne pathogens and beneficial microorganisms. Plant Soil. 2009;321(1–2): 341–361.
23. Jones DL, Hinsinger P. The rhizosphere: Complex by design. Plant Soil. 2008;312(1–2):1–6.
24. Berendsen RL, Pieterse CMJ, Bakker PAHM. The rhizosphere microbiome and plant health. Trends Plant Sci. 2012;17(8): 478–486.
25. Bakker PAHM, Berendsen RL, Doornbos RF, Wintermans PCA, Pieterse CMJ. The rhizosphere revisited: Root microbiomics. Front Plant Sci. 2013;4: 1–7.
26. Jayne B, Quigley M. Influence of arbuscular mycorrhiza on growth and reproductive response of plants under water deficit: a meta-analysis. Mycorrhiza. 2014;24(2): 109–119.
27. Baum C, El-Tohamy W, Gruda N. Increasing the productivity and product quality of vegetable crops using arbuscular mycorrhizal fungi: A review. Sci Hortic. 2015;187: 131–141.
28. El Komy MH, Saleh AA, Eranthodi A, Molan YY. Characterization of novel *Trichoderma asperellum* isolates to select effective biocontrol agents against tomato *Fusarium* wilt. Plant Pathol J. 2015;31(1): 50–60.
29. Treseder KK, Lennon JT. Fungal traits that drive ecosystem dynamics on land. Microbiol Mol Biol Rev. 2015;79(2): 243–262.
30. Brar B, Bala K, Singh B, Pardeep S, Sadh K, Singh J. Bio - boosting agriculture: Harnessing the potential of fungi - bacteria - plant synergies for crop improvement. Discov Plants. 2024; 1(1): 21.

31. Kim H -Y., Choi GJ, Lee HB, Lee S -W., Lim HK, Jang KS, et al. Some fungal endophytes from vegetable crops and their anti-oomycete activities against tomato late blight. Lett Appl Microbiol. 2007;44(3): 332–337.

32. Köhl J, Postma J, Nicot P, Ruocco M, Blum B. Stepwise screening of microorganisms for commercial use in biological control of plant-pathogenic fungi and bacteria. Biol Control. 2011;57(1): 1–12.

33. Drenker C, El Mazouar D, Bücker G, Weißhaupt S, Wienke E, Koch E, et al. Characterization of disease-suppressive isolate of *Lysobacter enzymogenes* with broad antagonistic activity against bacterial, oomycetal and fungal pathogens in different crops. Plants. 2023;12(3).

34. Wieland G, Neumann R, Backhaus H. Variation of microbial communities in soil, rhizosphere, and rhizoplane in response to crop species, soil type, and crop development. Appl Environ Microbiol. 2001;67(12): 5849–5854.

35. Semagn K. Leaf tissue sampling and dna extraction protocols. Molecular plant taxonomy: methods and protocols. Totowa, NJ: Humana Press; 2014: 53–67.

36. White TJ, Bruns T, Lee S, Taylor J. Amplification and direct sequencing of fungal ribosomal RNA genes for phylogenetics. PCR protocols. San Diego: Academic Press; 1990: 315–22.

37. Madden T. The BLAST sequence analysis tool. NCBI Handb. 2013;2(5): 425–436.

38. Linkies A, Jacob S, Zink P, Maschemer M, Maier W, Koch E. Characterization of cultural traits and fungicidal activity of strains belonging to the fungal genus *Chaetomium*. J Appl Microbiol. 2021;131(1): 375–391.

39. Miller AN, Huhndorf SM. Multi-gene phylogenies indicate ascomal wall morphology is a better predictor of phylogenetic relationships than ascospore morphology in the Sordariales (Ascomycota, Fungi). Mol Phylogenet Evol. 2005;35(1): 60–75.

40. O’Donnell K, Cigelnik E. Two divergent intragenomic rDNA ITS2 types within a

monophyletic lineage of the fungus *Fusarium* are nonorthologous. Mol Phylogenet Evol. 1997;7(1): 103–116.

41. Gardes M, Bruns TD. ITS primers with enhanced specificity for basidiomycetes--application to the identification of mycorrhizae and rusts. Mol Ecol. 1993;2(2): 113–118.

42. Martin M. Cutadapt removes adapter sequences from high-throughput sequencing reads. EMBnet J. 2011;17(1): 10–12.

43. Afgan E, Baker D, Batut B, van den Beek M, Bouvier D, Cech M, et al. The Galaxy platform for accessible, reproducible and collaborative biomedical analyses: 2018 update. Nucleic Acids Res. 2018;46(W1): W537–W544.

44. R Core Team. R: A language and environment for statistical computing. R Foundation for Statistical Computing. 2022.

45. Callahan BJ, McMurdie PJ, Rosen MJ, Han AW, Johnson AJA, Holmes SP. DADA2: High-resolution sample inference from Illumina amplicon data. Nat Methods. 2016;13(7): 581–583.

46. Abarenkov K, Nilsson RH, Larsson KH, Taylor AFS, May TW, Frøslev TG, et al. The UNITE database for molecular identification and taxonomic communication of fungi and other eukaryotes: sequences, taxa and classifications reconsidered. Nucleic Acids Res. 2024;52(D1): D791–797.

47. Fernandez-Gnecco G, Gégu L, Covacevich F, Consolo VF, Bouffaud ML, Buscot F, et al. Alone as effective as together: AMF and *Trichoderma* inoculation boost maize performance but differentially shape soil and rhizosphere microbiota. J Sustain Agric Environ. 2024;3(1):1–16.

48. Wickham H, Averick M, Bryan J, Chang W, McGowan L, François R, et al. Welcome to the Tidyverse. J Open Source Softw. 2019;4(43): 1686.

49. Wickham H. ggplot2: Elegant graphics for data analysis. 2016;

50. Wilke CO, Wienik BM. _ggtext: Improved text rendering support for ’ggplot2’_. R package

version 0.1.2. 2022. Available from: https://wilkelab.org/ggtext/

51. Wickham H, François R, Henry L, Müller K. _dplyr: A grammar of data manipulation_. R package version 1.1.4. R Packag version. 2023;1(2).

52. Müller, K, Wickham H. _tibble: Simple data frames_. R package version 3.2.1. 2023; Available from: https://cran.r-project.org/package=tibble

53. Wickham H. _stringr: Simple, consistent wrappers for common string operations_. R package version 1.4.0. 2019.

54. Elkin LA, Kay M, Higgins JJ, Wobbrock JO. An aligned rank transform procedure for multifactor contrast tests. UIST 2021 - Proc 34th Annu ACM Symp User Interface Softw Technol. 2021;754–768.

55. Oksanen J, Simpson G, Blanchet F, Kindt R, Legendre P, Minchin P, et al. Vegan: community ecology package_. R package version 2.6-6.1. 2024.

56. McMurdie PJ, Holmes S. Phyloseq: An R Package for Reproducible Interactive Analysis and Graphics of Microbiome Census Data. PLoS One. 2013;8(4).

57. Schloss PD. Sequencing effort in amplicon sequence analyses. mSphere. 2024;0(0).

58. Lin H, Peddada S Das. Analysis of compositions of microbiomes with bias correction. Nat Commun. 2020;11(1): 3514.

59. VDLUFA. Phosphordüngung nach Bodenuntersuchung und Pflanzenbedarf. VDLUFA-Verlag Darmstadt; 2018.

60. Koch E, Ole Becker J, Berg G, Hauschild R, Jehle J, Köhl J, et al. Biocontrol of plant diseases is not an unsafe technology! J Plant Dis Prot. 2018;125: 121–125.

61. Pandey AK, Kumar A, Dinesh K, Varshney R, Dutta P. The hunt for beneficial fungi for tomato crop improvement – Advantages and perspectives. Plant Stress. 2022;6: 100-110.

62. Schisler DA, Slininger PJ. Microbial selection strategies that enhance the likelihood of

developing commercial biological control products. J Ind Microbiol Biotechnol. 1997;19(3): 172–179.

63. Philippot L, Raaijmakers JM, Lemanceau P, van der Putten WH. Going back to the roots: the microbial ecology of the rhizosphere. Nat Rev Microbiol. 2013;11(11): 789–799.

64. Bhagat S, Shete P, Jain A. Plant health: Feedback effect of root exudates and rhizobiome interactions. Rhizobiome Ecol Manag Appl. 2023;345–375.

65. Vorholt JA. Microbial life in the phyllosphere. Nat Rev Microbiol. 2012;10(12): 828–840.

66. Rastogi G, Coaker GL, Leveau JHJ. New insights into the structure and function of phyllosphere microbiota through high-throughput molecular approaches. FEMS Microbiol Lett. 2013;348(1): 1–10.

67. Fürnkranz M, Wanek W, Richter A, Abell G, Rasche F, Sessitsch A. Nitrogen fixation by phyllosphere bacteria associated with higher plants and their colonizing epiphytes of a tropical lowland rainforest of Costa Rica. ISME J. 2008;2(5): 561–570.

68. Lalaymia I, Naveau F, Arguelles Arias A, Ongena M, Picaud T, Declerck S, et al. Screening and efficacy evaluation of antagonistic fungi against *Phytophthora infestans* and combination with arbuscular mycorrhizal fungi for biocontrol of late blight in potato. Front Agron. 2022;4: 1–20.

69. Pitt JI. *Penicillium* | *Penicillium* and *Talaromyces*: Introduction. Encyclopedia of Food Microbiology. Second Edi. Oxford: Academic Press; 2014. 6–13.

70. Khan R, Anwar F, Ghazali FM. A comprehensive review of mycotoxins: Toxicology, detection, and effective mitigation approaches. Heliyon. 2024;10(8).

71. Harman GE, Howell CR, Viterbo A, Chet I, Lorito M. *Trichoderma* species-opportunistic, avirulent plant symbionts. Nat Rev Microbiol. 2004;2(1): 43–56.

72. Adnan M, Islam W, Shabbir A, Khan KA, Ghramh HA, Huang Z, et al. Plant defense against fungal pathogens by antagonistic fungi with *Trichoderma* in focus. Microb Pathog. 2019;129:

7–18.

73. Funck Jensen D, Dubey M, Jensen B, Karlsson M. *Clonostachys rosea* to control plant diseases. 2021; 429–72.

74. Egel DS, Hoagland L, Davis J, Marchino C, Bloomquist M. Efficacy of organic disease control products on common foliar diseases of tomato in field and greenhouse trials. Crop Prot. 2019;122: 90–97.

75. Høyer AK, Jørgensen HJL, Amby DB, Jensen B. Endophytic colonization of tomato plants by the biological control agent *Clonostachys rosea*. Biol Integr Control plant Pathog IOBC-WPRS Bull. 2016;115: 101–106.

76. European Food SafetyAuthority. Conclusion on the peer review of the pesticide risk assessment of the active substance *Trichoderma gamsii* ICC080. EFSA J. 2015;13(4).

77. Arena M, Auteri D, Barmaz S, Bellisai G, Brancato A, Brocca D, et al. Peer review of the pesticide risk assessment of the active substance *Clonostachys rosea* strain J1446 (approved in Regulation (EU) No 540/2011 as *Gliocladium catenulatum* strain J1446). EFSA J. 2017;15(7).

78. Manzotti A, Bergna A, Burow M, Jørgensen HJL, Cernava T, Berg G, et al. Insights into the community structure and lifestyle of the fungal root endophytes of tomato by combining amplicon sequencing and isolation approaches with phytohormone profiling. FEMS Microbiol Ecol. 2020;96(5).

79. Ibrahim SRM, Mohamed SA, Sindi I, Mohamed GA. Biologically active secondary metabolites and biotechnological applications of species of the family Chaetomiaceae (Sordariales): an updated review from 2016 to 2021. Mycol Prog. 2021;20: 595–639.

80. Kedves O, Kocsubé S, Bata T, Andersson MA, Salo JM, Mikkola R, et al. *Chaetomium* and *Chaetomium*-like species from european indoor environments include D*ichotomopilus finlandicus* sp. Nov. Pathogens. 2021;10(9).

81. Liu C, Kan S, Zhou H, Zhang H, Chang Z. Mycolytic effect and underlying mechanism of *Chaetomium subaffine* LB-1 on phytopathogenic fungi via morphological, physio-biochemical and transcriptomic analysis. J Phytopathol. 2024;172(1): 1–12.

82. Stephan D, Schmitt A, Martins Carvalho S, Seddon B, Koch E. Evaluation of biocontrol preparations and plant extracts for the control of *Phytophthora infestans* on potato leaves. Eur J Plant Pathol. 2005;112(3): 235–46.

83. Berg G, Rybakova D, Fischer D, Cernava T, Vergès MCC, Charles T, et al. Microbiome definition re-visited: old concepts and new challenges. Vol. 8, Microbiome. England; 2020. 103.

84. Gruet C, Muller D, Moënne-Loccoz Y. Significance of the diversification of wheat species for the assembly and functioning of the root-associated microbiome. Front Microbiol. 2022;12.

85. Sahu KP, Patel A, Kumar M, Sheoran N, Mehta S, Reddy B, et al. Integrated metabarcoding and culturomic-based microbiome profiling of rice phyllosphere reveal diverse and functional bacterial communities for blast disease suppression. Front Microbiol. 2021;12 :1–21.

86. Watson A, Napier T, Mazwan Muhammad R, Rozana Nik Mohd Masdek N, Zahrah Ponari S, Anak Makup J, et al. Diseases of cucurbit vegetables. Prime Fact 832. 2009;2–3.

87. Ogórek R, Lejman A, Pusz W, Miłuch A, Miodyńska P. Characteristics and taxonomy of *Cladosporium* fungi. Mikol Lek. 2012;19(2): 80–85.

88. Hamayun M, Afzal Khan S, Ahmad N, Tang DS, Kang SM, Na CI, et al. *Cladosporium sphaerospermum* as a new plant growth-promoting endophyte from the roots of *Glycine max* (L.) Merr. World J Microbiol Biotechnol. 2009;25: 627–632.

89. Sandoval-Denis M, Sutton DA, Martin-Vicente A, Cano-Lira JF, Wiederhold N, Guarro J, et al. *Cladosporium* species recovered from clinical samples in the United States. J Clin Microbiol. 2015;53(9): 2990–3000.

90. Shakya M, Gottel N, Castro H, Yang ZK, Gunter L, Labbé J, et al. A multifactor analysis of

fungal and bacterial community structure in the root microbiome of mature populus deltoides trees. PLoS One. 2013;8(10).

91. Pfeiffer S, Mitter B, Oswald A, Schloter-Hai B, Schloter M, Declerck S, et al. Rhizosphere microbiomes of potato cultivated in the high Andes show stable and dynamic core microbiomes with different responses to plant development. FEMS Microbiol Ecol. 2016;93(2)

92. Taffner J, Cernava T, Erlacher A, Berg G. Novel insights into plant-associated archaea and their functioning in arugula (*Eruca sativa* Mill.). J Adv Res. 2019;19: 39–48.

93. Lemanceau P, Blouin M, Muller D, Moënne-Loccoz Y. Let the core microbiota be functional. Trends Plant Sci. 2017;22(7): 583–595.

94. Pascale A, Proietti S, Pantelides IS, Stringlis IA. Modulation of the root microbiome by plant molecules: the basis for targeted disease suppression and plant growth promotion. Front Plant Sci. 2020;10:1–23.

95. Berg G, Köberl M, Rybakova D, Müller H, Grosch R, Smalla K. Plant microbial diversity is suggested as the key to future biocontrol and health trends. FEMS Microbiol Ecol. 2017;93(5).

96. Lindow SE, Brandl MT. Microbiology of the phyllosphere. Appl Environ Microbiol. 2003;69(4): 1875–1883.

97. Ramudingana P, Mamphogoro TP, Kamutando CN, Maboko MM, Modika KY, Moloto KW, et al. Antagonistic potential of endophytic fungal isolates of tomato (*Solanum lycopersicum* L.) fruits against post-harvest disease-causing pathogens of tomatoes: An *in vitro* investigation. Fungal Biol. 2024;128(4): 1847–1858.

98. El-Hasan A, Ngatia G, Link TI, Voegele RT. Isolation, identification, and biocontrol potential of root fungal endophytes associated with solanaceous plants against potato late blight

(*Phytophthora infestans*). Plants. 2022;11(12).

99. Peixoto RS, Voolstra CR, Sweet M, Duarte CM, Carvalho S, Villela H, et al. Harnessing the microbiome to prevent global biodiversity loss. Nat Microbiol. 2022;7(11): 1726–1735.

100. Larousse M, Rancurel C, Syska C, Palero F, Etienne C, Industri B, et al. Tomato root microbiota and *Phytophthora parasitica*-associated disease. Microbiome. 2017;5(1): 1–11.

101. Berg G, Smalla K. Plant species and soil type cooperatively shape the structure and function of microbial communities in the rhizosphere. FEMS Microbiol Ecol. 2009;68(1): 1–13.

102. Docherty KM, Borton HM, Espinosa N, Gebhardt M, Gil-Loaiza J, Gutknecht JLM, et al. Key edaphic properties largely explain temporal and geographic variation in soil microbial communities across four biomes. PLoS One. 2015;10(11): 1–23.

103. Zhang T, Wang NF, Liu HY, Zhang YQ, Yu LY. Soil pH is a key determinant of soil fungal community composition in the Ny-Ålesund Region, Svalbard (High Arctic). Front Microbiol. 2016;7: 1–10.

104. Bardgett RD, Van Der Putten WH. Belowground biodiversity and ecosystem functioning. Nature. 2014;515(7528): 505–511.

Supporting information

S1 Table. Non-parametric aligned-rank ANOVA results for species richness using the ARTool package (v.0.11.1).

S2 Table. Non-parametric aligned-rank ANOVA results for Shannon using the ARTool package (v.0.11.1).

S3 Table. Non-parametric aligned-rank ANOVA results for species evenness index using the ARTool package (v.0.11.1).

S4 Table. Non-parametric aligned-rank ANOVA results for the Simpson index using the ARTool package (v.0.11.1).

S5 Table. Permutation test results from Adonis2 for beta diversity analysis with Bray-Curtis dissimilarity.

S6 Table. ANCOM-BC2 results of differential abundance of fungal ASVs between soil A and soil B in rhizosphere samples based on Log Fold Change (LogFC) and structural zero analysis.

S7 Table. ANCOM-BC2 results of differential abundance of fungal ASVs between soil A and soil B in phyllosphere samples based on Log Fold Change (LogFC) and structural zero analysis.

S8 Table. ANCOM-BC2 results of differential abundance of fungal ASVs between soil A and soil B in endosphere samples based on Log Fold Change (LogFC) and structural zero analysis.

S9 Table. Total collection of fungal isolates inhibiting *P. infestans, in vitro*.

S10 Table. Collection of 61 identified fungal biocontrol candidates inhibiting *P. infestans* after eliminating potential plant and human pathogens based on literature search.

S11 Table. Collection of 165 unidentified fungal isolates after eliminating those growing at 37°C and non viable isolates.

S12 Table. Collection of 246 initial fungal isolates recovered from tomato plant tissues.

S1 Fig. Rarefaction curve of observed species richness after sequencing of ITS rRNA gene from the rhizosphere, endosphere, and phyllosphere samples of tomato plants grown in soil origins A and B.

Figure 1

Click here to access/download;Figure;Fig 1.tif

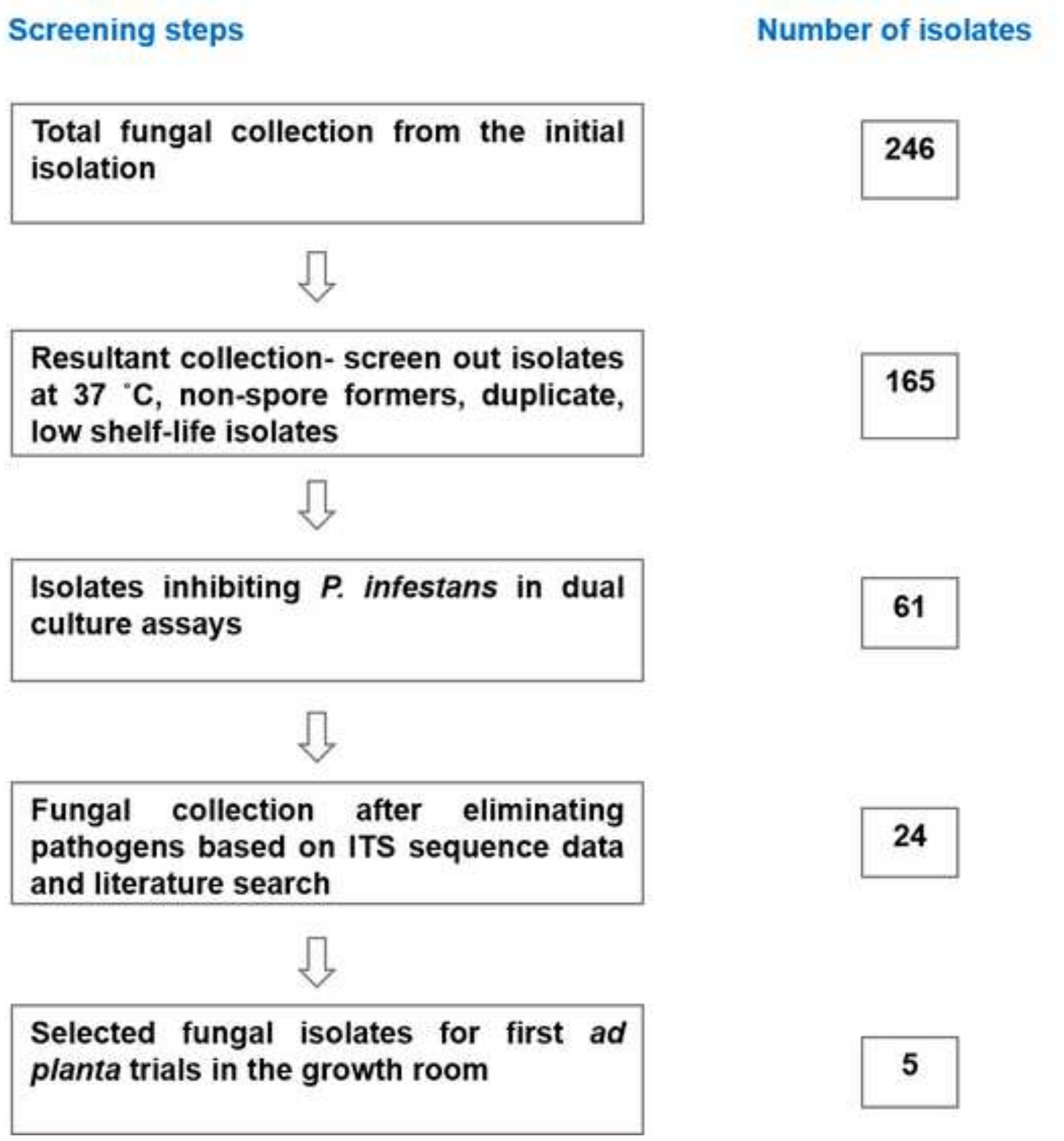

Figure 2

Click here to access/download;Figure;Fig 2.tif

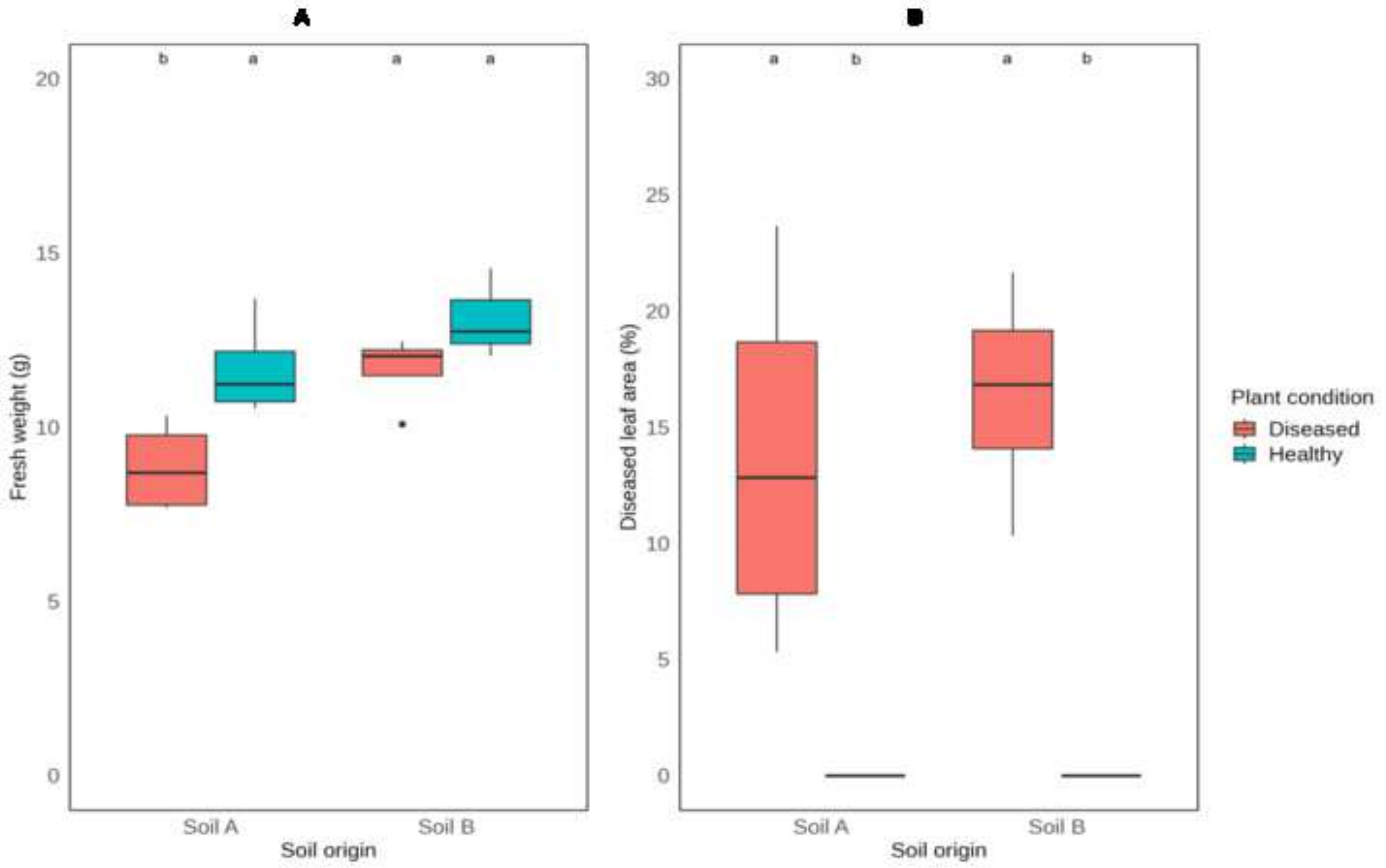

Figure 3

Click here to access/download;Figure;Fig 3.tif

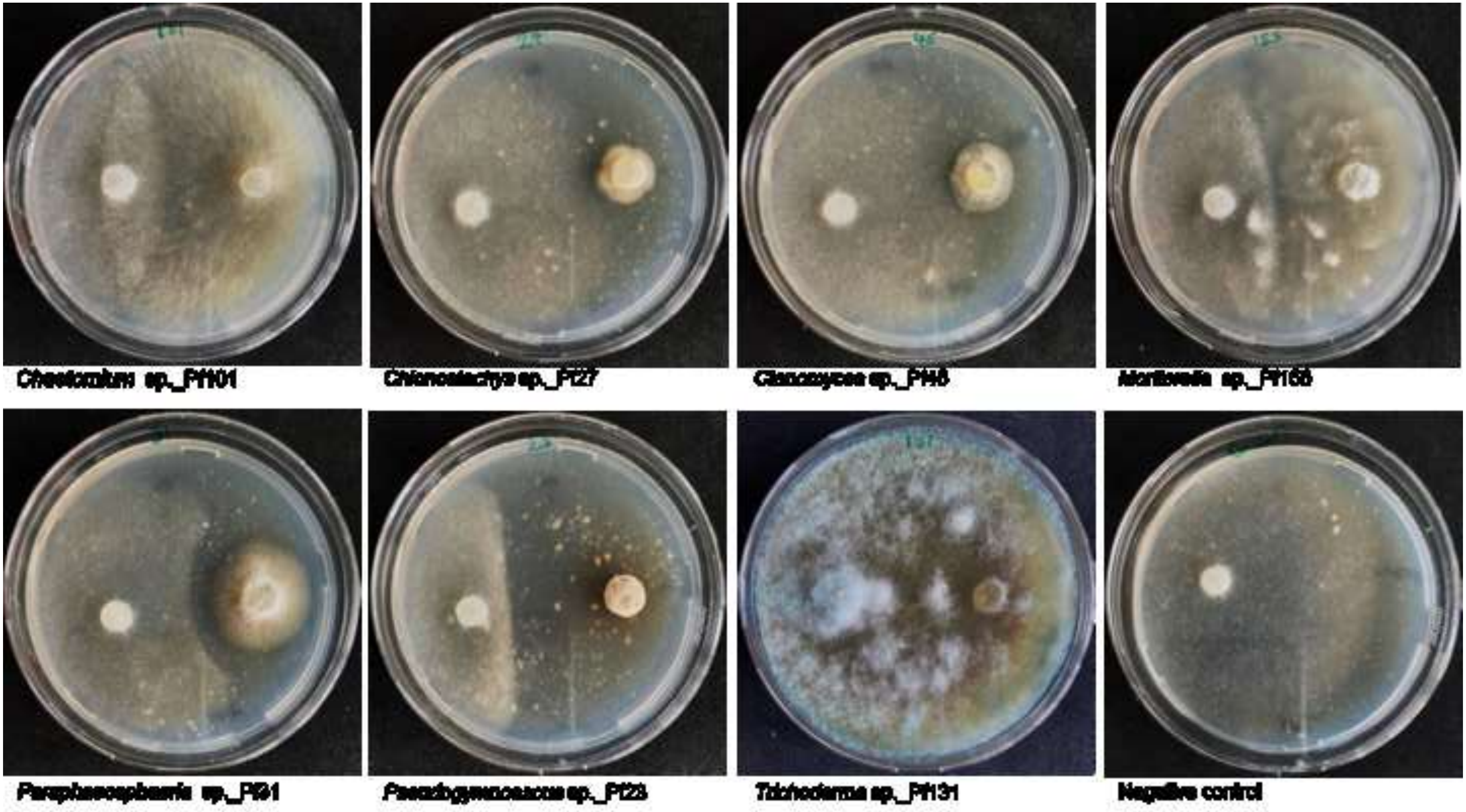

Figure 4

Click here to access/download;Figure;Fig 4.tif

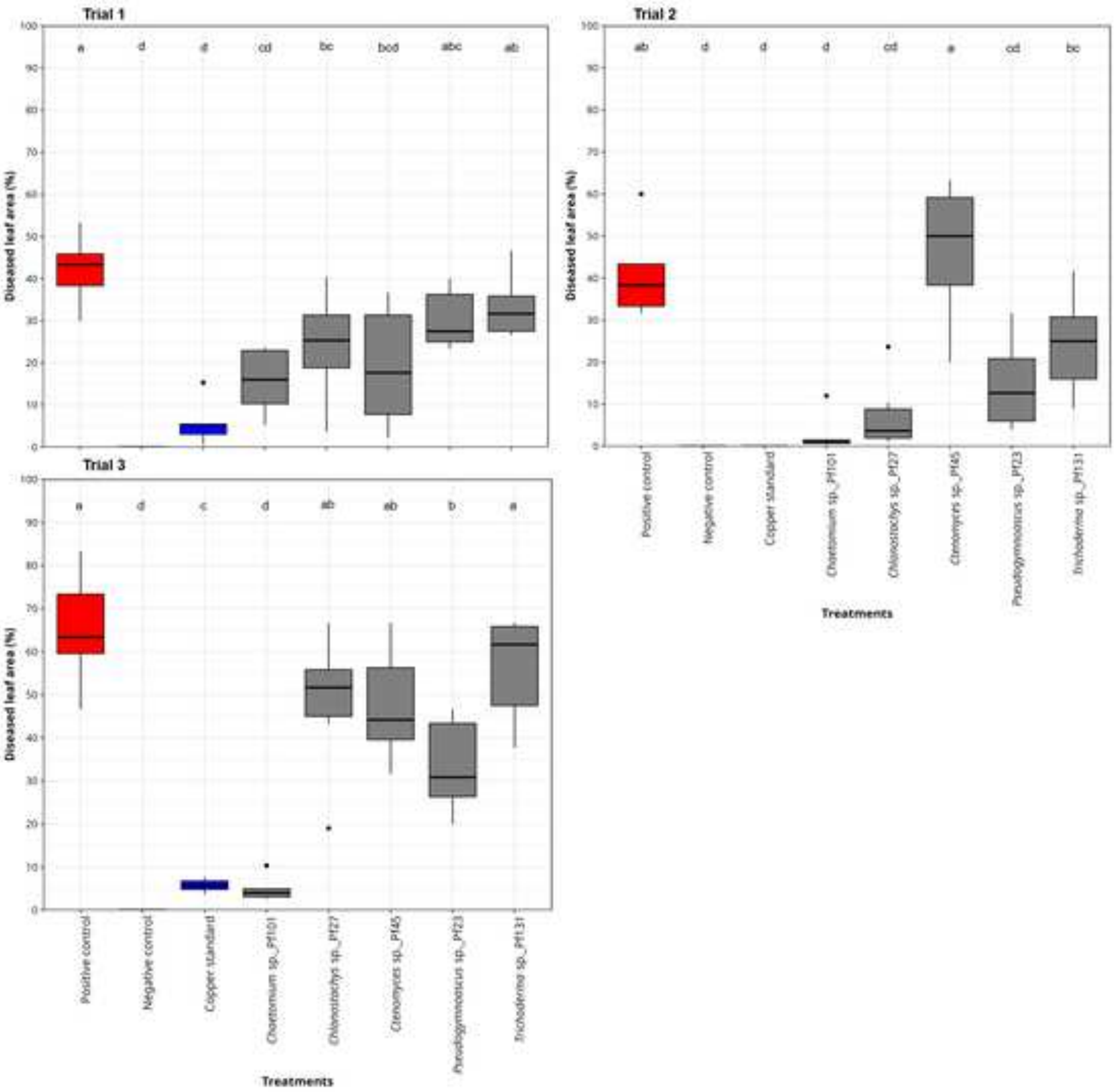

Figure 5

Click here to access/download;Figure;Fig 5.tif

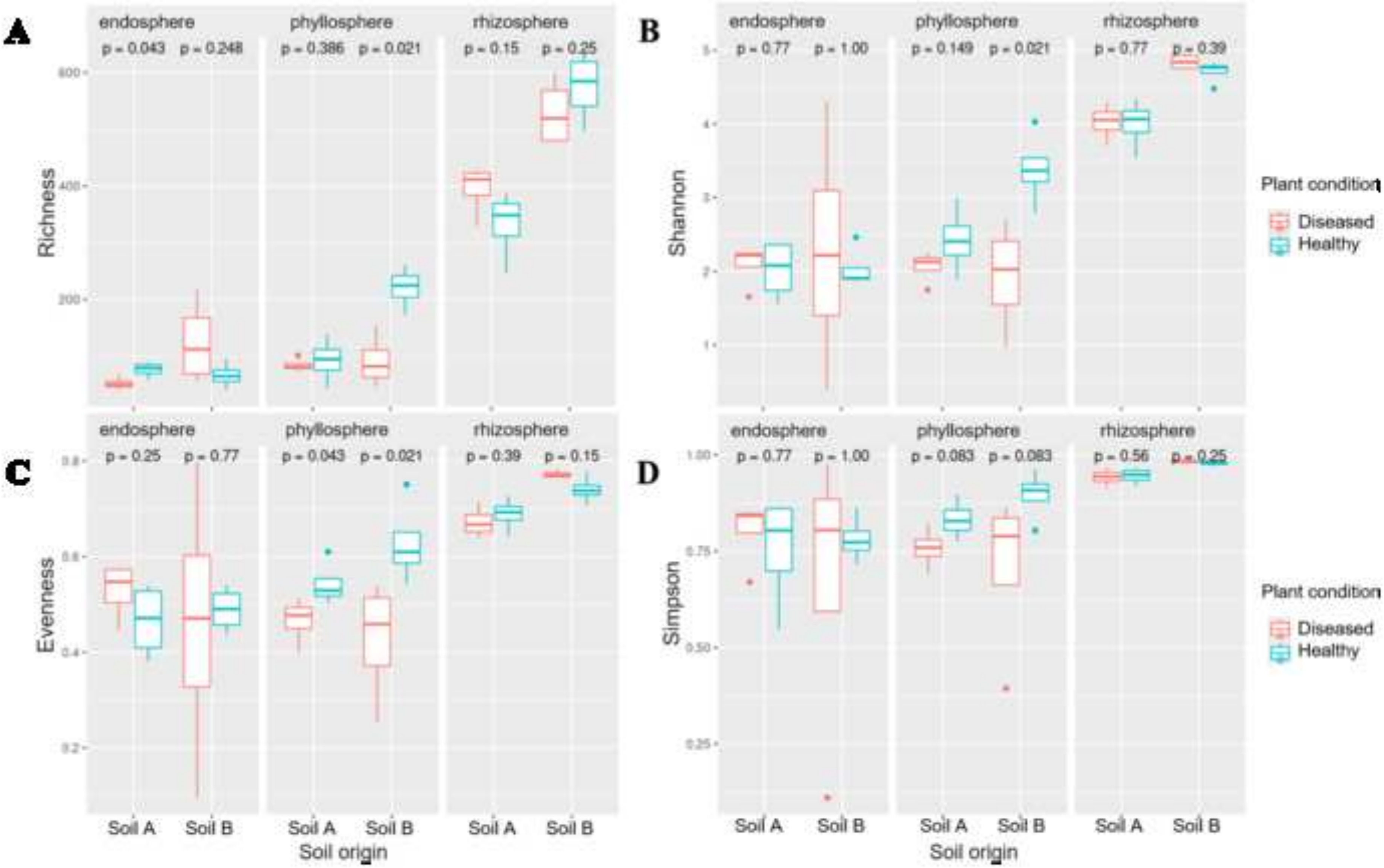

Figure 6

Click here to access/download;Figure;Fig 6.tif

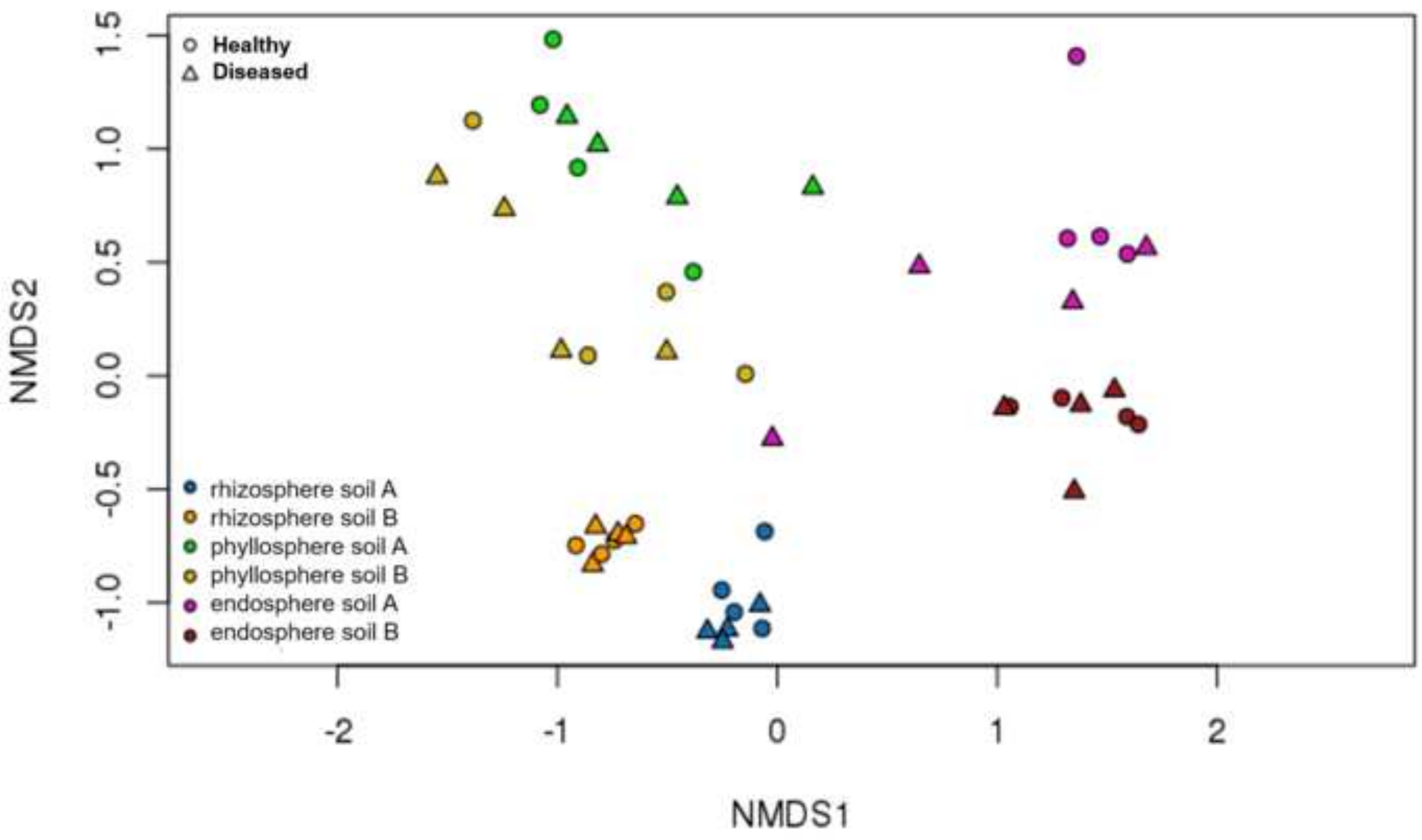

Figure 7

Click here to access/download;Figure;Fig 7.tif

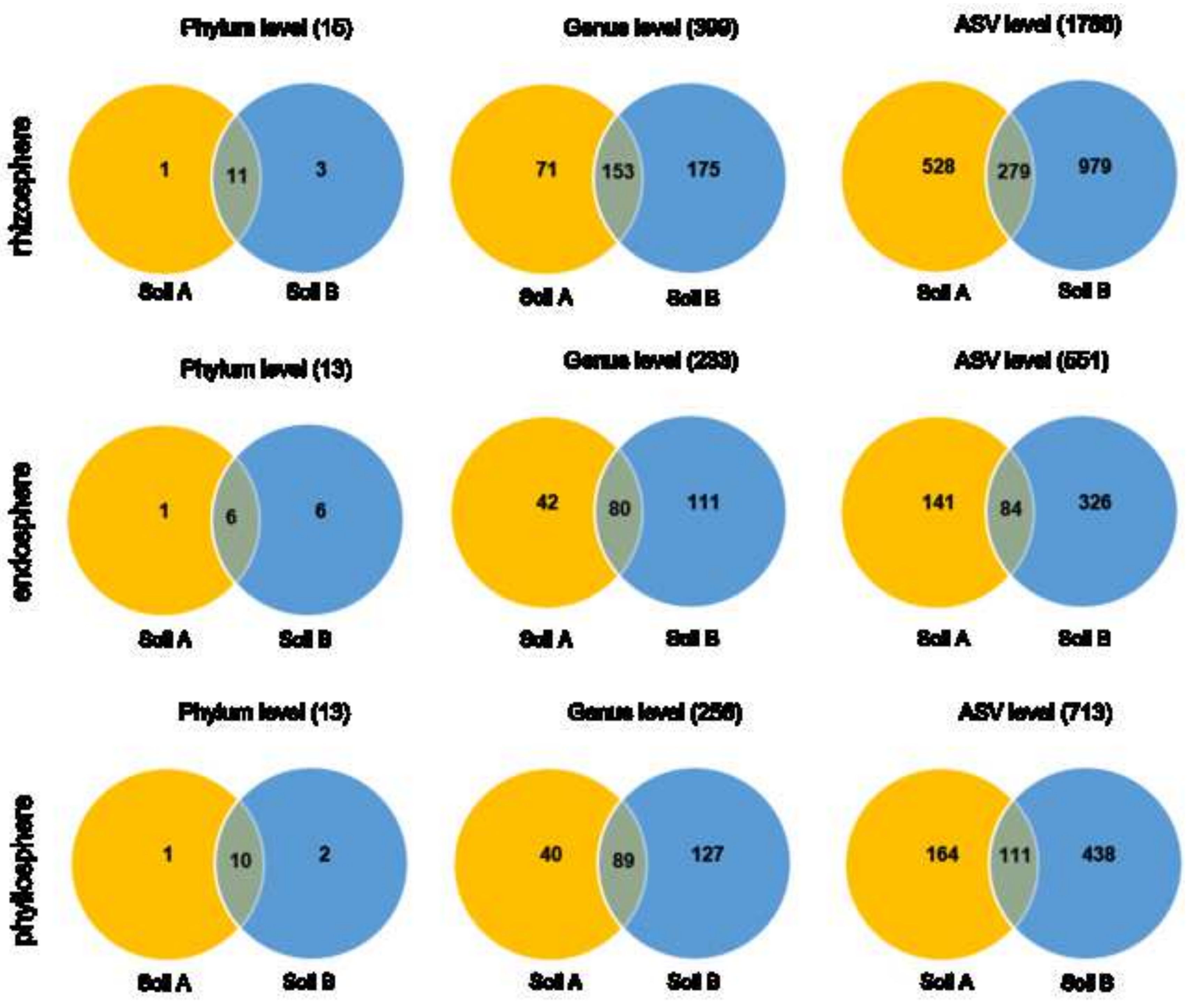

Figure 8

Click here to access/download;Figure;Fig 8.tif

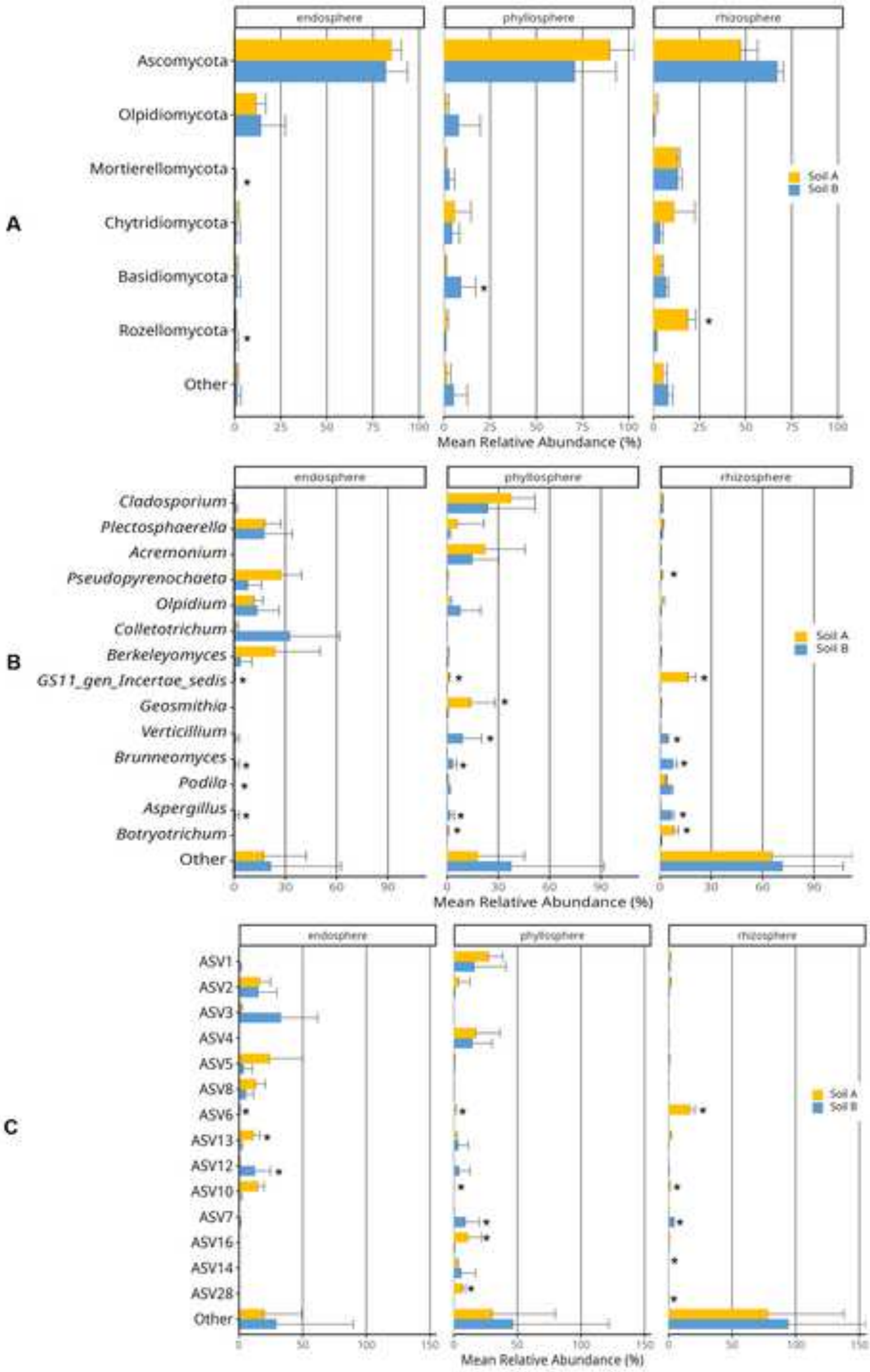

S11 Table

Click here to access/download
Supporting Information
S11 Table.xlsx

S12 Table

Click here to access/download
Supporting Information
S12 Table.xlsx

Click here to access/download
Supporting Information
Supplementary material_revised.docx

Chaetomium, *Chlonostachys*, and *Pseudogymnoascus* isolates from tomato tissues significantly suppress *Phytophthora infestans* in tomato

Philemon Orwa[1], Theresa Kuhl-Nagel[2], Rosa Meinhold-Ernst[1], Arne Seyer[1,4], Johannes A. Jehle[1], Romano Mwirichia[3], Ada Linkies[1*]

[1] Julius Kühn Institute (JKI) - Federal Research Centre for Cultivated Plants, Institute for Biological Control, 69221 Dossenheim, Germany

[2] Leibniz Institute of Vegetable and Ornamental Crops (IGZ), Plant-Microbe Systems, Großbeeren, Germany

[3] University of Embu, Department of Biological Sciences, 6 60100 Embu, Kenya

[4] Geisenheim University, Department of Crop Protection, 65366 Geisenheim, Germany

* Corresponding author:

ada.linkies@julius-kuehn.de

Short title: Fungal isolates significantly suppress *P. infestans* in tomato

Abstract

Late blight is a disease whose causative agent is the oomycete *Phytophthora infestans*. It is one of the most destructive pathogenic oomycetes and a major challenge to global tomato production. The pathogen is difficult to manage because of its ability to evolve thereby evading host resistance. The

aim of this study was to screen for potential antagonists of *P. infestans* using a combination of culture and microbiome-based approaches. Samples were collected from healthy and *P. infestans*-infected tomato plants grown in soil collected from two organic tomato growers in the Rhine-Main area in Germany. A total of 246 fungal isolates were screened for their antagonistic activity against *P. infestans*. Most of the isolates that exhibited *in vitro* antagonistic activity were from the genera *Penicillium, Trichoderma, Chlonostachys, Mortierella,* and *Pseudogymnoascus*. Following a stepwise *in vitro* screening strategy that accounted for growth features, ecological aspects, taxonomic data, potential health risks, commercial properties, and antagonistic efficacy, five fungal isolates were eventually selected for plant trials. *Chaetomium subaffine* showed the highest inhibitory effect against *P. infestans* across three trials whereby the percentage of diseased leaf area reduced by 90% compared to the control. *Chlonostachys* and *Pseudogymnoascus* spp. were effective in two trials, while *Trichoderma* and *Ctenomyces* spp. showed weak disease suppressive effects. In parallel, we characterized the fungal microbiome of the rhizosphere, phyllosphere, and endosphere from healthy and diseased tomato plants using ITS-rRNA sequencing. The fungal community differed significantly between the two soil origins, but *P. infestans* did not significantly influence fungal microbiota composition. Notably, 70% of our antagonistic fungi from the culture collection were detected in the tomato microbiome. This work identified isolates of *Chaetomium subaffine, Clonostachys sp.,* and *Pseudogymnoascus sp.* as potential biocontrol candidates promoting plant health. The findings highlight the importance of combined functional screening and microbiome profiling for identifying fungal antagonists.

Keywords: fungal microbiome, ITS rDNA, Illumina sequencing, tomato rhizosphere, microbial antagonists, biological control.

Introduction

Tomato is an important crop forming a vital component of the global diet, owing to its nutritional and health benefits [1]. In addition to other fungal pathogens such as *Alternaria solani*, *Botrytis cinerea*, and *Fusarium oxysporum* f. sp. *lycopersici* [2], late blight disease caused by *Phytophthora infestans*, is among the most devastating pathogenic oomycetes threatening global tomato production [3]. Studies indicate that *P. infestans* has become more aggressive over the past decades [4,5] and that the rapidly evolving genotypes are linked to severe epidemic global outbreaks [6]. Management costs and yield loss associated with late blight disease ranges between 3-10 billion United States Dollars per year globally [7,8].

Conventional approaches for managing *P. infestans* primarily utilize synthetic fungicides, which adversely impact the environment and may harm terrestrial and aquatic organisms [9]. The pathogen also poses the risk of fungicide resistance as it evolves rapidly [10]. In this regard, there are increasingly stringent regulations regarding the use of chemical-synthetic and copper fungicides. The European Union (EU) for instance, has heightened restrictions on the registration of chemical-synthetic pesticides through directives 2009/128/EC and (EU) 2019/782, aiming at lowering the risks associated with pesticide application on humans and the environment [11]. Therefore, there is an urgent need for more eco-friendly alternatives to chemical-synthetic and copper fungicides. Microbial antagonists promote plant growth and suppress plant pathogens through distinct mechanisms, including induced systemic resistance (ISR) [12], competition for space and nutrients [13], or the production of antimicrobial secondary metabolites [14]. The genera *Bacillus* and *Pseudomonas* are commonly found as microbial antagonists [15,16], while *Trichoderma* is among the most investigated and described fungal genera used as a biocontrol agent [17,18].

The soil microbiome includes many beneficial microorganisms and is an important source for plants to recruit their rhizosphere microbiome [19]. Soil microbiomes provide unique properties for plant

pathogen control, also known as soil-borne legacy, which is beneficial to plants of the next generation thriving on the same soil [20]. However, plant pathogens can drive dysbiosis of the plant microbiome with consequences for plant health [21]. The rhizosphere, shaped by plant roots, is a complex habitat for diverse microorganisms, such as protozoa, mycorrhizal fungi, nitrogen-fixing bacteria, plant growth-promoting rhizobacteria (PGPR), and mycoparasitic fungi [22–25]. Fungi play multifaceted roles in the soil, including nitrogen fixation, and synthesis of plant growth–promoting hormones, they are natural biological agents against root pathogens and promoters of plant resilience under drought stress [26–28]. Additionally, fungi are key players in stabilizing soil organic matter by breaking down plant residues [29]. In recent years, there has been a growing emphasis on harnessing the diversity of soil fungi as a sustainable method to improve the quality of soil and boost crop productivity —an emerging strategy holding significant promise for the future of agriculture [30]. Several fungal species have been described with biocontrol potential against *P. infesta*ns [31]. Research on new or better biocontrol agents often focuses mainly on *in vitro* confrontation assay results as the basis for the selection of promising candidates, a majority of which do not meet the requirements for commercial use. The current work seeks to address the above-described research limitations by finding answers to the following questions. (i) Are the micro-compartments of tomato plants a suitable source for isolating fungal strains with effective biocontrol potential against *P. infestans*, which also meet selection criteria for later product development and registration? (ii) Does the microbiome structure differ between healthy and *P. infestans*-infected tomato plants grown in two soil origins, and can specific fungal groups be identified as key contributors to disease suppression? Therefore, we applied an integrated approach in the search for fungal antagonists against *P. infestans* by linking culture-dependent characterization to microbiome analyses and assessing the results in the context of tomato plant health. In the culture-dependent approach, we isolated and characterized potential fungal antagonists from diseased and healthy tomato plant samples in a stepwise selection procedure previously described by Köhl et al. [32] to select promising candidates for *ad planta* trials against *P. infestans*. The selection

procedure incorporates commercial aspects, including growth on low-cost growth media, massive spore production, and evaluating potential health risks, in addition to antagonistic efficacy. In the microbiome approach, we characterized and compared the fungal microbiome of the phyllosphere, the endosphere, and the rhizosphere of diseased and healthy tomato plants grown in soils from different origins using high-throughput sequencing (HTS). Linking culture-dependent work to the microbiome provides deeper insights to characterize beneficial fungi for biocontrol of tomato late blight and other tomato diseases.

Methods

Soil sampling, tomato plant growth conditions, and inoculation with *P. infestans*

Tomato plants (*Solanum lycopersicum* `Red Robin´ cultivar) were grown in two different natural soils that were previously used for organic ~~open soil~~ tomato cultivation in the greenhouse. No chemical-synthetic pesticides and fertilizers were applied previously and during the experiments. As in both soils tomato plants were grown in the previous growing season, ~~the assumption was that~~ these soils are enriched with organisms that are supportive of tomato plant health. The soils were collected from two different professional tomato growers in the Rhine-Main area at Domäne Mechthildshausen, Wiesbaden, 50°02′ N, 8°19′ E (soil A), and Solidarische Landwirtschaft Rüsselsheim, 49°58′ N, 8°25′ E (soil B) in February 2022. The contact to the growers and the corresponding field sites (soil A and B) was provided by Christian Fetzer, employee from the Landesbetrieb Landwirtschaft Hessen, a governmental educational and advisory institution of the State of Hesse, subordinate to the Hessian Ministry of Agriculture and Environment, Viticulture, Forestry, Hunting, and Home Affairs (HMLU). The growers gave permission to collect soil from their greenhouses for this study. Soil samples were

Formatted: Font color: Auto

taken at five positions throughout the greenhouse area with a depth of 20 cm and a mixed sample from all samples was used as substrate for the experiments. Eight tomato seeds were sown in soils from both locations (soil A and B) in each pot (size 8 x 8 x 8.5 cm) within 24 h after soil collection. Four seedlings for each treatment and soil origin were transferred to individual pots 14 days after sowing. Plants were grown at a temperature range of 19-22 °C for subsequent inoculation with *P. infestans* (isolate 606 of JKI culture collection) as described in Drenker et al. [33]. Briefly, four 5-weeks-old plants each grown in soil A and soil B were spray inoculated with 7 ml of 2 x 10^4 ml^{-1} ~~sporangia~~ zoospores per plant. Four plants grown in each soil were not inoculated and served as healthy controls. Scoring for disease symptoms (percentage symptomatic area) was performed 2 weeks after inoculation with *P. infestans*.

Formatted: Font: Not Italic

Sample preparation for microbiome analysis and isolation of fungal antagonists

Samples for partial ITS amplicon sequencing and isolation of viable fungi were collected from the above-described tomato plants. Samples from leaves, roots, and rhizosphere soil were collected from the tomato plant. One plant was considered one replicate, in total four replicate plants per treatment were analyzed. Several plant tissues per micro-compartment were pooled as described in detail below. Plant samples were processed for ITS amplicon sequencing and isolation of fungal antagonists following the protocol of Wieland et al. [34], with some modifications, described as follows.

Rhizosphere sample collection

The roots of each plant were shaken carefully to remove non-adhering soil. Five grams of soil adhering to the roots were transferred in sterile 50 mL centrifuge tubes containing 20 mL of sterile saline buffer (0.85% NaCl) and mixed vigorously by vortexing for 2 min. The roots (approximately 5 g), from which the rhizospheric soil had been dislodged were soaked in 20 mL of sterile saline buffer (0.85% NaCl)

and mixed thoroughly by vortexing for 5 min to obtain the rhizoplane. Both solutions were combined to obtain the final rhizosphere sample. A total of 16 rhizosphere samples were obtained for microbiome analysis, four replicates from each soil origin, both from healthy and diseased plants. The remaining rhizosphere material from the four replicate plants was pooled for the isolation of fungal antagonists. A total of four rhizosphere samples were obtained for fungal isolation, one replicate from each soil origin, and from healthy and diseased plants.

Endosphere sample collection

Roots (approximately 5 g) from rhizoplane extraction described above were sterilized with ethanol (75%; 2 min), sodium hypochlorite solution (50%; 2 min), and ethanol (75%; 1 min) and rinsed five times in sterile distilled water. Root samples (100–150 mg) were then homogenized with a sterile pestle and mortar in 20 mL of sterile saline buffer (0.85% NaCl) to obtain the final endosphere sample. In total, 16 endosphere samples were obtained for microbiome analysis, four replicates from each soil origin, and healthy and diseased plants. The remaining endosphere material from the four replicate plants was pooled for the isolation of fungal antagonists. A total of four endosphere samples were obtained for fungal isolation, one replicate from each soil origin, and from healthy and diseased plants. A sterility check was performed by placing a subset of the sterilized roots on PDA at 27 °C for 7 days, and the absence of microbial growth ensured the sterility of the root surfaces.

Phyllosphere sample collection

Samples for the microbiome analysis and isolation of leaf-associated fungi followed the protocol by Kassa Semagn [35]: Fresh leaf samples (100–150 mg) were harvested from each plant by pooling 4 portions obtained from different branches, cut into smaller pieces into the collection tubes and processed immediately. Notably, samples from plants infected with *P. infestans* were collected from

leaf patches with healthy-looking samples adjacent to the diseased area. A total of 16 rhizosphere samples were obtained for microbiome analysis, four replicates from each soil origin, from both healthy and diseased plants. The remaining phyllosphere material from the four replicate plants was pooled for the isolation of fungal antagonists. A total of four phyllosphere samples were obtained for fungal isolation, one replicate from each soil origin, and from healthy and diseased plants.

Collection of fungal isolates

Culturable fungi were isolated from the phyllosphere and rhizosphere of healthy and diseased plants grown in soil A and B. A total of eight samples were collected for fungal isolation, including four rhizosphere samples (as described above) and four phyllosphere samples as described above. Precisely, 100 µl of serially diluted samples (10^{-1} - 10^{-3}) in sterile saline buffer (0.85% NaCl) were spread-plated onto potato dextrose agar (PDA) and oatmeal agar (OMA) supplemented with antibiotics (rifampicin (20 mg/ml) and streptomycin (50 mg/ml)). Plates were incubated at different temperatures (14, 21, and 28 °C) to increase the diversity of the collection. The first assessment of fungal growth was conducted 7 days after plating and was based on differences in growth morphology, whereas the last assessment of fungal growth was conducted 14 days after plating. Fungal colonies that emerged on plates with different media, at varying temperatures, and from different tissue samples were isolated through a series of sub-culturing steps.

Characterization of fungal isolates regarding their biocontrol potential

The present work adopted a stepwise screening approach developed by Köhl et al. [32] to select fungal candidates with promising characteristics as microbial antagonists of tomato pathogens (Fig 1). The isolated 246 fungi were examined under the microscope for their ability to form spores as this is a key parameter in the mass production of fungi for commercial purposes (S12 Table). The fungal isolates

were further incubated ~~All isolates that were able to grow~~ at 37 °C and those that grew in this temperature were eliminated as they could present potential risks to majorly humans and other mammals (S12 Table). ~~The remaining isolates were screened for abundant spore production as this is a key parameter in the mass production of fungi for commercial purposes.~~ Additionally, morphologically identical isolates that appeared repeatedly and those that lost viability were eliminated. The remaining 165 fungal ~~collection~~ isolates ~~was~~ were subjected to dual-culture assays with *P. infestans* as the target plant pathogen in this work (S11 Table). A total of 61 f~~F~~ungal isolates that either formed inhibition zones when co-cultured with the pathogen on Petri dish or overgrew the pathogen were deemed to exhibit ~~ed~~ antagonistic effects, through antibiosis and matrix completion, respectively (S9 Table). The fungal antagonists were identified as potential biocontrol candidates and were ~~against *P. infestans* based on the formation of inhibition zones were~~ ~~characterized~~ molecularly characterized by sequencing the ~~using~~ ITS locus, yielding an amplicon of approximately 560bp ~~sequencing~~(S9 Table). The already identified antagonists were further screened against *A. solani* (isolate 62028 of JKI culture collection) to reveal their broader antagonistic effects (S9 Table). Notably, the *in vitro* confrontation assays with *A. solani* only served as criteria for selecting the most promising antagonists, therefore, the pathogen was not used in the subsequent experiments. A literature search was performed and species that have previously been described as plant, human, or animal pathogens were eliminated, particularly *Penicillium* species which formed a higher proportion of our collection. The resultant collection of 24 fungal isolates ~~was retained~~were re-screened for *in -vitro* inhibitor~~r~~y activity on *P. infestans* but this time on rye agar, which is the most suitable growth media for the pathogen (S10 Table). In the end,~~f~~ 5 fungal antagonistic candidates were selected for~~or~~ *ad planta* tests with *P. infestans* on tomato plants (Table 1). The criteria for selection of the 5 potential fungal antagonists included (i) literature evidence as a potential plant biocontrol agent: both broadly described genera, including *Trichoderma* and scarcely described genera, including *Pseudogymnoascus* were

Formatted: Font: Italic

Formatted: Font: Not Italic

considered; (ii) ability to produce at least 4 x 10^5 ml^{-1} spores on standard fungal media (PDA) within 21 days of incubation at 25 °C; (iii) no negative effects on healthy tomato plants following leaf application.

Fig 1. Stepwise screening procedure of the fungal isolate collection for potential microbial antagonists (Modified from: Köhl et al. [32]).

Table 1. Selection of fungal biocontrol candidates ~~obtained from different micro-compartments of tomato plants as described in the methods section. Five isolates were selected~~ **for *ad planta* trials with *P. infestans* on tomato plants**. Data on isolates inhibiting *A. solani, in vitro* are included.The putative genera were determined by BLAST analysis after partial sequencing of the ITS locus (approximately 560bp). Isolation parameters include media and temperature used for the isolation of fungi from plant samples. PDA: potato dextrose agar; OMA: oatmeal agar

	NCBI BLAST close relative (ITS loci)				Isolation parameters			Inhibitory activity on rye agar and PDA
**Isolate code**	~~**Probable**~~ ~~**G**~~**genus match** ~~**with (%) identity**~~	**Accession number**	**Sample source**	**Soil origin**	**Isolation temperature (°C)**	**Isolation Media**	**Spore count after 21 days of incubation at 25 °C on PDA**	***P. infestans***
~~Pf121~~	~~*Bionectria* sp.~~		~~Diseased rhizosphere~~	~~A~~	~~OMA~~	~~yes~~		
~~Pf 154~~	~~*Bionectria* sp.~~		~~Healthy rhizosphere~~	~~A~~	~~PDA~~	~~yes~~		
Pf101	*Chaetomium* sp.	PX060209	Healthy rhizosphere	B	21	PDA	1.8×10^6 spores/ml	yes

Formatted Table

Formatted: Font: Bold, Not Italic

Formatted: Font: Bold, Not Italic

Formatted: Font: Bold, Not Italic

Formatted: Font: Not Italic

Formatted: Superscript

~~Pf 179~~	~~*Cladosporium* sp.~~		~~Diseased leaves~~	~~A~~	~~PDA~~	~~yes~~		
~~Pf 242~~	~~*Cladosporium* sp.~~		~~Diseased phyllosphere~~	~~B~~	~~OMA~~	~~yes~~		
~~Pf 4~~	~~*Clonostachys* sp.~~		~~Healthy rhizosphere~~	~~A~~	~~OMA~~	~~yes~~		
~~Pf 22~~	~~*Clonostachys* sp.~~		~~Diseased rhizosphere~~	~~A~~	~~PDA~~	~~yes~~		
Pf 27	*Clonostachys* sp.	PX060190	Healthy rhizosphere	A	14	OMA	4.5×10^6 spores/ml	yes
Pf 45	*Ctenomyces* sp.	PX060194	Diseased rhizosphere	A	21	PDA	2.2×10^6 spores/ml	yes
~~Pf 56~~	~~*Furcasterigmium* sp.~~		~~Healthy rhizosphere~~	~~A~~	~~OMA~~	~~yes~~		
~~Pf 158~~	~~*Geomyces* sp.~~		~~Diseased rhizosphere~~	~~A~~	~~OMA~~	~~yes~~		

Pf 155	*Mortierella sp.*		Healthy rhizosphere	A	PDA	yes		
Pf 194	*Mortierella sp.*		Healthy rhizosphere	A	PDA	yes		
Pf 210	*Mortierella sp.*		Healthy rhizosphere	A	OMA	yes		
Pf 86	*Myrothecium sp.*		Diseased rhizosphere	A	OMA	yes		
Pf 31	*Paraphaeosphaeria* sp.		Healthy rhizosphere	A	PDA	yes		
Pf 23	*Pseudogymnoascus* sp.	PX060188	Diseased rhizosphere	A	21	OMA	2.6×10^6 spores/ml	yes
Pf 117	*Pseudogymnoascus* sp.		Healthy rhizosphere	A	PDA	yes		
Pf 133	*Talaromyces sp.*		Diseased rhizosphere	B	PDA	yes		

Pf 165	*Trametes* sp.		Diseased rhizosphere	A	OMA	yes		
Pf 131	*Trichoderma* sp.	PX060216	Diseased leaves	B	14	PDA	2.2×10^{6} spores/ml	yes
Pf 205	*Trichoderma* sp.		Healthy rhizosphere	B	PDA	yes		yes
Pf 218	*Trichoderma* sp.		Diseased rhizosphere	B	OMA	yes		yes
Pf 223	*Trichoderma* sp.		Healthy rhizosphere	B	OMA	yes		yes

Formatted Table

In vitro dual-culture assays of fungal isolates against *P. infestans* and *A. solani*

Two mycelial discs (5 mm) of 7-day-old agar cultures of the fungal isolates grown on PDA and 7-day-old cultures of *P. infestans* grown on ~~Rye~~ rye agar (200 g rye; 3 g glucose; 12 g agar; 1000 ml distilled water) were simultaneously inoculated 4 cm opposite each other on PDA in the first screening of 165 isolates (S11 Table) and on rye agar in the second screening of 24 isolates (S10 Table) in standard Petri dishes. The control plates were inoculated with *P. infestans* only. Three Petri dishes were prepared for each isolate. The plates were incubated at 25 °C for 6-7 days, and fungal pathogen growth reduction indicated by inhibition zones around *P. infestans* was recorded (yes- inhibition zone present and no- inhibition zone absent). Fungal isolates that grew over the pathogen were also recorded as (yes) showing potential inhibitory effect. The screening for *A. solani*, precultured on PDA medium was performed in the same way as described for *P. infestans*.

Testing disease suppressive effects of selected fungal isolates *ad planta*

Five fungal isolates, *Chaetomium* sp. (Pf101), *Trichoderma* sp. (Pf131), *Pseudogymnoascus* sp. (Pf23) *Chlonostachys* sp. (Pf27), and *Ctenomyces* sp. (Pf45), were selected from the characterized fungal collection for plant trials (Fig 1). The isolates were grown on PDA for 21 days at 25 °C for spore production. Tomato seeds (`Red Robin´, Weigelt Samen) were sown in a 3:1 mixture of substrate (ProLine Potgrond, Klasmann-Deilmann, Germany) and sand, respectively, in the greenhouse. The seedlings were transplanted into planting pots (8 x 8 x 8.5 cm) containing the same soil mixture, 14 days after sowing. The plants were maintained in the greenhouse with demand-oriented watering and weekly fertilizer application using 0.2% (w/v) Hakaphos blau (Compo Expert, Münster, Germany). From each of the above-mentioned fungal candidates, a spore suspension of 4×10^5 ml^{-1} was adjusted in distilled water. Five ml were sprayed on the leaves of 5-week-old plants. Plants were kept in the

dark overnight. On the next day, the plants were spray-inoculated with 5 ml 2 x 10^4 ml^{-1} conidia of *P. infestans.* The trays were covered with lids to ensure 100% humidity and left in the dark overnight. The plants were placed in the growth chamber (Grow Bank, Plant Climatics, Germany) set to 16 h/8 h light/darkness, day temperature 21 °C, night temperature 16 °C, day humidity 65%, and night humidity 75%. Percentage diseased leaf area was visually recorded 7 days after spraying with fungal biocontrol candidates as described by Drenker et al. [33]. Six tomato plants were used per treatment, and three independent replicates of the plant trials were done. All plant trials followed a randomized design.

Molecular identification of isolates with inhibitory effects against *P. infestans*

Fungal DNA was extracted from 7-day-old cultures grown on PDA at 28 °C using the DNeasy Plant Mini kit (Qiagen, Germany) following the manufacturer's protocol. DNA quality was determined spectrophotometrically (NanoDrop 2000c; peqLab Biotechnologie GmbH, Erlangen Germany). The Internal Transcribed Spacer (ITS) region (approximately 560bp) was amplified using primers ITS 1F-5'-TCCGTAGGTGAACCTGCGG-3', and ITS 4- 5'-TCCTCCGCTTATTGATATGC-3', as described in White et al. [36]. Briefly, a 25 µl PCR reaction mix was prepared for each sample, with reagents quantified as outlined by the manufacturer (Axon Labortechnik, Kaiserslautern). In the cases when PCR amplification was not successful, PCR reagents with a GoTaq® G2 Flexi DNA polymerase from Promega were used. The PCR cycling parameters were as follows: initial denaturation at 95 °C for 3 min; 40 cycles at 95 °C for 30 s (denaturation), 56 °C for 30 s (annealing), and 72 °C for 1 min (extension); final extension at 72 °C for 5 min. PCR products were purified using the ExoSAP-IT™ PCR Product Cleanup Reagent (Thermo Fisher Scientific) and 10 µl was used for sequencing (StarSEQ, Mainz), pre-mixed with ITS-1F. The Basic Local Alignment Search Tool (BLAST) was used to identify potential genus matches by comparing the sequences of the isolates against the

National Center for Biotechnology Information (NCBI) database (blastn, [37], and the most relevant hits based on the highest query coverage and percentage identity were recorded.

Molecular identification of *Chaetomium* species

The actual species of the *Chaetomium* isolate was evaluated by sequencing three identification loci according to Linkies et al. [38]. Internal transcribed spacer (primers ITS-1 and ITS-4 [36]; RNA polymerase II second largest subunit (primers RPB2AM-1bf (5'-CCA AGG TBT TYG TSA ACG G-3') and RPB2AM-7R (5'-GAA TRT TGG CCA TGG TRT CCA T-3') [39]; β-tubulin (primers T1 (5'-AACATGCGTGAGATTGTAAGT-3') and T22 (5'-TCTGGATGTTGTTGGGAATCC-3') [40] were employed with the following PCR conditions: Initial denaturation: 5 min at 95 °C, 40 cycles of denaturation: 30 s at 95 °C, 30 s of annealing at 55 °C and 45 s of extension at 72 °C, and a final extension of 5 min at 72 °C. The PCR product was visualized by 1% agarose gel electrophoresis. Cleaned PCR products using the ExoSAP-IT PCR Product Cleanup Reagent (Thermo Fisher, Berlin, Germany) were sequenced at StarSEQ (Mainz, Germany). Sequencing was performed in one direction and the species name was recorded as the highest BLAST hit (Table 2).

Table 2. Molecular identification of the *Chaetomium* isolate. A consensus sequence of three separate PCR reactions was examined. The top BLAST hits for all three examined coding regions showed a 100% match to *Chaetomium subaffine* in both the ITS and β-tubulin regions, and a 99.63% match in the RNA polymerase II second largest subunit region. The BLAST hit is shown with the highest probability for each of the three examined coding regions.

**Highest hit (NCBI BLAST)**	**Percentage identity**	**Locus**
Chaetomium subaffine	100%	ITS
Chaetomium acropullum	100%	ITS

~~*Chaetomium globosum*~~	~~100%~~	~~ITS~~
~~*Chaetomium subaffine*~~	~~100%~~	~~β-tubulin~~
~~*Chaetomium subaffine*~~	~~99.63%~~	~~RNA polymerase II second largest subunit~~

DNA extraction and library preparation for the fungal microbiome analysis

Total DNA was extracted from 0.25 mg of samples of the phyllosphere, endosphere, and rhizosphere from both soil origins and both healthy and diseased plants in four replicates (48 samples in total) using the DNeasy Powersoil Pro kit (Qiagen, Germany) following the manufacturer's protocol. DNA yield and quality were checked photometrically (NanoDrop 2000c; peqLab Biotechnologie GmbH, Erlangen Germany) and samples were stored at -20 °C. Library construction and high-throughput amplicon sequencing of ITS region with primers ITS1F (5'-CTTGGTCATTTAGAGGAAGTAA-3' [41] and ITS2 (5'-GCTGCGTTCTTCATCGATGC-3' [36] was carried out by StarSEQ GmbH (Mainz, Germany). Illumina adapter sequences were added in a 1-step PCR approach. Libraries were normalized and sequenced on the Illumina MiSeq platform with a v3 Reagent Kit in 2 x 300 nt paired-end mode.

Analysis of ITS rDNA amplicon sequencing

Primers and adapters were removed from high-quality raw reads using the software Cutadapt (v.4.4, [42], implemented in the European Galaxy-Server [43](https://usegalaxy.eu/). Processing of paired-end reads was performed in R (v.4.2.1, [44] using the DADA2 pipeline (v.1.24.0, [45] following the adapted workflow for ITS sequences (DADA2 ITS Pipeline Workflow (1.8), https://benjjneb.github.io/dada2/ITS_workflow.html, accessed 25.1.2024). The obtained amplicon sequence variants (ASVs) were taxonomically assigned using NCBI BLAST (megablast, v.2.14.1, [37]

with e-value 0.001 and the identity cut-off of 80% against the UNITE database (version 9, [46] containing ITS sequences in a Galaxy workflow as described in Fernandez-Grecco et al. [47]. Sequences identified as not fungal (= other eukaryotes) and sequences with less than five reads were removed from the dataset. This resulted in a final number of 2,124 ASVs and on average 124,032 quality reads per sample from 3,962,154 raw reads. Rarefaction curves indicated sufficient sequencing depth (S1 Fig ~~S1~~). Raw reads are deposited at the Sequence Read Archive (https://www.ncbi.nlm.nih.gov/sra) under the BioProject accession number PRJNA1273108.

All scripts including detailed parameters and data to reproduce the figures for this study have been deposited in GitHub (https://github.com/TKuhl-Nagel/Phytophthora_challenged_tomato_microbiome.git).

Data analysis and statistics

Statistical analysis and data handling for ASVs was performed using R (v.4.2.1, [44]) with the R packages “tidyverse” (v.1.3.2, [48], “ggplot2” (v.3.5.1, [49]), “ggtext” (v.0.1.2, [50]), “dplyr” (v.1.1.4, [51]), “tibble” (v.3.2.1, [52]), “stringr” (v.1.5.1, [53]), “ARTool” (v.0.11.1, [54]), “vegan” (v.2.6-6.1, [55]) and “phyloseq” (v.1.44.0, [56]). To account for uneven sequencing depth, the dataset was rarefied by 1000 times random subsampling to the lowest number of reads (18,628 reads) (S1 Fig~~.~~) and all further analyses were performed with the rarefied dataset as recommended by Schloss [57]. Significant effects of micro-compartment, soil origin, and disease status on alpha-diversity indices (Richness, Evenness, Shannon, Simpson) were calculated using the non-parametric aligned rank ANOVA because residuals failed the normality criteria based on the Shapiro test in the previously performed 3-way-ANOVA (S1-S4 Tables ~~S1-S4~~). After normalizing the dataset to relative abundance (%) Bray-Curtis distance was used to estimate beta-diversity and visualized using

nonmetric multidimensional scaling (NMDS). The effects of micro-compartment, soil origin, and disease status on the fungal community composition were tested using PERMANOVA (10,000 permutations). Relative abundance was computed for each ASV within each sample by dividing ASV counts by the total count per sample. Square bar plots were generated to represent the most abundant taxa (phylum, genus, and ASV level) based on their relative abundance in the whole dataset. Differences in their relative abundances based on soil origin were calculated for each micro-compartment using ANCOM-BC2 (v2.1.2, [58]) with p-value correction via the Benjamini-Hochberg method. The data set was filtered for rows with 0 ASVs prior to ANCOM-BC2 (Analysis of Compositions of Microbiomes with Bias Correction) to reveal differentially abundant taxa considering the two soil origins. ANCOM-BC2 also calculates the bias-corrected log-fold change (LFC) for each taxon between the two soil conditions to confirm that any taxon found only in one condition and completely absent in the other is a structural zero (sz) rather than rarely detected low-abundant taxa. LFC represents the ratio of the abundance of taxon between two conditions on a logarithmic scale. Negative LFC values indicate higher abundance in Soil A, while positive values indicate higher abundance in Soil B. ASVs classified as structural zero are exclusively present in one soil origin and are represented by strongly negative LFC (approaching $-\infty$) and strongly positive LFC (approaching $+\infty$). In order to compare the isolated living fungal species to the fungal community of the rhizosphere, we mapped the ASV sequences (~250 bp) to the isolate sequences (~500 bp) with 100% identity and at least 50% query coverage using NCBI BLAST. Afterwards, isolates were evaluated for their presence among the most abundant taxa and/or the differentially abundant taxa in the microbial community. Statistics for *ad planta* experiments and generation of plots were performed in R (versiolastn 4.4.2). Data on diseased leaf area (%) were presented as mean values ± standard error (SEM) computed from 6 biological replicates. The *ad planta* inhibitory effect of the fungal candidates against *P. infestans* was evaluated using a two-way analysis of variance (ANOVA) as the data was normally distributed according to Shapiro-Wilk test results. Significant differences

between means were evaluated by Tukey's test at P ≤ 0.05. Plant experiments were conducted in three independent repetitions, analyzed separately. Raw data from the three plant trials, and the associated R scripts including detailed parameters and data to reproduce the figures for this study have been deposited in GitHub (https://github.com/TKuhl-Nagel/Phytophthora_challenged_tomato_microbiome.git).

Results

Comparison of soil properties from Wiesbaden and Rüsselsheim in the the Rhine-Main ~~region~~area of Germany

Both soils were loam soils with comparable measures in terms of physical properties including humus level and organic carbon content (Table ~~3~~2). Soil A had a slightly higher pH than soil B. While both soils were sufficiently supplied with macronutrients such as phosphorus, potassium, magnesium, and trace elements, they had low iron content. Tomato plants grown in soil A demonstrated a significant difference in weight between healthy and diseased conditions following 14 days of infection with *P. infestans* (Fig 2A), with diseased plants showing a significantly lower fresh weight. In soil B, there was no significant difference in fresh weight between healthy and diseased plants. However, healthy plants grown in both soil origins had comparable measures in weight. In addition, plants grown in both soils showed slight differences in the degree of symptom development upon inoculation with *P. infestans* (Fig 2B). The results indicate that the selected soils have comparable properties and are suitable as a resource for plant-beneficial fungi.

Table ~~3~~2: Evaluation of the physiochemical parameters for soil A and soil B. The soil samples were analyzed according to VD-Lufa methods [59].

**Soil sample**	**A**	**B**	**A**	**B**
Soil origin	Wiesbaden	Rüsselsheim		
**Soil physical properties**	**Measured value**		**Evaluation**	
Soil type	Loam	Loam	Heavy	Heavy
pH value	7.5	7.2	Increased	Sufficient
Humus class	H2	H2	Slightly humic	Slightly humic
Organic carbon	0.6 - < 1.2	0.6 - < 1.2	Slightly humic	Slightly humic
**Main nutrients**	**Measured value** (mg/100 g)		**Supply rate**	
P_2O_5	119	44.0	High	High
K_2O	34.5	36.0	Above requirement	Above requirement
MgO	145	39.4	High	Above requirement
**Trace elements**	**Measured value** (mg/kg)		**Supply rate**	
Boron	1.99	1.26	Above requirement	Above requirement
Copper	5.52	3.23	High	High
Iron	349	373	Low	Low

Zinc	30.5	10.8	High	High

Fig 2. Effect of *P. infestans* on tomato plants grown in two different soil origins (A and B), in terms of (A) plant fresh weight and (B) diseased leaf area (%). The two soils were collected from two organic growers from Domäne Mechthildshausen, Wiesbaden (soil A) and Solidarische Landwirtschaft, Rüsselsheim (soil B). Plants were inoculated with *P. infestans* conidial suspension (2×10^4 conidia/ml) 5 weeks after sowing. Tomato plants inoculated with distilled water served as the healthy control. Four tomato plants were used for each treatment (n=4). Data were collected 14 days after inoculation with *P. infestans*. Means were compared with two-way ANOVA ($p \leq 0.01$) followed by Tukey's post-hoc test ($p \leq 0.05$). Significant differences are shown by letters (a, b).

Characterization of the fungal culture collection derived from tomato rhizosphere and phyllosphere

The isolation process of fungi from different samples yielded 246 isolates (Fig 1). In a stepwise screening approach described in the methods section, the number was reduced to 61 isolates (S9 Table S9) with promising characteristics as biocontrol agents against *P. infestans*. The *in vitro* antagonistic activity of the fungal isolates against *P. infestans* served as one selection criterion (Fig 3). Additionally, parameters that are advantageous for later product development and approval or registration as a biocontrol product were also taken into account, namely the inability to grow at 37 °C, easy spore induction, and ability to survive on standard growth media. Out of the 61 identified isolates with antagonistic effects against *P. infestans*, the rhizosphere yielded 45 fungal isolates, of which 24 and 21

isolates derived from healthy and diseased rhizosphere, respectively. Another 16 ~~16~~ isolates were recovered from the phyllosphere, of which eleven isolates derived from diseased phyllosphere compared to five isolates from healthy phyllosphere. All candidates were identified on the genus level by partial sequencing of the ITS locus.

Fig 3. Activity of selected fungal isolates against *P. infestans*. Dual-culture assays showing representative fungal candidates (right part of each Petri dish) and *P. infestans* (left part of each Petri dish). Assays were performed on PDA with both fungal plugs placed 4 cm apart on Petri dishes. The results were recorded after 7 days at 25 °C for n=3 plates.

The genus *Penicillium* constituted the largest share (27 isolates), followed by *Trichoderma* (6 isolates), *Agrostalagmus* (4 isolates), *Chlonostachys, Mortierella,* and *Pseudogymnoascus* (3 isolates each), (~~Fig 4~~S9 Table). All other genera were only present once or twice. Based on literature research, potential health concerns and plant pathogenic isolates, such as *Penicillium*, *Fusarium*, and *Acrostalagmus* were removed, ending up with 24 remaining candidates (Table 1). Among the 24 fungi, *Mortierella* sp., *Pseudogymnoascus* sp., *Trametes* sp., and *Trichoderma* sp. are notable for their potential to inhibit both *P. infestans* and *A. solani.* Among the antagonists tested for *ad planta* disease suppressive effects on *P. infestans* included *Pseudogymnoascus* sp. and *Trichoderma* sp.. However, no further tests were performed with *A. solani* as most of the antagonists showed weak inhibition potential via *in vitro* confrontation assays. Intriguingly, a majority of the selected fungi (17 isolates) originated from samples of tomato plants grown in soil A.

Fig 4. Taxonomic distribution of fungal isolates in the culture collection with *in vitro* activity against *P. infestans*. The genus determination was obtained through BLAST analysis after partial sequencing of the ITS locus. N=61

Fungal isolates show disease-suppressive effects *ad planta* against *P. infestans*

Five of the remaining 24 fungal candidates were selected for exemplary tomato plant trials evaluating suppressive characteristics against *P. infestans*. *Pseudogymnoascus* sp. (isolate Pf23), *Chlonostachys* sp. (Pf27), and *Ctenomyces* sp. (Pf45), *Chaetomium* sp. (Pf101), and *Trichoderma* sp. (Pf131), were chosen based on fast visible mycelial growth towards the edges of the Petri dish on standard media, abundant spore production (at least 4 x 10^5 ml^{-1} spores in standard fungal media (PDA)), and literature research. The isolates demonstrated varying levels of disease-suppressive potential in growth chamber plant trials (Fig 54). *Chaetomium* sp. (Pf101) significantly suppressed *P. infestans* across three independent replicates of the trial, as evidenced by the lower percentage of diseased leaf area compared to the positive control. *Chlonostachys* sp. (Pf27) significantly suppressed *P. infestans* in the first and second trials, whereas *Pseudogymnoascus* sp. (Pf23) significantly suppressed *P. infestans* in the second and third trials. *Ctenomyces* sp. (Pf45) was able to inhibit *P. infestans* once and *Trichoderma* sp. (Pf131) did not significantly inhibit symptoms of *P. infestans*, although the percentage of diseased leaf area was slightly lower compared to the positive control.

Fig ~~5~~4. Efficacy of selected fungal isolates against *P. infestans* on tomato plants across three trials. Spore suspensions (4×10^5 CFU/ml) prepared from *Chaetomium* sp. (Pf101), *Trichoderma* sp. (Pf131), *Pseudogymnoascus* sp. (Pf23), *Chlonostachys* sp. (Pf27), and *Ctenomyces* sp. (Pf45) were preventively applied on tomato leaves, 24 hours before inoculation with *P. infestans* (2×10^4 ml^{-1} conidia). Tomato plants inoculated with distilled water served as the negative control (Negative_control). Tomato plants inoculated with *P. infestans* only served as the positive control (Positive_control). Tomato plants treated with Cuprozin progress (0.52%) and inoculated with *P. infestans* served as the chemical control (Copper standard). Six tomato plants were used for each treatment (n=6). Percentage disease leaf area (%) was determined 7 days after inoculation with *P. infestans*. The letters above the boxes show significant differences across treatments ($p \leq 0.05$) according to Tukey HSD tests following ANOVA on diseased leaf area data. The three trials represent three independent replicates.

Soil origin influences the fungal community in tomato plants

The micro-compartment (endosphere, phyllosphere, rhizosphere), soil origin, disease status, and their interaction significantly influenced the alpha diversity of the fungal microbiome (ANOVA, Fig~~ure~~ ~~6A~~5A-D, S1-S4 Table~~s S1-4~~). Rhizosphere samples had the highest alpha-diversity indices indicating the presence of many species and with a rather homogeneous distribution compared to endosphere and phyllosphere. The disease status of the plant had a significant influence on the alpha-diversity only in the phyllosphere (ANOVA, S1-S4 Table~~s S1-4~~), which shows significant differences in the within-sample diversity between healthy and diseased plants mainly in soil B (Fig ~~6A~~5A-C). Besides, *P. infestans* had an effect on fungal species richness in the endosphere of plants grown in soil A, where infection reduced fungal species richness compared to healthy samples (Fig ~~6A~~5A). These results show

that leaf infection with *P. infestans* led to changes in the within-sample microbiome composition of the phyllosphere, although these changes are only significant in plants grown in soil B. In contrast, the endosphere and rhizosphere were mainly not significantly affected by microbial diversity after inoculation with *P. infestans*.

Fig ~~6~~5. The alpha-diversity indices species richness (A), Shannon diversity index (B), evenness (C), and Simpson index (D) of the fungal microbiome in samples from phyllosphere, endosphere, and rhizosphere from tomato plants grown in two soil origins (soil A and soil B). Plants were grown in two different soil origins (A and B) and were inoculated with *P. infestans* (diseased, (turquoise plots) or remained untreated (healthy, red plots) 14 days before sampling was done. Four tomato plants were used for each treatment (n=4). Values of alpha-diversity indices are shown, corresponding p-values are written above each comparison. Means were compared with a Kruskal Wallis test, with a significance threshold set at $p \leq 0.05$.

The micro-compartment, soil origin, and their interaction were identified as the driving factors of the fungal ß-diversity (PERMANOVA $p \leq 0.001$, S5 Table~~ S5~~, Fig ~~7~~6), whereas *P. infestans* infection had no significant influence on the fungal community composition. Phyllosphere samples clustered more distant from each other compared to rhizosphere and endosphere.

Fig ~~7~~6. Microbial beta-diversity of different tomato micro-compartments visualized using an NMDS plot. The fungal microbiome composition was determined using Bray–Curtis community dissimilarities. Plants were grown in two soil origins (A and B) and inoculated with *P. infestans* 14 d prior to sample collection from the rhizosphere, phyllosphere, and endosphere of healthy and diseased plants. ANOSIM was used to determine significant differences ($p \leq 0.001$, S5 Table~~ S5~~).

Based on these results we focused on the effect of soil origin on the fungal microbial community in the different micro-compartments because beta diversity analysis showed no significant difference between diseased and healthy plants. In each micro-compartment, we identified shared and unique taxa between the two soils (Fig 87), using ANCOM-BC2 (Analysis of Compositions of Microbiomes with Bias Correction). Rhizosphere samples of the two soil origins had overall more taxa compared to the phyllosphere and the endosphere, which confirms previous results. Interestingly, samples from soil B harbour more taxa compared to soil A across the three micro-compartments, indicating a soil-specific effect on fungal composition at the phylum, genus, and amplicon sequence variant (ASV) levels. At the same time, a substantial number of taxa were shared between plant samples from soil A and soil B across the three taxonomic levels (phylum, genus, ASV).

Fig 87: Fungal taxonomic distribution of the microbiome analyses at the phylum, genus, and amplicon sequence variant (ASV) levels in the rhizosphere, phyllosphere, and endosphere of soil A and soil B. The overlapping region represents taxa that are shared between soil A and soil B. Numbers inside each section represent the number of taxa at each level (phylum, genus, or ASV). The Venn diagram is based on the analysis of relative abundance with ANCOM-BC2.

Further analysis of the most abundant taxa in plant samples from the two soil origins showed that the phylum Ascomycota had the highest relative abundance in the whole data set (Fig 9A8A). Although other phyla showed variation between the two soil origins, only Basidiomycota, Rozellomycota, and Mortierellomycota significantly differed in abundance in individual micro-compartments. At the genus level, *Botryotrichum*, *Geosmithia*, *GS11_gen_Incertae_sedis*, and *Pseudopyrenochaeta* were more abundant in soil A, whereas *Aspergillus*, *Brunneomyces*, and *Verticillium* were more abundant in soil B in one or more micro-compartments (Fig 9B8B). *Cladosporium* and *Acremonium* were the top

abundant genera in the phyllosphere, while *Colletotrichum* and *Pseudopyrenochaeta* were the most abundant group in the endosphere. The rhizosphere had a high proportion for an unknown genus, *GS11_gen_Incertae_sedis*.

The most abundant ASVs in the whole dataset were from three phyla, with a majority from Ascomycota, followed by Olpidiomycota, and Rozellomycota (Fig ~~9C~~8C, Table ~~4~~3). The differentially abundant ASVs between the two soil origins across the three micro-compartments were linked to *Cladosporium*, *Geosmithia*, *Olpidium*, *Pseudopyrenochaeta*, *Verticillium*, and unknown genus (*GS11_gen_Incertae_sedis*) (Fig ~~9C~~8C, Table ~~4~~3).

Fig ~~9~~8. Diversity of the most abundant fungal taxa at phylum (A), genus (B), and (C) ASV levels between plants grown in soil A and soil B across the three micro-compartments (endosphere, phyllosphere, and rhizosphere). Others represent all taxa with less than 9%, 6%, and 5.5% abundance at phylum, genus, and ASV levels, respectively. Bars represent the mean relative abundance (%) of each phylum genus, and ASV. Error bars indicate standard deviation. Asterisks indicate a significant difference in abundance between samples from soil origins A and soil B inferred at p-value ≤ 0.05 according to Benjamini-Hochberg (BH) correction, which controls for false discovery rate (FDR) analyzed using ANCOM-BC2 (Analysis of Compositions of Microbiomes with Bias Correction).

Comparison of culture collection and fungal microbiome composition

In order to identify overlaps between the culture collection of living isolates and the microbiome analysis, we mapped ITS sequences of selected fungal isolates that demonstrated *in vitro* inhibitory effects against *P. infestans* to fungal ASVs in the microbiome of tomato plants grown in soils A and B. Of the 61 fungal isolates, 42 (*Penicillium* (21), *Trichoderma* (5), *Acrostalagmus* (4), *Clonostachys*

(2), *Pseudogymnoascus* (2), *Cladosporium* (2), *Bionectriaceae* (2), *Ctenomyces* (2), *Chaetomium* (1) and *Humicola* (1)) showed 100% identity with the ASV sequences.

Cladosporium linked to isolates Pf179 and Pf242 from our culture collection was the only genus detected among the top 15 ASVs in terms of relative abundance (Table ~~4~~3). Of the ASVs that matched our fungal collection, only ASV14 identified as *Cladosporium* sp. was differentially abundant in the rhizosphere between the two soil origins (Fig ~~9C~~8C, Table ~~4~~3), with higher abundance in soil A. The matching ASVs were further evaluated for exclusive presence (also referred to as structural zeros) in plant samples from either soil origin. Intriguingly, ASVs identified as the genera *Mortierella*, *Ctenomyces*, and *Pseudogymnoascus* were exclusively present in the rhizosphere of plant samples from soil A, whereas ASVs identified as the genus *Chaetomium* were exclusively found in samples of plants grown in soil B (S6 Table~~ S6~~). Phyllosphere and endosphere samples showed a similar pattern of exclusive taxonomic presence, with plants cultivated in soil A enriched with ASVs identified as the genus *Ctenomyces* and those from plants grown in soil B enriched solely with ASVs identified as *Chaetomium* (S7-S8 Table~~ S7–S8~~).

Table ~~4~~3: Taxonomic affiliation ~~(phylum and species levels)~~ of the top most abundant fungal ASVs in the microbiome of tomato plants in the whole data set. The ASVs were taxonomically assigned using NCBI BLAST (megablast, v.2.14.1) with an e-value of 0.001 and the identity cut-off of 80% against the UNITE database (version 9, [46]) containing ITS sequences in a Galaxy workflow.

**ASVid**	**Taxonomic affiliation at ~~Phylum~~phylum level**	**Taxonomic affiliation at genus level and potential ~~S~~species**
ASV1	Ascomycota	*Cladosporium sphaerospermum*
ASV2	Ascomycota	*Plectosphaerella ramiseptata*

Formatted Table

ASV3	Ascomycota	*Colletotrichum coccodes*
ASV4	Ascomycota	*Acremonium sclerotigenum*
ASV5	Ascomycota	*Berkeleyomyces rouxiae*
ASV6	Rozellomycota	GS11_gen_Incertae_sedis
ASV7	Ascomycota	*Verticillium dahliae*
ASV8	Ascomycota	*Pseudopyrenochaeta lycopersici*
ASV10	Ascomycota	*Pseudopyrenochaeta terrestris*
ASV12	Olpidiomycota	*Olpidium* sp.
ASV13	Olpidiomycota	*Olpidium* sp.
ASV14	Ascomycota	*Cladosporium halotolerans*
ASV16	Ascomycota	*Geosmithia* sp.
ASV28	Ascomycota	*Cladosporium sphaerospermum*

Discussion

In times of increasing awareness of sustainable use of pesticides, microorganisms gain more and more importance as biocontrol agents of plant diseases [60,61]. Our study characterized cultivable fungi from various plant micro-compartments as the initial step towards discovering fungal biocontrol agents against *P. infestans*. Additionally, we employed a fungal microbiome analysis to evaluate the potential importance of our fungal candidates within the microbiome. The initial number of 246 isolates obtained from the phyllosphere, endosphere, and rhizosphere of tomato plants was reduced to 24 potential biocontrol candidates remaining for plant trials by utilization of the step-wise screening procedure described by Köhl et al. [32]. The multi-step framework employs several steps to screen a high number of isolates for candidates fulfilling requirements for antagonists in commercial use, for example regarding efficacy, ecology, production, safety, and environmental risks. Schisler and Slininger [62]

also included the above commercial parameters to select viable bacterial antagonists against *Gibberella pulicaris,* a potato dry rot causing pathogen.

The rhizosphere yielded more candidate isolates than the phyllosphere and endosphere, which concurs with previous findings that described the rhizosphere as a highly competitive habitat and a stable reservoir for beneficial microbes in terms of pathogen suppression, nutrient acquisition, and host defense functions [24,63]. Additionally, as a nutrient-rich environment, partly caused by plant root exudates [64], the rhizosphere supports a diverse microbial community, including beneficial fungi with potential biocontrol characteristics. Low microbe numbers recovered from the phyllosphere are linked to the specific nature of this habitat which is characterized by highly varying conditions that support only the survival of a limited number of microbes [65]. Recovering more fungal candidates from diseased phyllosphere samples suggests the enrichment of fungal microbial antagonists in pathogen-prevalent micro-compartments. Phyllosphere-associated microbes have been described with various functions, including plant growth promotion and pathogen inhibition [66], and nitrogen fixation [67], and can be causative agents for plant diseases [66]. The genera *Penicillium, Trichoderma, Chlonostachys, Mortierella,* and *Pseudogymnoascus* dominated our collection of potential microbial antagonists based on *in vitro* tests. A majority of these fungal groups have been adequately described as potential biocontrol agents. Culture filtrates of *Penicillium griseofulvum* demonstrated inhibitory effects on the growth of *P. infestans in vitro* [68]. But since *Penicillium* [69] species have been frequently reported as mycotoxin producers or as otherwise harmful in regard to food safety and consumer health [70], all *Penicillium* sp. isolates were eliminated from subsequent steps at an early screening stage. *Trichoderma* has been broadly investigated as a BCA owing to its ability to thrive in diverse soil habitats and outcompete pathogens for space and nutrients among other biocontrol mechanisms [71]. *Trichoderma* is also linked to other plant-beneficial functions such as phosphate solubilization and could be vital for the overall fitness of tomato plants through plant growth promotion [72]. Particular strains of *Chlonostachys rosea* [73], a tomato stem endophyte, have been reported to

significantly reduce early blight caused by *A. solani* [74] and *B. cinerea* [75] in tomatoes. Products based on *Trichoderma* sp. [76] and *C. rosea* [77] are approved as fungicides in Europe. Moreover, there is limited information on the isolation of *Pseudogymnoascus* species from tomato plants, but this fungal genus is among the most abundant taxa in tomato root microbiome through amplicon sequencing [78].

While *in vitro* effects of certain microbial species or strains may not necessarily be reflected in the *in vivo* effects, the five selected fungal candidates tested in *ad planta* assays showed varying disease-suppressive efficiency against *P. infestans*. The candidate antagonists from *Chaetomium* sp., *Chlonostachys* sp., and *Pseudogymnoascus* sp. demonstrated significant disease-suppressive effects in at least two separate plant trials, validating the feasibility of our approach in identifying new or improved biocontrol agents. It is worth noting that the findings on *Pseudogymnoascus* contribute substantially to the knowledge of this genus in the field of plant pathology as a potential biocontrol agent. *Chaetomium* species, including *Chaetomium cochliodes* and *Chaetomium aureum,* are known to produce antifungal secondary metabolites against tomato pathogens such as *A. solani*, *B. cinerea*, and *P. infestans* [38,79]. However, other species of *Chaetomium* induce human pathogenic effects linked to systemic infections and allergic reactions [80]. Our isolate was confirmed to be *Chaetomium subaffine* after sequencing three identification loci (as described in the methods). To the best of our knowledge, there is currently no data on potential health or environmental risks associated with *C. subaffine.* Liu et al. [81] revealed the potential mycolytic effects of *C. subaffine* LB-1 on phytopathogenic fungi *B. cinerea* and *A. solani* by altering their cell membrane and cell wall. Reduced biocontrol efficacy by other groups, including *Trichoderma sp*. (Pf131) and *Ctenomyces sp*. (Pf45) across trials could be linked to environmental or procedural effects. For instance, soil-originated beneficial microbes such as *Trichoderma* when introduced to the leaves and *vice versa* may not survive or could cause negative interactions with the native community of microorganisms on the leaf reducing

the efficacy [3]. Further processing steps such as drying or adding stabilizers might surely change the outcome but have not been investigated here. Different application methods such as the use of soil additives and fertilizers or a regular leaf application allowing for preventive and curative effects might also increase efficacy [82] but have not been tested yet.

High-resolution microbiome profiling techniques have improved the research on cultivable fungal species with plant-beneficial effects, providing crucial data for the targeted application of antagonists to ensure improved efficacy [83,84]. One example of this approach is demonstrated by Sahu et al. [85] who integrated culture methods and metabarcoding to profile the microbiome of the rice phyllosphere, revealing the key microbial communities for blast pathogen control. Similarly, we detected the presence of some of our fungal candidate antagonists for *P. infestans,* including *Cladosporium* among the highly abundant taxa and differential taxa in the tomato plant microbiome dataset. Although some *Cladosporium* species are plant pathogens [86,87], there are plant-beneficial species promoting growth[88,89]. This genus could reflect the core microbiome which has received a lot of interest in recent years, with multiple studies revealing core microorganisms, including bacteria, fungi, and archaea playing different roles in various plant hosts[90–92]. The dominance of specific fungal taxa, for instance, the exclusive presence of *Mortierella, Ctenomyces,* and *Pseudogymnoascus* in the rhizosphere samples from soil A and *Chaetomium* from rhizosphere samples in soil B may be a reflection of their beneficial activities to ensure a healthy plant microbiome [83]. In the rhizosphere, root exudates released due to pathogen invasion trigger plants to recruit microorganisms that are involved in specific functions, including direct suppression of the pathogens [93,94]. The endosphere represents the internal phytomicrobiome enriched with specifically adapted microorganisms that interact intimately [95]. The phyllosphere is linked to the atmosphere and interacts continuously with the air microbiome [96]. The fact that the fungal candidate antagonists are naturally present within the tomato plant and are effective against *P. infestans* highlights their potential as BCAs [97,98].

Therefore, knowledge of the ecological role of fungal biocontrol candidates in their native microbiomes can provide insights into the selection of improved and effective fungal agents against *P. infestans*. Enhanced knowledge of how pathogen infection of a host plant alters soil microbial communities may pave the way for the use of microbial antagonists in the long-term management of plant diseases [99]. Therefore, our approach to microbiome characterization provides a more complete picture of how *P. infestans* and distinct soil sources may influence the composition of the tomato fungal community. Although *P. infestans* did not alter the overall fungal microbiome composition of tomato plants, significant changes in fungal alpha diversity were evident in specific micro-compartments, particularly the phyllosphere. Previous research on *P. parasitica* (closely related to *P. infestans*) revealed changes in the bacterial microbiota in tomato roots upon infection, whereas the fungal microbiota remained relatively stable [100]. These changes in fungal composition could also be attributed to distinct soil physiochemical parameters as we cultivated tomatoes in soils from two different locations in the Rhine-Main area [101]. Studies have shown that pH plays a key role in determining the composition of bacteria and fungi in the rhizosphere and bulk soil [102,103]. Also, soils with sufficient nutrient amounts provide optimal habitat for beneficial microbial communities to outperform pathogens [104].

Taken together, we present a strategic approach that enhances the likelihood of identifying fungal candidates with both strong biocontrol potential and commercial viability. The tested fungal biocontrol candidates, including *Chaetomium* sp., *Chlonostachys* sp., and *Pseudogymnoascus* sp. isolated from different micro-compartments of tomato plants grown in two soil origins inhibited *P. infestans* and *A.solani in vitro* and reduced *P. infestans*-related disease symptoms *ad planta*. We have also demonstrated that *P. infestans* infection of tomato plants influences the fungal community composition at the micro-compartment level, particularly on the phyllosphere rather than on the overall plant microbiome. However, the effect of soil origin on fungal community composition is significant

considering the overall microbiome of tomato plants. The high abundance and differential abundance of specific genera, in our case *Cladosporium* indicates the potential beneficial role of this genus in tomato plant health as initial treatment showed no negative effects on the plant. In subsequent steps, we plan to test the suppressive effects of our *Chaetomium subaffine* in open-field plant trials. Other fungal candidates with variable efficacy against *P. infestans* will be subjected to optimization steps and used as consortia. Lastly, we intend to incorporate both curative and preventive application methods in future plant trials to achieve better disease-suppressive effects from the fungal candidates in a controlled growth room and greenhouse conditions. These findings improve our knowledge of the link between fungal biological control agents and plant microbiome, providing valuable ecological data in identifying effective antagonists suited for sustainable control of *P. infestans*.

Acknowledgements

The authors thank Anja Götzmann, Julia Herzberg, and Ramona Mittag for technical support and Julian Maier for assistance with the analysis of plant trial data. Christian Fetzer from Landesbetrieb Landwirtschaft Hessen (LLH) established the contact to the growers whose soil was used for the study, which is kindly acknowledged.

Funding

P.O. received funding from Deutscher Akademischer Austauschdienst (DAAD) doctoral scholarship (award no. 91832749). The funders did not contribute to the study design, data collection and analysis, decision to publish, or preparation of the manuscript.

Author Contributions

Conceptualization: Philemon Orwa, Johannes A. Jehle, Romano Mwirichia, Ada Linkies.

Data curation: Philemon Orwa, Theresa Kuhl-Nagel, Rosa Meinhold-Ernst, Arne Seyer.

Formal analysis: Philemon Orwa, Theresa Kuhl-Nagel.

Funding acquisition: Philemon Orwa, Johannes A. Jehle, Romano Mwirichia, Ada Linkies.

Investigation: Philemon Orwa, Rosa Meinhold-Ernst, Arne Seyer.

Methodology: Philemon Orwa, Theresa Kuhl-Nagel, Rosa Meinhold-Ernst, Arne Seyer.

Project administration: Johannes A. Jehle, Ada Linkies.

Resources: Johannes A. Jehle, Ada Linkies.

Supervision: Theresa Kuhl-Nagel, Johannes A. Jehle, Romano Mwirichia, Ada Linkies.

Visualization: Philemon Orwa, Theresa Kuhl-Nagel.

Writing – original draft: Philemon Orwa.

Writing – review & editing: Theresa Kuhl-Nagel, Johannes Romano Mwirichia, Ada Linkies.

References

1. Ali MY, Sina AAI, Khandker SS, Neesa L, Tanvir EM, Kabir A, et al. Nutritional composition and bioactive compounds in tomatoes and their impact on human health and disease: A review. Foods. 2021;10(1).

2. Panno S, Davino S, Caruso AG, Bertacca S, Crnogorac A, Mandić A, et al. A review of the most common and economically important diseases that undermine the cultivation of tomato crop in the mediterranean basin. Agronomy. 2021;11(11): 1–45.

3. Hashemi M, Tabet D, Sandroni M, Benavent-Celma C, Seematti J, Andersen CB, et al. The

hunt for sustainable biocontrol of oomycete plant pathogens, a case study of *Phytophthora infestans*. Fungal Biol Rev. 2022;40: 53–69.

4. Cooke LR, Schepers HTAM, Hermansen A, Bain RA, Bradshaw NJ, Ritchie F, et al. Epidemiology and integrated control of potato late blight in Europe. Potato Res. 2011;54(2): 183–222.
5. Lehsten V, Wiik L, Hannukkala A, Andreasson E, Chen D, Ou T, et al. Earlier occurrence and increased explanatory power of climate for the first incidence of potato late blight caused by *Phytophthora infestans* in Fennoscandia. PLoS One. 2017;12(5): 1–21.
6. Fry WE, Birch PRJ, Judelson HS, Grünwald NJ, Danies G, Everts KL, et al. Five reasons to consider *Phytophthora infestans* a reemerging pathogen. Phytopathology. 2015;105(7): 966–981.
7. Haverkort AJ, Struik PC, Visser RGF, Jacobsen E. Applied biotechnology to combat late blight in potato caused by *Phytophthora infestans*. Potato Res. 2009;52(3): 249–264.
8. Kamoun S, Furzer O, Jones JDG, Judelson HS, Ali GS, Dalio RJD, et al. The top 10 oomycete pathogens in molecular plant pathology. Mol Plant Pathol. 2015;16(4): 413–434.
9. Wan NF, Fu L, Dainese M, Kiær LP, Hu YQ, Xin F, et al. Pesticides have negative effects on non-target organisms. Nat Commun. 2025;16(1): 1–16.
10. Wang Y, Tyler BM, Wang Y. Defense and counterdefense during plant-pathogenic oomycete infection. Annu Rev Microbiol. 2019;73: 667–696.
11. European Parliament. Directive 2009/128/EC of the european parliament and the council of 21 October 2009 establishing a framework for community action to achieve the sustainable use of pesticides. October. 2009;309: 71–86.
12. Pieterse CMJ, Zamioudis C, Berendsen RL, Weller DM, Van Wees SCM, Bakker PAHM. Induced systemic resistance by beneficial microbes. Annu Rev Phytopathol. 2014;52: 347–375.

13. Spadaro D, Droby S. Development of biocontrol products for postharvest diseases of fruit: The importance of elucidating the mechanisms of action of yeast antagonists. Trends Food Sci Technol. 2016;47: 39–49.

14. Raaijmakers JM, Mazzola M. Diversity and natural functions of antibiotics produced by beneficial and plant pathogenic bacteria. Annu Rev Phytopathol. 2012;50: 403–424.

15. Khabbaz SE, Zhang L, Cáceres LA, Sumarah M, Wang A, Abbasi PA. Characterisation of antagonistic *Bacillus* and *Pseudomonas* strains for biocontrol potential and suppression of damping-off and root rot diseases. Ann Appl Biol. 2015;166(3): 456–471.

16. Chowdappa P, Mohan Kumar SP, Jyothi Lakshmi M, Upreti KK. Growth stimulation and induction of systemic resistance in tomato against early and late blight by *Bacillus subtilis* OTPB1 or *Trichoderma harzianum* OTPB3. Biol Control. 2013;65(1): 109–117.

17. De Silva NI, Brooks S, Lumyong S, Hyde KD. Use of endophytes as biocontrol agents. Fungal Biol Rev. 2019;33(2): 133–148.

18. Rai S, Solanki MK, Solanki AC, Surapathrudu K. Biocontrol potential of *Trichoderma* spp.: current understandings and future outlooks on molecular techniques. In: Ansari RA, Mahmood I, editors. Plant Health Under Biotic Stress: Volume 2: Microbial Interactions. Singapore: Springer Singapore; 2019. p. 129–160.

19. Luo C, He Y, Chen Y. Rhizosphere microbiome regulation: Unlocking the potential for plant growth. Curr Res Microb Sci. 2025;8: 100322.

20. Bakker PAHM, Pieterse CMJ, de Jonge R, Berendsen RL. The soil-borne legacy. Cell. 2018;172(6): 1178–1180.

21. Gush S, Lebre P, Couthino TA, Cowan DA, van der Waals JE. Disentangling shifts in the soil microbiome of potatoes infected with *Rhizoctonia solani* anastomosis group 3-pt in search of potential biocontrol agents. Phytobiomes J. 2024;8(2): 128–142.

22. Raaijmakers JM, Paulitz TC, Steinberg C, Alabouvette C, Moënne-Loccoz Y. The rhizosphere: A playground and battlefield for soilborne pathogens and beneficial microorganisms. Plant Soil. 2009;321(1–2): 341–361.

23. Jones DL, Hinsinger P. The rhizosphere: Complex by design. Plant Soil. 2008;312(1–2):1–6.

24. Berendsen RL, Pieterse CMJ, Bakker PAHM. The rhizosphere microbiome and plant health. Trends Plant Sci. 2012;17(8): 478–486.

25. Bakker PAHM, Berendsen RL, Doornbos RF, Wintermans PCA, Pieterse CMJ. The rhizosphere revisited: Root microbiomics. Front Plant Sci. 2013;4: 1–7.

26. Jayne B, Quigley M. Influence of arbuscular mycorrhiza on growth and reproductive response of plants under water deficit: a meta-analysis. Mycorrhiza. 2014;24(2): 109–119.

27. Baum C, El-Tohamy W, Gruda N. Increasing the productivity and product quality of vegetable crops using arbuscular mycorrhizal fungi: A review. Sci Hortic. 2015;187: 131–141.

28. El Komy MH, Saleh AA, Eranthodi A, Molan YY. Characterization of novel *Trichoderma asperellum* isolates to select effective biocontrol agents against tomato *Fusarium* wilt. Plant Pathol J. 2015;31(1): 50–60.

29. Treseder KK, Lennon JT. Fungal traits that drive ecosystem dynamics on land. Microbiol Mol Biol Rev. 2015;79(2): 243–262.

30. Brar B, Bala K, Singh B, Pardeep S, Sadh K, Singh J. Bio - boosting agriculture: Harnessing the potential of fungi - bacteria - plant synergies for crop improvement. Discov Plants. 2024; 1(1): 21.

31. Kim H -Y., Choi GJ, Lee HB, Lee S -W., Lim HK, Jang KS, et al. Some fungal endophytes from vegetable crops and their anti-oomycete activities against tomato late blight. Lett Appl Microbiol. 2007;44(3): 332–337.

32. Köhl J, Postma J, Nicot P, Ruocco M, Blum B. Stepwise screening of microorganisms for commercial use in biological control of plant-pathogenic fungi and bacteria. Biol Control.

2011;57(1): 1–12.

33. Drenker C, El Mazouar D, Bücker G, Weißhaupt S, Wienke E, Koch E, et al. Characterization of disease-suppressive isolate of *Lysobacter enzymogenes* with broad antagonistic activity against bacterial, oomycetal and fungal pathogens in different crops. Plants. 2023;12(3).

34. Wieland G, Neumann R, Backhaus H. Variation of microbial communities in soil, rhizosphere, and rhizoplane in response to crop species, soil type, and crop development. Appl Environ Microbiol. 2001;67(12): 5849–5854.

35. Semagn K. Leaf tissue sampling and dna extraction protocols. Molecular plant taxonomy: methods and protocols. Totowa, NJ: Humana Press; 2014: 53–67.

36. White TJ, Bruns T, Lee S, Taylor J. Amplification and direct sequencing of fungal ribosomal RNA genes for phylogenetics. PCR protocols. San Diego: Academic Press; 1990: 315–22.

37. Madden T. The BLAST sequence analysis tool. NCBI Handb. 2013;2(5): 425–436.

38. Linkies A, Jacob S, Zink P, Maschemer M, Maier W, Koch E. Characterization of cultural traits and fungicidal activity of strains belonging to the fungal genus *Chaetomium*. J Appl Microbiol. 2021;131(1): 375–391.

39. Miller AN, Huhndorf SM. Multi-gene phylogenies indicate ascomal wall morphology is a better predictor of phylogenetic relationships than ascospore morphology in the Sordariales (Ascomycota, Fungi). Mol Phylogenet Evol. 2005;35(1): 60–75.

40. O'Donnell K, Cigelnik E. Two divergent intragenomic rDNA ITS2 types within a monophyletic lineage of the fungus *Fusarium* are nonorthologous. Mol Phylogenet Evol. 1997;7(1): 103–116.

41. Gardes M, Bruns TD. ITS primers with enhanced specificity for basidiomycetes--application to the identification of mycorrhizae and rusts. Mol Ecol. 1993;2(2): 113–118.

42. Martin M. Cutadapt removes adapter sequences from high-throughput sequencing reads.

EMBnet J. 2011;17(1): 10–12.

43. Afgan E, Baker D, Batut B, van den Beek M, Bouvier D, Cech M, et al. The Galaxy platform for accessible, reproducible and collaborative biomedical analyses: 2018 update. Nucleic Acids Res. 2018;46(W1): W537–W544.

44. R Core Team. R: A language and environment for statistical computing. R Foundation for Statistical Computing. 2022.

45. Callahan BJ, McMurdie PJ, Rosen MJ, Han AW, Johnson AJA, Holmes SP. DADA2: High-resolution sample inference from Illumina amplicon data. Nat Methods. 2016;13(7): 581–583.

46. Abarenkov K, Nilsson RH, Larsson KH, Taylor AFS, May TW, Frøslev TG, et al. The UNITE database for molecular identification and taxonomic communication of fungi and other eukaryotes: sequences, taxa and classifications reconsidered. Nucleic Acids Res. 2024;52(D1): D791–797.

47. Fernandez-Gnecco G, Gégu L, Covacevich F, Consolo VF, Bouffaud ML, Buscot F, et al. Alone as effective as together: AMF and *Trichoderma* inoculation boost maize performance but differentially shape soil and rhizosphere microbiota. J Sustain Agric Environ. 2024;3(1):1–16.

48. Wickham H, Averick M, Bryan J, Chang W, McGowan L, François R, et al. Welcome to the Tidyverse. J Open Source Softw. 2019;4(43): 1686.

49. Wickham H. ggplot2: Elegant graphics for data analysis. 2016;

50. Wilke CO, Wienik BM. _ggtext: Improved text rendering support for 'ggplot2'_. R package version 0.1.2. 2022. Available from: https://wilkelab.org/ggtext/

51. Wickham H, François R, Henry L, Müller K. _dplyr: A grammar of data manipulation_. R package version 1.1.4. R Packag version. 2023;1(2).

52. Müller, K, Wickham H. _tibble: Simple data frames_. R package version 3.2.1. 2023; Available from: https://cran.r-project.org/package=tibble

53. Wickham H. _stringr: Simple, consistent wrappers for common string operations_. R package version 1.4.0. 2019.
54. Elkin LA, Kay M, Higgins JJ, Wobbrock JO. An aligned rank transform procedure for multifactor contrast tests. UIST 2021 - Proc 34th Annu ACM Symp User Interface Softw Technol. 2021;754–768.
55. Oksanen J, Simpson G, Blanchet F, Kindt R, Legendre P, Minchin P, et al. Vegan: community ecology package_. R package version 2.6-6.1. 2024.
56. McMurdie PJ, Holmes S. Phyloseq: An R Package for Reproducible Interactive Analysis and Graphics of Microbiome Census Data. PLoS One. 2013;8(4).
57. Schloss PD. Sequencing effort in amplicon sequence analyses. mSphere. 2024;0(0).
58. Lin H, Peddada S Das. Analysis of compositions of microbiomes with bias correction. Nat Commun. 2020;11(1): 3514.
59. VDLUFA. Phosphordüngung nach Bodenuntersuchung und Pflanzenbedarf. VDLUFA-Verlag Darmstadt; 2018.
60. Koch E, Ole Becker J, Berg G, Hauschild R, Jehle J, Köhl J, et al. Biocontrol of plant diseases is not an unsafe technology! J Plant Dis Prot. 2018;125: 121–125.
61. Pandey AK, Kumar A, Dinesh K, Varshney R, Dutta P. The hunt for beneficial fungi for tomato crop improvement – Advantages and perspectives. Plant Stress. 2022;6: 100-110.
62. Schisler DA, Slininger PJ. Microbial selection strategies that enhance the likelihood of developing commercial biological control products. J Ind Microbiol Biotechnol. 1997;19(3): 172–179.
63. Philippot L, Raaijmakers JM, Lemanceau P, van der Putten WH. Going back to the roots: the microbial ecology of the rhizosphere. Nat Rev Microbiol. 2013;11(11): 789–799.
64. Bhagat S, Shete P, Jain A. Plant health: Feedback effect of root exudates and rhizobiome

interactions. Rhizobiome Ecol Manag Appl. 2023;345–375.
65. Vorholt JA. Microbial life in the phyllosphere. Nat Rev Microbiol. 2012;10(12): 828–840.
66. Rastogi G, Coaker GL, Leveau JHJ. New insights into the structure and function of phyllosphere microbiota through high-throughput molecular approaches. FEMS Microbiol Lett. 2013;348(1): 1–10.
67. Fürnkranz M, Wanek W, Richter A, Abell G, Rasche F, Sessitsch A. Nitrogen fixation by phyllosphere bacteria associated with higher plants and their colonizing epiphytes of a tropical lowland rainforest of Costa Rica. ISME J. 2008;2(5): 561–570.
68. Lalaymia I, Naveau F, Arguelles Arias A, Ongena M, Picaud T, Declerck S, et al. Screening and efficacy evaluation of antagonistic fungi against *Phytophthora infestans* and combination with arbuscular mycorrhizal fungi for biocontrol of late blight in potato. Front Agron. 2022;4: 1–20.
69. Pitt JI. *Penicillium* | *Penicillium* and *Talaromyces*: Introduction. Encyclopedia of Food Microbiology. Second Edi. Oxford: Academic Press; 2014. 6–13.
70. Khan R, Anwar F, Ghazali FM. A comprehensive review of mycotoxins: Toxicology, detection, and effective mitigation approaches. Heliyon. 2024;10(8).
71. Harman GE, Howell CR, Viterbo A, Chet I, Lorito M. *Trichoderma* species-opportunistic, avirulent plant symbionts. Nat Rev Microbiol. 2004;2(1): 43–56.
72. Adnan M, Islam W, Shabbir A, Khan KA, Ghramh HA, Huang Z, et al. Plant defense against fungal pathogens by antagonistic fungi with *Trichoderma* in focus. Microb Pathog. 2019;129: 7–18.
73. Funck Jensen D, Dubey M, Jensen B, Karlsson M. *Clonostachys rosea* to control plant diseases. 2021; 429–72.
74. Egel DS, Hoagland L, Davis J, Marchino C, Bloomquist M. Efficacy of organic disease control products on common foliar diseases of tomato in field and greenhouse trials. Crop

Prot. 2019;122: 90–97.

75. Høyer AK, Jørgensen HJL, Amby DB, Jensen B. Endophytic colonization of tomato plants by the biological control agent *Clonostachys rosea*. Biol Integr Control plant Pathog IOBC-WPRS Bull. 2016;115: 101–106.

76. European Food SafetyAuthority. Conclusion on the peer review of the pesticide risk assessment of the active substance *Trichoderma gamsii* ICC080. EFSA J. 2015;13(4).

77. Arena M, Auteri D, Barmaz S, Bellisai G, Brancato A, Brocca D, et al. Peer review of the pesticide risk assessment of the active substance *Clonostachys rosea* strain J1446 (approved in Regulation (EU) No 540/2011 as *Gliocladium catenulatum* strain J1446). EFSA J. 2017;15(7).

78. Manzotti A, Bergna A, Burow M, Jørgensen HJL, Cernava T, Berg G, et al. Insights into the community structure and lifestyle of the fungal root endophytes of tomato by combining amplicon sequencing and isolation approaches with phytohormone profiling. FEMS Microbiol Ecol. 2020;96(5).

79. Ibrahim SRM, Mohamed SA, Sindi I, Mohamed GA. Biologically active secondary metabolites and biotechnological applications of species of the family Chaetomiaceae (Sordariales): an updated review from 2016 to 2021. Mycol Prog. 2021;20: 595–639.

80. Kedves O, Kocsubé S, Bata T, Andersson MA, Salo JM, Mikkola R, et al. *Chaetomium* and *Chaetomium*-like species from european indoor environments include D*ichotomopilus finlandicus* sp. Nov. Pathogens. 2021;10(9).

81. Liu C, Kan S, Zhou H, Zhang H, Chang Z. Mycolytic effect and underlying mechanism of *Chaetomium subaffine* LB-1 on phytopathogenic fungi via morphological, physio-biochemical and transcriptomic analysis. J Phytopathol. 2024;172(1): 1–12.

82. Stephan D, Schmitt A, Martins Carvalho S, Seddon B, Koch E. Evaluation of biocontrol preparations and plant extracts for the control of *Phytophthora infestans* on potato leaves. Eur

J Plant Pathol. 2005;112(3): 235–46.

83. Berg G, Rybakova D, Fischer D, Cernava T, Vergès MCC, Charles T, et al. Microbiome definition re-visited: old concepts and new challenges. Vol. 8, Microbiome. England; 2020. 103.

84. Gruet C, Muller D, Moënne-Loccoz Y. Significance of the diversification of wheat species for the assembly and functioning of the root-associated microbiome. Front Microbiol. 2022;12.

85. Sahu KP, Patel A, Kumar M, Sheoran N, Mehta S, Reddy B, et al. Integrated metabarcoding and culturomic-based microbiome profiling of rice phyllosphere reveal diverse and functional bacterial communities for blast disease suppression. Front Microbiol. 2021;12 :1–21.

86. Watson A, Napier T, Mazwan Muhammad R, Rozana Nik Mohd Masdek N, Zahrah Ponari S, Anak Makup J, et al. Diseases of cucurbit vegetables. Prime Fact 832. 2009;2–3.

87. Ogórek R, Lejman A, Pusz W, Miłuch A, Miodyńska P. Characteristics and taxonomy of *Cladosporium* fungi. Mikol Lek. 2012;19(2): 80–85.

88. Hamayun M, Afzal Khan S, Ahmad N, Tang DS, Kang SM, Na CI, et al. *Cladosporium sphaerospermum* as a new plant growth-promoting endophyte from the roots of *Glycine max* (L.) Merr. World J Microbiol Biotechnol. 2009;25: 627–632.

89. Sandoval-Denis M, Sutton DA, Martin-Vicente A, Cano-Lira JF, Wiederhold N, Guarro J, et al. *Cladosporium* species recovered from clinical samples in the United States. J Clin Microbiol. 2015;53(9): 2990–3000.

90. Shakya M, Gottel N, Castro H, Yang ZK, Gunter L, Labbé J, et al. A multifactor analysis of fungal and bacterial community structure in the root microbiome of mature populus deltoides trees. PLoS One. 2013;8(10).

91. Pfeiffer S, Mitter B, Oswald A, Schloter-Hai B, Schloter M, Declerck S, et al. Rhizosphere microbiomes of potato cultivated in the high Andes show stable and dynamic core microbiomes with different responses to plant development. FEMS Microbiol Ecol.

2016;93(2)

92. Taffner J, Cernava T, Erlacher A, Berg G. Novel insights into plant-associated archaea and their functioning in arugula (*Eruca sativa* Mill.). J Adv Res. 2019;19: 39–48.

93. Lemanceau P, Blouin M, Muller D, Moënne-Loccoz Y. Let the core microbiota be functional. Trends Plant Sci. 2017;22(7): 583–595.

94. Pascale A, Proietti S, Pantelides IS, Stringlis IA. Modulation of the root microbiome by plant molecules: the basis for targeted disease suppression and plant growth promotion. Front Plant Sci. 2020;10:1–23.

95. Berg G, Köberl M, Rybakova D, Müller H, Grosch R, Smalla K. Plant microbial diversity is suggested as the key to future biocontrol and health trends. FEMS Microbiol Ecol. 2017;93(5).

96. Lindow SE, Brandl MT. Microbiology of the phyllosphere. Appl Environ Microbiol. 2003;69(4): 1875–1883.

97. Ramudingana P, Mamphogoro TP, Kamutando CN, Maboko MM, Modika KY, Moloto KW, et al. Antagonistic potential of endophytic fungal isolates of tomato (*Solanum lycopersicum* L.) fruits against post-harvest disease-causing pathogens of tomatoes: An *in vitro* investigation. Fungal Biol. 2024;128(4): 1847–1858.

98. El-Hasan A, Ngatia G, Link TI, Voegele RT. Isolation, identification, and biocontrol potential of root fungal endophytes associated with solanaceous plants against potato late blight (*Phytophthora infestans*). Plants. 2022;11(12).

99. Peixoto RS, Voolstra CR, Sweet M, Duarte CM, Carvalho S, Villela H, et al. Harnessing the microbiome to prevent global biodiversity loss. Nat Microbiol. 2022;7(11): 1726–1735.

100. Larousse M, Rancurel C, Syska C, Palero F, Etienne C, Industri B, et al. Tomato root microbiota and *Phytophthora parasitica*-associated disease. Microbiome. 2017;5(1): 1–11.

101. Berg G, Smalla K. Plant species and soil type cooperatively shape the structure and function of microbial communities in the rhizosphere. FEMS Microbiol Ecol. 2009;68(1): 1–13.

102. Docherty KM, Borton HM, Espinosa N, Gebhardt M, Gil-Loaiza J, Gutknecht JLM, et al. Key edaphic properties largely explain temporal and geographic variation in soil microbial communities across four biomes. PLoS One. 2015;10(11): 1–23.

103. Zhang T, Wang NF, Liu HY, Zhang YQ, Yu LY. Soil pH is a key determinant of soil fungal community composition in the Ny-Ålesund Region, Svalbard (High Arctic). Front Microbiol. 2016;7: 1–10.

104. Bardgett RD, Van Der Putten WH. Belowground biodiversity and ecosystem functioning. Nature. 2014;515(7528): 505–511.

Supporting information

S1 Table. Non-parametric aligned-rank ANOVA results for species richness using the ARTool package (v.0.11.1).

S2 Table. Non-parametric aligned-rank ANOVA results for Shannon using the ARTool package (v.0.11.1).

S3 Table. Non-parametric aligned-rank ANOVA results for species evenness index using the ARTool package (v.0.11.1).

S4 Table. Non-parametric aligned-rank ANOVA results for the Simpson index using the ARTool package (v.0.11.1).

S5 Table. Permutation test results from Adonis2 for beta diversity analysis with Bray-Curtis dissimilarity.

S6 Table. ANCOM-BC2 results of differential abundance of fungal ASVs between soil A and soil B in rhizosphere samples based on Log Fold Change (LogFC) and structural zero analysis.

S7 Table. ANCOM-BC2 results of differential abundance of fungal ASVs between soil A and soil B in phyllosphere samples based on Log Fold Change (LogFC) and structural zero analysis.

S8 Table. ANCOM-BC2 results of differential abundance of fungal ASVs between soil A and soil B in endosphere samples based on Log Fold Change (LogFC) and structural zero analysis.

S9 Table. Total collection of fungal isolates inhibiting *P. infestans, in vitro*.

S10 Table. Collection of 61 identified fungal biocontrol candidates inhibiting *P. infestans* after eliminating potential plant and human pathogens based on literature search.

S11 Table. Collection of 165 unidentified fungal isolates after eliminating those growing at 37°C and non viable isolates.

S12 Table. Collection of 246 initial fungal isolates recovered from tomato plant tissues.

S1 Fig. Rarefaction curve of observed species richness after sequencing of ITS rRNA gene from the rhizosphere, endosphere, and phyllosphere samples of tomato plants grown in soil origins A and B.

We thank the editorial board of PLOS ONE for considering our work for publication and in particular thank both reviewers for careful revision of our manuscript and for the valuable comments. We see that our manuscript lacks clarity in many parts, in particular in the procedure of the screening process. We did our best to improve clarity in the written text and by adding more raw data in the supplemental part and by depositing raw data to a data repository. We also responded to all other comments raised by the reviewers and the editor. Below you find the detailed responses to all remarks. We are available for any further questions and remarks.
On behalf of all authors, Ada Linkies

1. Please ensure that your manuscript meets PLOS ONE's style requirements, including those for file naming. The PLOS ONE style templates can be found at

https://journals.plos.org/plosone/s/file?id=wjVg/PLOSOne_formatting_sample_main_body.pdf and

https://journals.plos.org/plosone/s/file?id=ba62/PLOSOne_formatting_sample_title_authors_affiliations.pdf

We carefully checked the manuscript in the context of formal requirements of PLOS ONE. We indeed found things that needed correction, some tables and figures were not named correctly. They are indicated in the document with track changes at several positions and indicated as such.

We also found several inconsistencies in the format of the references. They were all corrected and the references were re-linked. As they were changed in reference program, they cannot be found by track changes, but all references are correct now to meet the formatting requirements of PLOS ONE.

2. In your Methods section, please provide additional information regarding the permits you obtained for the work. Please ensure you have included the full name of the authority that approved the field site access and, if no permits were required, a brief statement explaining why.

Both sampling locations were from professional tomato growers. The contact to the growers and the corresponding field sites (soil A and B) were provided by Christian Fetzer, employee from the Landesbetrieb Landwirtschaft Hessen, a governmental educational and advisory institution of the State of Hesse, subordinate to the Hessian Ministry of Agriculture and Environment, Viticulture, Forestry, Hunting, and Home Affairs (HMLU). We added information in lines 110-114 and we added him to the acknowledgement section.

3. We note that the grant information you provided in the 'Funding Information' and 'Financial Disclosure' sections do not match.

We checked the funding information in the section in the manuscript an online in the funding section related to the manuscript data. They both are identical and correct. I do not find a Financial Disclosure section, only Funding Information online in the system.

When you resubmit, please ensure that you provide the correct grant numbers for the awards you received for your study in the ‘Funding Information’ section.

The grant number in the section `Funding Information´ in the manuscript is correct, it is 91832749 for Philemon Orwa by the Deutscher Akademischer Austauschdienst (DAAD). The other authors did not receive any further funding.

4. When completing the data availability statement of the submission form, you indicated that you will make your data available on acceptance. We strongly recommend all authors decide on a data sharing plan before acceptance, as the process can be lengthy and hold up publication timelines. Please note that, though access restrictions are acceptable now, your entire data will need to be made freely accessible if your manuscript is accepted for publication. This policy applies to all data except where public deposition would breach compliance with the protocol approved by your research ethics board. If you are unable to adhere to our open data policy, please kindly revise your statement to explain your reasoning and we will seek the editor's input on an exemption. Please be assured that, once you have provided your new statement, the assessment of your exemption will not hold up the peer review process.

Our data was already in parts (the microbiome-related work) uploaded to GitHub, which is made immediately accessible now. We also uploaded all other data, related to the work with the living organisms and related R scripts to GitHub with immediate access for the public. The indication is also changed to immediate access in the online portal for document submission. We also added information in the manuscript, in the methods section lines 366-369. We hope to now have done all needed to adhere to the open data policy of PLOS ONE. If that is not the case, please let us now as soon as possible, so we can fix the remaining questions.

5. If the reviewer comments include a recommendation to cite specific previously published works, please review and evaluate these publications to determine whether they are relevant and should be cited. There is no requirement to cite these works unless the editor has indicated otherwise.

The reviewers did not recommend any further publications to be cited.

Additional Editor Comments:

After carefully reviewing the manuscript, I recommend major revision as detailed in the attached comments, and request that the authors submit a thoroughly revised version for further consideration.

Reviewer #1: Dear Corresponding Author
I checked your paper and now I have some comments to improve it:
1) I strongly suggest to have a phylogenetic tree to identification of fungal isolates.

We sincerely thank reviewer 1 for taking his or her precious time to carefully read our manuscript and for the valuable comments for improvement.

We agree on most other of the comments, but here we do not want to add a phylogenetic tree of our fungal isolates. The reasons are: first we only sequenced with one primer and only get secured information on genus level. This would make a tree construction not very precise in our eyes. Secondly, the resulting tree with our samples would show them being randomly distributed mainly within the Ascomycota, which is logical as it is the biggest phylum within the fungi. Further, our tree would not be complete anyway, as we had to stop our selection at a certain point to not get too many isolates for further downstream processes. Taken together, in our eyes this would not add any more clarity or depths on the manuscript, compared to the text and figures, which are already present. We therefore did not agree on this desired change, but we agreed on the other comments, as you see below.

2) Please submit your sequences of the isolates into GenBank and receive accession numbers.

Thanks for the valuable comment, definitely the sequences should be uploaded. We did this for all the 61 fungal isolates that were analyses on the ITS locus. They have been submitted to NCBI Genbank and the accession numbers have been included in Table 1, S9 and S10 table. (line 221 and supplementary information).

3) Please remove Tables a and 2.

We assume the reviewer means Table 1 and 2.

We modified table 1 and removed the isolates that were not in our core selection of five for plant trials. Instead we included selection criteria such as temperature and spore count, and we added the accession numbers. The remaining samples from the original table 1 are now included in the supplementary information in S10 table.

Table 2 has been removed. The key information from the table has been harmonized into a paragraph in lines 291-293. The numbers of the following tables were changed accordingly, also in the text.

4) To inference of OTUs that you implied in Table 4, it is better you kepp the genus and species level into main text and leave others as suppl. mat.

We prefer to leave table 4 the way it is, but we modified the column description to increase clarity. We think that table 4 shows more clearly the most abundant ASVs compared to a written statement. Also it is easier to refer to a table 4 in the results description compared to a written sentence.

5) Please remove Figure 4.

Figure 4 has been removed as suggested. The names of the represented genera in Figure 4 are already listed in table S9, which is described as such in the manuscript. The numbers of all following figures have been changed accordingly.

Reviewer #2: Greetings. Management of late blight pathogen using beneficial fungal isolates are need of the hour but needs clarity in the following points

We thank reviewer 2 for the careful and intense review of our manuscript. This resulted in many changes, which added clarity to the description and presentation of our screening procedure with the living isolates. We understood that the way was described in a way that lacked clarity here and there. We added information at several positions in the introduction (lines 49-51), the methods section (e.g. between lines 184-212) and in the newly constructed or modified tables (mainly S10-S12 table).

1. No proper data for antagonistic activity and screening and final restriction to final four isolates

We did qualitative (yes/no) screening of initial antagonistic activity in dual cultures as described in the methods section 184-212. To add more clarity we added/modified table 1 and S9 table and created S10- S12 table. We also added text describing the selection procedure from our initial collection to the five final isolates used in plant trials. This was done between lines 184-212.

2. Whether these are growth promoters? provide answer with data

This is definitely a very useful suggestion. The screening process of 246 isolates was very time consuming regarding disease suppression and the other described parameters only. Therefore the analysis of growth promoting effects was not done in this analysis.

3. Only identification with ITS targeting 560 bp is not sufficient??

We used the very commonly used primers ITS1 and ITS4, as described in White et al. (1990). They amplify a fragment of 560 bp size from the internal spacer region, which resulted in genus names, as described in table 1, S9 and S10 table. For genus identification, a fragment size of 560 bp is definitely sufficient. For our screening purposes the genus level at this stage was sufficient, as we aimed in further reducing the number of isolates to a workable amount for plant trials by excluding isolates that might be potentially harmful for animals, humans or which might present plant pathogens. An identification at species level would have been to ambitious at this stage, as it would have required different primers, based on the individual genera detected. We did this at a later stage for the identification of our Chaetomium isolate, where we used primers being described for identification on species level for the genus Chaetomium.

Although we realized, that in S9 table the most probable species was mentioned. We removed them and kept only the genus name here.

4. There is no clarity, strong data for selecting the beneficial fungus??

We are sorry that our description of the selection procedure was not detailed and clear enough. As described above we added information in the methods section, between lines 184-212 and in tables S10-S12.

5. Late blight pathogen is continuously getting patho types?? no data on virulent nature, accession numbers??

Thanks for the information. We added the strain names for both A. solani and P. infestans in lines 119 and 200 in the methods section. They are both pathogenic on the tomato cultivars in our lab, `Red Robin´ and are commonly used for pathogenicity tests, as in Drenker et al., 2023.

Comments in the manuscript itself.

Thanks also for the very detailed comments put in the PDF document. We extracted them from the file and comment on them below.

Line 22: include data for individual isolates (referring to the antagonistic activity of all 246 isolates).

We summarized the raw data of the initial screening in S10-S12 tables, summarizing the results of the screening of all 246 isolates. And the procedure is explained in the methods section, lines 184-212.

Line 27: provide the evidences for selection of these 5 isolates.

We believe this is answered in the reply to comment 4 and in the reply to the comment one above.

Line 48: indicate the losses in quantity of *P. infestans* in the past decades

A sentence has been added in line 49-51 indicating the losses associated with P. infestans over the last decades.

Line 53: expansion is needed for first time, regarding mentioning of EU

The abbreviations EU have been expanded to European Union at first mention in line 55.

Line 101: variety or hybrid (Red Robin tomato)

Red Robin is a tomato variety cultivated in Germany; this has now been specific in line 103

Line 102: not clear about the term `organic open-soil tomato cultivation´

We meant natural soil in the greenhouse not to be confused with artificial soil substrates commercially available in the market. The phrase 'open-soil' has been deleted and the word 'natural' added to improve sentence clarity, line 103.

Line 104: no assumption is needed ??.

The word assumption has been deleted (line 106), as it is practically true that that the two soils having been used for tomato cultivation in the previous season are enriched with organisms supportive of tomato plant health.

Line 107: is it location (positions of sampling)

Yes. We changed the word for locations.

Line 109: what is it? (referring to soil A and B)

This refers to both sampling sites. They are introduced before as soil A and B. We added a few words to add clarity (line 116-117).

Line 112: add accession number of *P. infestans* isolate

It is added.

Line 113: minimum one lakh spores (referring to 7 ml of 2x 104 sporangia per plant)

We sprayed 7 ml of 2x 10^4 zoospores which is equivalent to 140,000 spores per plant to obtain close to 30% infection which was appropriate for the microbiome study.

Line 120: partial or full length (referring to ITS amplicon size)

We performed partial ITS amplicon sequencing commonly used for fungi barcoding. This has been updated in the manuscript accordingly. It was already described in the methods section that primers ITS1 and ITS4 were used, which are very commonly used primers for genotyping.

Line 179: is it correct (referring to elimination of isolates growing at 37 °C

Yes. This is correct. We incubated all of the 246 isolates that we obtained from tomato tissues at 37 °C and those that grew were not considered for subsequent tests. This data has been provided in S11 and S12 Table.

Line 184: data is needed (referring to observed antagonistic effects)

This is shown in S11 table..

Line 185: add base pair (referring to ITS locus)

The base pair information has been updated in the manuscript as '(approximately 560bp)' because it varies for many fungal species. We deposited the sequences in Genbank and added the accession numbers in S9, S10 table.

Line 186: provide accession number (of the A. solani isolate)

We added the accession number (line 200).

Line 200: target base pairs (referring to ITS locus)

The target base pair is 560bp. This has been updated too.

Line 201: provide the result (regarding inhibitory activity against *P. infestans* and *A. solani*)

This data has now been provided in S10 Table and S9Table.

Line 241: 560 bp?? (referring to length of IST amplicon)

Yes 560bp is the ITS amplicon length. This is now indicated in brackets in line 265.

Line 342: is it place name? (referring to Wiesbaden and Rüsselsheim)

Yes. Wiesbaden and Rüsselsheim are the two locations from where we obtained soil from the growers. This has been clarified in lines 107-109 and the subtitle has also been modified to improve clarity (lines 374-375)

Line 378: ??? (referring to `another 16 isolates were…´)

There was a typing error, the repetitive 16 has been deleted in line 411.

Line 393: include the data (referring to `potential to inhibit both P. *infestans* and A. solani)

This data has now been provided in S10 Table and S9Table

Line 413: comment on no data related to `fast growth´.

We modified it to visible mycelial growth towards the edges of the Petri dish on standard media (line x). Not all isolates grew reliably and were discarded at a very early stage, but we did not monitor the growth rate at this screening state with over 200 isolates.

Line 413: is the correct term used (referring to `abundant´spore production)

We included the minimal required spore number that we used to decide on weather to continue with the isolate or not. This is included in the methods section and in the results, line 447.

Line 433: no data? (referring to the term `three independent replicates´).

We carried out three repetitions of the plant trials with 6 plants per replicate as described in the methods section in figure 5. Raw data and the R scripts for statistics of the data for diseased leaf area recorded following application of the 5 fungal antagonists have now also been uploaded in Github for the tree trials. The analysed data is visualized in the Box plot shown in Fig 5, now Fig 4.
