# Supplementary material for: Chaetomium, Chlonostachys, and Pseudogymnoascus isolates from tomato tissues significantly suppress Phytophthora infestans in tomato
Source: PLoS One. 2025 Oct 24;20(10):e0335007. doi: 10.1371/journal.pone.0335007 (PMC12551835; doi:10.1371/journal.pone.0335007)
Supplement: Attachment — Submitted filename: response to reviewers.docx [file pone.0335007.s015.docx]

*We thank the editorial board of PLOS ONE for considering our work for publication and in particular thank both reviewers for careful revision of our manuscript and for the valuable comments. We see that our manuscript lacks clarity in many parts, in particular in the procedure of the screening process. We did our best to improve clarity in the written text and by adding more raw data in the supplemental part and by depositing raw data to a data repository. We also responded to all other comments raised by the reviewers and the editor. Below you find the detailed responses to all remarks. We are available for any further questions and remarks.*

*On behalf of all authors, Ada Linkies*

1. Please ensure that your manuscript meets PLOS ONE's style requirements, including those for file naming. The PLOS ONE style templates can be found at

<https://journals.plos.org/plosone/s/file?id=wjVg/PLOSOne_formatting_sample_main_body.pdf> and

[https://journals.plos.org/plosone/s/file?id=ba62/PLOSOne_formatting_sample_title_authors_affiliations.pdf](https://track.editorialmanager.com/CL0/https:%2F%2Fjournals.plos.org%2Fplosone%2Fs%2Ffile%3Fid=ba62%2FPLOSOne_formatting_sample_title_authors_affiliations.pdf/1/010f019836dfe8a0-b286f709-42e8-4487-93e0-f926b2a8d45c-000000/pZloMkv90LL8ssnIhGa-Efn5dhJA2vZgefyX_f3jT70=220)

*We carefully checked the manuscript in the context of formal requirements of PLOS ONE. We indeed found things that needed correction, some tables and figures were not named correctly. They are indicated in the document with track changes at several positions and indicated as such.*

*We also found several inconsistencies in the format of the references. They were all corrected and the references were re-linked. As they were changed in reference program, they cannot be found by track changes, but all references are correct now to meet the formatting requirements of PLOS ONE.*

2. In your Methods section, please provide additional information regarding the permits you obtained for the work. Please ensure you have included the full name of the authority that approved the field site access and, if no permits were required, a brief statement explaining why.

*Both sampling locations were from professional tomato growers. The contact to the growers and the corresponding field sites (soil A and B) were provided by Christian Fetzer, employee from the Landesbetrieb Landwirtschaft Hessen, a governmental educational and advisory institution of the State of Hesse, subordinate to the Hessian Ministry of Agriculture and Environment, Viticulture, Forestry, Hunting, and Home Affairs (HMLU). We added information in lines 110-114 and we added him to the acknowledgement section.*

3. We note that the grant information you provided in the ‘Funding Information’ and ‘Financial Disclosure’ sections do not match.

*We checked the funding information in the section in the manuscript an online in the funding section related to the manuscript data. They both are identical and correct. I do not find a Financial Disclosure section, only Funding Information online in the system.*

When you resubmit, please ensure that you provide the correct grant numbers for the awards you received for your study in the ‘Funding Information’ section.

*The grant number in the section `Funding Information´ in the manuscript is correct, it is 91832749 for Philemon Orwa by the Deutscher Akademischer Austauschdienst (DAAD). The other authors did not receive any further funding.*

4. When completing the data availability statement of the submission form, you indicated that you will make your data available on acceptance. We strongly recommend all authors decide on a data sharing plan before acceptance, as the process can be lengthy and hold up publication timelines. Please note that, though access restrictions are acceptable now, your entire data will need to be made freely accessible if your manuscript is accepted for publication. This policy applies to all data except where public deposition would breach compliance with the protocol approved by your research ethics board. If you are unable to adhere to our open data policy, please kindly revise your statement to explain your reasoning and we will seek the editor's input on an exemption. Please be assured that, once you have provided your new statement, the assessment of your exemption will not hold up the peer review process.

*Our data was already in parts (the microbiome-related work) uploaded to GitHub, which is made immediately accessible now. We also uploaded all other data, related to the work with the living organisms and related R scripts to GitHub with immediate access for the public. The indication is also changed to immediate access in the online portal for document submission. We also added information in the manuscript, in the methods section lines 366-369. We hope to now have done all needed to adhere to the open data policy of PLOS ONE. If that is not the case, please let us now as soon as possible, so we can fix the remaining questions.*

5. If the reviewer comments include a recommendation to cite specific previously published works, please review and evaluate these publications to determine whether they are relevant and should be cited. There is no requirement to cite these works unless the editor has indicated otherwise.

*The reviewers did not recommend any further publications to be cited.*

Additional Editor Comments:

After carefully reviewing the manuscript, I recommend major revision as detailed in the attached comments, and request that the authors submit a thoroughly revised version for further consideration.

Reviewer #1: Dear Corresponding Author
I checked your paper and now I have some comments to improve it:
1) I strongly suggest to have a phylogenetic tree to identification of fungal isolates.

*We sincerely thank reviewer 1 for taking his or her precious time to carefully read our manuscript and for the valuable comments for improvement.*

*We agree on most other of the comments, but here we do not want to add a phylogenetic tree of our fungal isolates. The reasons are: first we only sequenced with one primer and only get secured information on genus level. This would make a tree construction not very precise in our eyes. Secondly, the resulting tree with our samples would show them being randomly distributed mainly within the Ascomycota, which is logical as it is the biggest phylum within the fungi. Further, our tree would not be complete anyway, as we had to stop our selection at a certain point to not get too many isolates for further downstream processes. Taken together, in our eyes this would not add any more clarity or depths on the manuscript, compared to the text and figures, which are already present. We therefore did not agree on this desired change, but we agreed on the other comments, as you see below.*

2) Please submit your sequences of the isolates into GenBank and receive accession numbers.

*Thanks for the valuable comment, definitely the sequences should be uploaded. We did this for all the 61 fungal isolates that were analyses on the ITS locus. They have been submitted to NCBI Genbank and the accession numbers have been included in Table 1, S9 and S10 table. (line 221 and supplementary information).*

3) Please remove Tables a and 2.

*We assume the reviewer means Table 1 and 2.*

*We modified table 1 and removed the isolates that were not in our core selection of five for plant trials. Instead we included selection criteria such as temperature and spore count, and we added the accession numbers. The remaining samples from the original table 1 are now included in the supplementary information in S10 table.*

*Table 2 has been removed. The key information from the table has been harmonized into a paragraph in lines 291-293. The numbers of the following tables were changed accordingly, also in the text.*

4) To inference of OTUs that you implied in Table 4, it is better you kepp the genus and species level into main text and leave others as suppl. mat.

*We prefer to leave table 4 the way it is, but we modified the column description to increase clarity. We think that table 4 shows more clearly the most abundant ASVs compared to a written statement. Also it is easier to refer to a table 4 in the results description compared to a written sentence.*

5) Please remove Figure 4.

*Figure 4 has been removed as suggested. The names of the represented genera in Figure 4 are already listed in table S9, which is described as such in the manuscript. The numbers of all following figures have been changed accordingly.*

Reviewer #2: Greetings. Management of late blight pathogen using beneficial fungal isolates are need of the hour but needs clarity in the following points

*We thank reviewer 2 for the careful and intense review of our manuscript. This resulted in many changes, which added clarity to the description and presentation of our screening procedure with the living isolates. We understood that the way was described in a way that lacked clarity here and there. We added information at several positions in the introduction (lines 49-51), the methods section (e.g. between lines 184-212) and in the newly constructed or modified tables (mainly S10-S12 table).*

1. No proper data for antagonistic activity and screening and final restriction to final four isolates

*We did qualitative (yes/no) screening of initial antagonistic activity in dual cultures as described in the methods section 184-212. To add more clarity we added/modified table 1 and S9 table and created S10- S12 table. We also added text describing the selection procedure from our initial collection to the five final isolates used in plant trials. This was done between lines 184-212.*

2. Whether these are growth promoters? provide answer with data

*This is definitely a very useful suggestion. The screening process of 246 isolates was very time consuming regarding disease suppression and the other described parameters only. Therefore the analysis of growth promoting effects was not done in this analysis.*

3. Only identification with ITS targeting 560 bp is not sufficient??

*We used the very commonly used primers ITS1 and ITS4, as described in White et al. (1990). They amplify a fragment of 560 bp size from the internal spacer region, which resulted in genus names, as described in table 1, S9 and S10 table. For genus identification, a fragment size of 560 bp is definitely sufficient. For our screening purposes the genus level at this stage was sufficient, as we aimed in further reducing the number of isolates to a workable amount for plant trials by excluding isolates that might be potentially harmful for animals, humans or which might present plant pathogens. An identification at species level would have been to ambitious at this stage, as it would have required different primers, based on the individual genera detected. We did this at a later stage for the identification of our Chaetomium isolate, where we used primers being described for identification on species level for the genus Chaetomium.*

*Although we realized, that in S9 table the most probable species was mentioned. We removed them and kept only the genus name here.*

4. There is no clarity, strong data for selecting the beneficial fungus??

*We are sorry that our description of the selection procedure was not detailed and clear enough. As described above we added information in the methods section, between lines 184-212 and in tables S10-S12*.

5. Late blight pathogen is continuously getting patho types?? no data on virulent nature, accession numbers??

*Thanks for the information. We added the strain names for both A. solani and P. infestans in lines 119 and 200 in the methods section. They are both pathogenic on the tomato cultivars in our lab, `Red Robin´ and are commonly used for pathogenicity tests, as in Drenker et al., 2023.*

Comments in the manuscript itself.

*Thanks also for the very detailed comments put in the PDF document. We extracted them from the file and comment on them below.*

Line 22: include data for individual isolates (referring to the antagonistic activity of all 246 isolates).

*We summarized the raw data of the initial screening in S10-S12 tables, summarizing the results of the screening of all 246 isolates. And the procedure is explained in the methods section, lines 184-212.*

Line 27: provide the evidences for selection of these 5 isolates.

*We believe this is answered in the reply to comment 4 and in the reply to the comment one above.*

Line 48: indicate the losses in quantity of *P. infestans* in the past decades

*A sentence has been added in line 49-51 indicating the losses associated with P. infestans over the last decades.*

Line 53: expansion is needed for first time, regarding mentioning of EU

*The abbreviations EU have been expanded to European Union at first mention in line 55.*

Line 101: variety or hybrid (Red Robin tomato)

*Red Robin is a tomato variety cultivated in Germany; this has now been specific in line 103*

Line 102: not clear about the term `organic open-soil tomato cultivation´

*We meant natural soil in the greenhouse not to be confused with artificial soil substrates commercially available in the market. The phrase ‘open-soil’ has been deleted and the word ‘natural’ added to improve sentence clarity, line 103.*

Line 104: no assumption is needed ??.

*The word assumption has been deleted (line 106), as it is practically true that that the two soils having been used for tomato cultivation in the previous season are enriched with organisms supportive of tomato plant health.*

Line 107: is it location (positions of sampling)

*Yes. We changed the word for locations.*

Line 109: what is it? (referring to soil A and B)

*This refers to both sampling sites. They are introduced before as soil A and B. We added a few words to add clarity (line 116-117).*

Line 112: add accession number of *P. infestans* isolate

*It is added.*

Line 113: minimum one lakh spores (referring to 7 ml of 2x 104 sporangia per plant)

*We sprayed 7 ml of 2x 10^4^ zoospores which is equivalent to 140,000 spores per plant to obtain close to 30% infection which was appropriate for the microbiome study.*

Line 120: partial or full length (referring to ITS amplicon size)

*We performed partial ITS amplicon sequencing commonly used for fungi barcoding. This has been updated in the manuscript accordingly. It was already described in the methods section that primers ITS1 and ITS4 were used, which are very commonly used primers for genotyping.*

Line 179: is it correct (referring to elimination of isolates growing at 37 °C

*Yes. This is correct. We incubated all of the 246 isolates that we obtained from tomato tissues at 37 °C and those that grew were not considered for subsequent tests. This data has been provided in S11 and S12 Table.*

Line 184: data is needed (referring to observed antagonistic effects)

*This is shown in S11 table..*

Line 185: add base pair (referring to ITS locus)

*The base pair information has been updated in the manuscript as ‘(approximately 560bp)’ because it varies for many fungal species. We deposited the sequences in Genbank and added the accession numbers in S9, S10 table.*

Line 186: provide accession number ( of the A. solani isolate)

*We added the accession number (line 200).*

Line 200: target base pairs (referring to ITS locus)

*The target base pair is 560bp. This has been updated too.*

Line 201: provide the result (regarding inhibitory activity against *P. infestans* and *A. solani*)

*This data has now been provided in S10 Table and S9Table.*

Line 241: 560 bp?? (referring to length of IST amplicon)

*Yes 560bp is the ITS amplicon length. This is now indicated in brackets in line 265.*

Line 342: is it place name? (referring to Wiesbaden and Rüsselsheim)

*Yes. Wiesbaden and Rüsselsheim are the two locations from where we obtained soil from the growers. This has been clarified in lines 107-109 and the subtitle has also been modified to improve clarity (lines 374-375)*

Line 378: ??? (referring to `another 16 isolates were…´)

*There was a typing error, the repetitive 16 has been deleted in line 411.*

Line 393: include the data (referring to `potential to inhibit both P. *infestans* and A. solani)

*This data has now been provided in S10 Table and S9Table*

Line 413: comment on no data related to `fast growth´.

*We modified it to visible mycelial growth towards the edges of the Petri dish on standard media (line x). Not all isolates grew reliably and were discarded at a very early stage, but we did not monitor the growth rate at this screening state with over 200 isolates.*

Line 413: is the correct term used (referring to `abundant´spore production)

*We included the minimal required spore number that we used to decide on weather to continue with the isolate or not. This is included in the methods section and in the results, line 447.*

Line 433: no data? (referring to the term `three independent replicates´).

*We carried out three repetitions of the plant trials with 6 plants per replicate as described in the methods section in figure 5. Raw data and the R scripts for statistics of the data for diseased leaf area recorded following application of the 5 fungal antagonists have now also been uploaded in Github for the tree trials. The analysed data is visualized in the Box plot shown in Fig 5, now Fig 4.*
